# Associations between polymorphisms of *SLC22A7*, *NGFR*, *ARNTL* and *PPP2R2B* genes and Milk production traits in Chinese Holstein

**DOI:** 10.1186/s12863-021-01002-0

**Published:** 2021-11-03

**Authors:** Ruike Jia, Yihan Fu, Lingna Xu, Houcheng Li, Yanhua Li, Lin Liu, Zhu Ma, Dongxiao Sun, Bo Han

**Affiliations:** 1 Department of Animal Genetics and Breeding, College of Animal Science and Technology, Key Laboratory of Animal Genetics, Breeding and Reproduction of Ministry of Agriculture and Rural Affairs, National Engineering Laboratory for Animal Breeding, China Agricultural University, No. 2 Yuanmingyuan West Road, Haidian District, Beijing, 100193 China; 2 Beijing Dairy Cattle Center, Beijing, 100192 China

**Keywords:** Genetic effect, SNP, Milk yield and composition, Dairy cattle

## Abstract

**Background:**

Our preliminary work confirmed that, *SLC22A7* (solute carrier family 22 member 7), *NGFR* (nerve growth factor receptor), *ARNTL* (aryl hydrocarbon receptor nuclear translocator like) and *PPP2R2B* (protein phosphatase 2 regulatory subunit Bβ) genes were differentially expressed in dairy cows during different stages of lactation, and involved in the lipid metabolism through insulin, PI3K-Akt, MAPK, AMPK, mTOR, and PPAR signaling pathways, so we considered these four genes as the candidates affecting milk production traits. In this study, we detected polymorphisms of the four genes and verified their genetic effects on milk yield and composition traits in a Chinese Holstein cow population.

**Results:**

By resequencing the whole coding region and part of the flanking region of *SLC22A7*, *NGFR*, *ARNTL* and *PPP2R2B*, we totally found 20 SNPs, of which five were located in *SLC22A7*, eight in *NGFR*, three in *ARNTL*, and four in *PPP2R2B*. Using Haploview4.2, we found three haplotype blocks including five SNPs in *SLC22A7*, eight in *NGFR* and three in *ARNTL*. Single-SNP association analysis showed that 19 out of 20 SNPs were significantly associated with at least one of milk yield, fat yield, fat percentage, protein yield or protein percentage in the first and second lactations (*P* < 0.05). Haplotype-based association analysis showed that the three haplotypes were significantly associated with at least one of milk yield, fat yield, fat percentage, protein yield or protein percentage (*P* < 0.05). Further, we used SOPMA software to predict a SNP, 19:g.37095131C > T in *NGFR*, changed the structure of NGFR protein. In addition, we used Jaspar software to found that four SNPs, 19:g.37113872C > G,19:g.37113157C > T, and 19:g.37112276C > T in *NGFR* and 15:g.39320936A > G in *ARNTL*, could change the transcription factor binding sites and might affect the expression of the corresponding genes. These five SNPs might be the potential functional mutations for milk production traits in dairy cattle.

**Conclusions:**

In summary, we proved that *SLC22A7*, *NGFR*, *ARNTL* and *PPP2R2B* have significant genetic effects on milk production traits. The valuable SNPs can be used as candidate genetic markers for genomic selection of dairy cattle, and the effects of these SNPs on other traits need to be further verified.

**Supplementary Information:**

The online version contains supplementary material available at 10.1186/s12863-021-01002-0.

## Background

Milk and dairy products have long been regarded as good sources of nutrition, providing proteins, fatty acids, minerals and vitamins. Studies have shown that eating dairy products seems to be good for lowering blood pressure and low density lipoprotein, muscle exercise and preventing tooth decay, diabetes, cancer and obesity [1]. Milk production traits are the most important economic traits for dairy cattle breeding, including 305-day milk yield, fat yield, protein yield, fat percentage and protein percentage [2]. These traits are quantitative traits, controlled by minor polygenes and greatly affected by the environment, so the breeding work is more difficult.

Since the genomic selection (GS) was comprehensively applied in dairy cow breeding in developed countries in 2009, the early and accurate selection of young bulls has been realized, the generation interval has been greatly shortened, and the genetic progress of the population has been accelerated, thus the breeding cost has been significantly reduced. GS uses SNP markers to select target traits. Studies have shown that adding functional gene information with greater genetic effects of target traits to SNP marker data can improve the accuracy of genome breeding value prediction [3–5].

Therefore, in recent decades, quantitative trait locus (QTL) mapping, candidate gene analysis, genome-wide association analysis and high-throughput sequencing techniques have been widely used in dairy cow breeding to identify the functional elements [6–10]. Moreover, studies have shown that SNPs (single nucleotide polymorphisms) in the gene can significantly affect the milk production traits of dairy cows [11–15].

Previously, we identified a number of genes, miRNAs and lncRNAs that showed differentially expressed patterns in different lactation periods of Holstein, and found 12 genes, including *SLC22A7*, *NGFR*, *ARNTL*, and *PPP2R2B*, participating in the lipid metabolism through insulin, PI3K-Akt, MAPK, AMPK, mTOR, and PPAR signaling pathways, which were considered to be the promising candidates for milk production traits in dairy cattle; additionally, the four genes, were regulated by the same lncRNA that is worthy of further verification [16].

*SLC22A7* (solute carrier family 22 member 7) is an organic anion transporter, which is involved in the transport of cGMP, niacin and propionic acid [17–20]. Propionic acid can be effectively converted into glucose, and the gene may regulate cell metabolism through the transport of propionic acid in the kidney and liver, and ultimately affect the milk production traits of dairy cows [21, 22].

It is reported that *NGFR* (nerve growth factor receptor) is closely associated with fat accumulation and the thickness of subcutaneous fat in pigs [23, 24], and another study has shown that the *NGFR* gene can regulate the directional differentiation of pluripotent stem cells into skeletal muscle progenitor cells [25].

*ARNTL* (aryl hydrocarbon receptor nuclear translocator like, also known as *BMAL1*) gene, which belongs to the family of transcriptional regulatory factors, is the main transcriptional activator in mammals and plays an important role in regulating circadian rhythm [26, 27]. A study shows that the deletion of *ARNTL* in the adipocytes can disrupt the energy balance of mice and eventually lead to obesity [28].

The expression product of *PPP2R2B* (protein phosphatase 2 regulatory subunit Bβ) gene is a regulatory subunit of protein phosphatase 2A, which is widely found in neurons throughout the brain [29]. Studies have shown that *PPP2R2B* is associated with some human diseases, such as spinocerebellar ataxia, breast cancer and autoimmune diseases [30–32].

Based on the four candidate genes for milk production traits in dairy cows, the main goal of this study was to identify the SNPs of these four genes, analyze their genetic association with milk yield, fat yield, fat percentage, protein yield, and protein percentage, and evaluate whether they can be used for the genomic selection chip development. In addition, we will predict the potential biological structure and function of the identified SNPs, such as transcription factor binding site (TFBS) and secondary protein structure, and speculate the causal mutation for milk traits in dairy cattle.

## Result

### SNPs identification

**Fig. 1:**
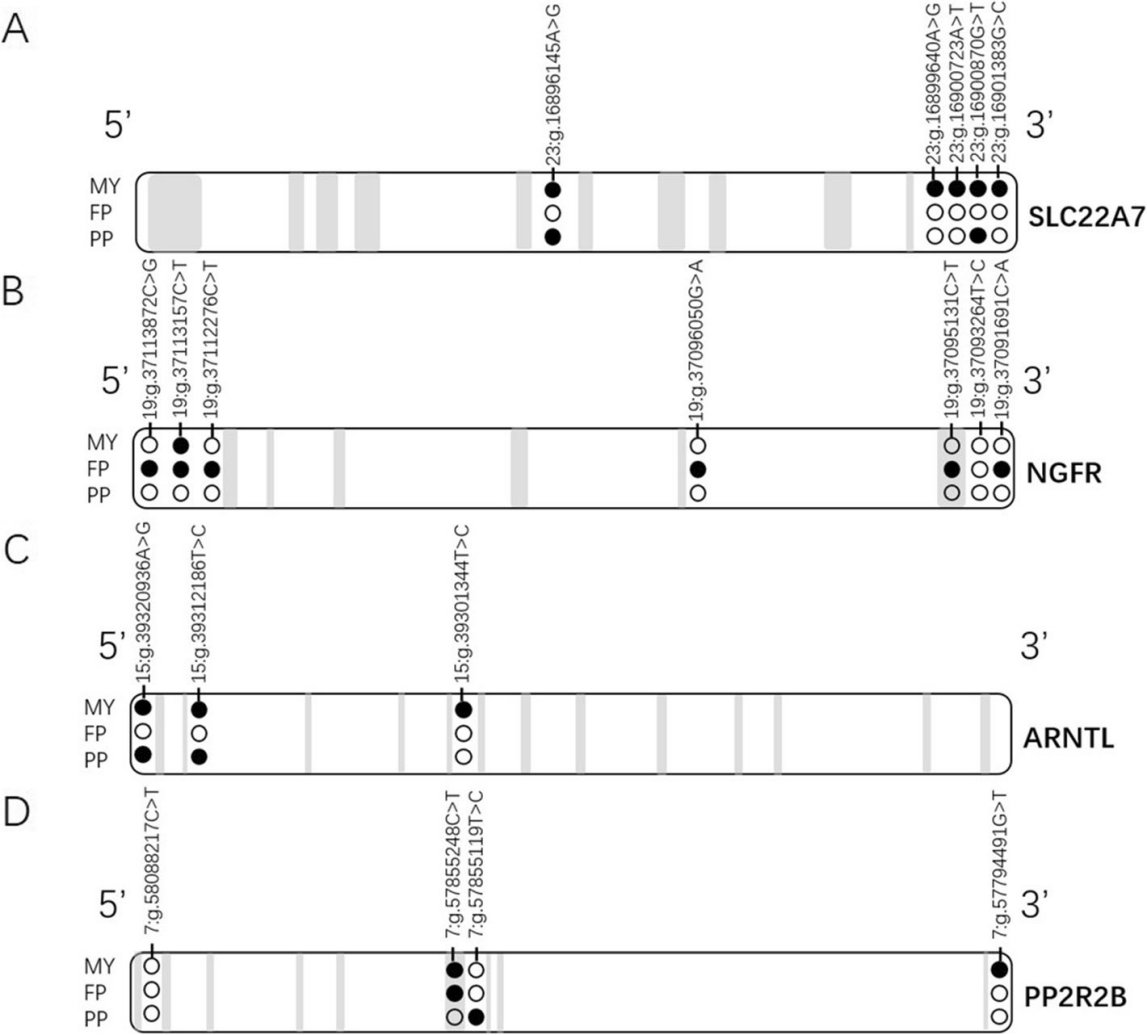
Location and association with milk yield, fat and protein percentage of SNPs in *SLC22A7* (A), *NGFR* (B), *ARNTL* (C) and *PPP2R2B* (D) genes. *MY* milk yield, *FP* fat percentage, *PP* protein percentage. Gray boxes represent exons, and solid black circles represent significant differences in first or second lactation

In this study, we found five, eight, three and four SNPs in *SLC22A7*, *NGFR*, *ARNTL* and *PPP2R2B* genes, respectively. In *SLC22A7*, one SNP was located in intron, one in 3′ untranslated region (UTR) and three in 3 ‘flanking region. In *NGFR*, there were three SNPs located in 5 ‘flanking region, one in intron, one in exon 6, two in 3′ untranslated region and one in 3 ‘flanking region. In *ARNTL*, one SNP was located in 5 ‘flanking region, and two in intron. In *PPP2R2B*, two SNPs were located in intron region, one in exon 6, and one in the 3 ‘flanking region (Table 1**;** Fig. 1).

**Table 1:** Detailed information about SNPs identified and their genotypic and allelic frequencies

Gene	SNP	RS ID	Gene region	Genotype	Genotypic frequency	Allele	Allelic frequency
SLC22A7	23:g.16896145A > G	rs135904081	intron	AA	0.0275	A	0.1642
				AG	0.2735	G	0.8358
				GG	0.6990		
	23:g.16899640A > G	rs133901529	3’UTR	AA	0.5238	A	0.7276
				AG	0.4076	G	0.2724
				GG	0.0686		
	23:g.16900723A > T	rs137472561	3’flanking region	AA	0.5238	A	0.7276
				AT	0.4076	T	0.2724
				TT	0.0686		
	23:g.16900870G > T	rs110672247	3’flanking region	GG	0.2967	G	0.5417
				GT	0.4900	T	0.4583
				TT	0.2133		
	23:g.16901383G > C	rs108993506	3’flanking region	CC	0.2957	C	0.5475
				CG	0.5037	G	0.4525
				GG	0.2006		
NGFR	19:g.37113872C > G	rs209326778	5’promoter region	CC	0.6484	C	0.8115
				CG	0.3263	G	0.1885
				GG	0.0253		
	19:g.37113157C > T	rs42707581	5’promoter region	CC	0.2587	C	0.5206
				CT	0.5238	T	0.4794
				TT	0.2175		
	19:g.37112276C > T	rs470795681	5’promoter region	CC	0.6473	C	0.8094
				CT	0.3242	T	0.1906
				TT	0.0285		
	19:g.37096050G > A	rs384321728	intron	AA	0.1595	A	0.4029
				AG	0.4868	G	0.5971
				GG	0.3537		
	19:g.37095131C > T	rs441506595	exon 6	CC	0.3516	C	0.5950
				CT	0.4868	T	0.4050
				TT	0.1616		
	19:g.37093264 T > C	rs43703926	3’UTR	CC	0.1700	C	0.4213
				CT	0.5026	T	0.5787
				TT	0.3273		
	19:g.37093189A > C	rs439860473	3’UTR	AA	1.0000	A	1.0000
					0.0000		
					0.0000		
	19:g.37091691C > A	rs382443341	3’flanking region	AA	0.1595	A	0.4023
				AC	0.4857	C	0.5977
				CC	0.3548		
ARNTL	15:g.39320936A > G	rs133103558	5’promoter region	AA	0.7318	A	0.8532
				AG	0.2429	G	0.1468
				GG	0.0253		
	15:g.39312186 T > C	rs137623467	intron	CC	0.1162	C	0.3654
				CT	0.4984	T	0.6346
				TT	0.3854		
	15:g.39301344 T > C	rs134613800	intron	CC	0.0412	C	0.2186
				CT	0.3548	T	0.7814
				TT	0.6040		
PPP2R2B	7:g.58088217C > T	rs380884981	intron	CC	0.7793	C	0.8849
				CT	0.2112	T	0.1151
				TT	0.0095		
	7:g.57855248C > T	rs457295594	exon 6	CC	0.8015	C	0.8976
				CT	0.1922	T	0.1024
				TT	0.0063		
	7:g.57855119 T > C	rs133004496	intron	CC	0.5924	C	0.7724
				CT	0.3601	T	0.2276
				TT	0.0475		
	7:g.57794491G > T	rs208484910	3’flanking region	GG	0.7012	G	0.8353
				GT	0.2682	T	0.1647
				TT	0.0306		

*Note*: *UTR* untranslated region

The SNP of *NGFR*, 19:g.37095131C > T, was a missense mutation, and when the allele was mutated from C to T, the amino acid changed from cysteine to tyrosine. Additionally, the genotypic and allelic frequencies of all the identified SNPs were summarized in Table 1.

### Association analyses between SNPs and the five Milk production traits

We analyzed the association between the 20 SNPs and five milk traits in dairy cows, including milk yield, fat yield, protein yield, fat percentage and protein percentage **(**Additional file 1**;** Fig. 1).

In *SLC22A7*, 23:g.16896145A > G was significantly associated with milk yield, protein yield and protein percentage in the first lactation, and milk yield, fat yield and protein yield in the second lactation. Two SNPs, 23:g.16899640A > G and 23:g.16900723A > T, were significantly associated with milk yield, fat yield and protein yield in the first lactation. The SNP 23:g.16900870G > T had significant associations on milk yield, protein yield in the first lactation, and milk yield, fat yield, protein yield and protein percentage in the second lactation. The 23:g.16901383G > C was significantly associated with milk yield, protein yield in the first lactation, and milk yield, fat yield and protein yield in the second lactation (*P* < 0.05).

In *NGFR*, 19:g.37113872C > G, 19:g.37096050G > A, 19:g.37095131C > T and 19:g.37091691C > A had significant associations with fat yield and fat percentage in the second lactation; 19:g.37113157C > T was significantly associated with milk yield, fat yield and fat percentage in the second lactation; and 19:g.37112276C > T had significant genetic effects on fat yield, fat percentage and protein yield in the second lactation (*P* < 0.05).

In *ARNTL*, 15:g.39320936A > G was significantly associated with milk yield, protein yield and protein percentage in the first lactation, and milk yield, fat yield and protein percentage in the second lactation; 15:g.39301344 T > C had significant effects on milk yield, fat yield, protein yield in the first and second lactations; and 15:g.39312186 T > C was significantly associated with milk yield, protein percentage in the first lactation, and milk yield, fat yield and protein yield in the second lactation (*P* < 0.05).

In *PPP2R2B*, 7:g.58088217C > T was significantly associated with fat yield and protein yield in the first lactation; 7:g.57855248C > T was significantly associated with milk yield, fat yield, protein yield in the first lactation and fat percentage in the second lactation; 7:g.57855119 T > C had significant effects on fat yield, protein yield, protein percentage in the first lactation and fat yield in the second lactation; 7:g.57794491G > T was significantly associated with fat yield in the first lactation, and milk yield and fat yield in the second lactation (*P* < 0.05).

In addition, the results of additive, dominant and substitution effects were shown in Additional file 2.

### Associations between haplotype blocks and the five Milk traits

**Fig. 2:**
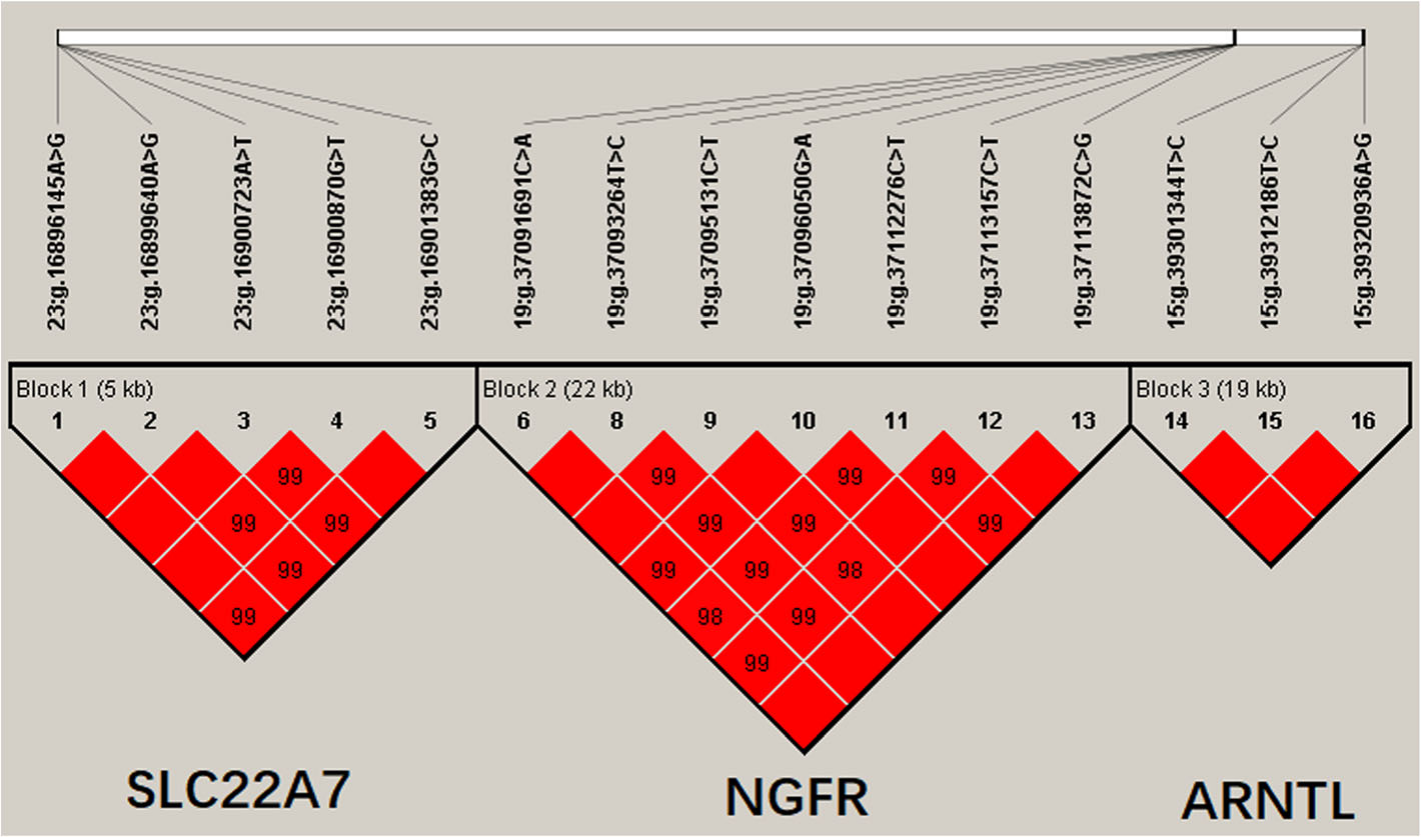
Linkage disequilibrium estimated between SNPs in *SLC22A7*, *NGFR* and *ARNTL* genes. The blocks indicate haplotype blocks and the text above the horizontal numbers is the SNP names. The values in the red boxes are pair-wise SNP correlations (D′), while bright red boxes without numbers indicate complete LD (D′ = 1)

We estimated the degree of linkage disequilibrium (LD) among the 20 identified SNPs in this study using Haploview4.2, and inferred three haplotype blocks, including five, seven and three SNPs of *SLC22A7*, *NGFR*, and *ARNTL* genes, respectively (Fig. 2; Block 1 (*SLC22A7*): D′ = 0.99–1.00; Block 2 (*NGFR*): D′ = 0.98–1.00; and Block 3 (*ARNTL*): D′ = 1).

In Block 1, the frequencies of four haplotypes, H1 (GAATC), H2 (GGTGG), H3 (AAAGG) and H4 (GAAGC), were 45.9, 27.2, 16.3 and 8.9%, respectively. In Block 2, four haplotypes were found, and the frequencies of H1 (CTCGCTC), H2 (ACTACCC), H3 (ACTATCG) and H4 (CTCGCCC), were 47.9, 21.3, 18.8, and 10%, respectively. In Block 3, the frequencies of H1 (TTA), H2 (CCA) and H3 (CTG) haplotypes were 63.5, 21.9 and 14.7%, respectively.

Moreover, we found that the haplotype combinations showed significant associations with 305-day milk yield, fat yield, fat percentage, protein yield, or protein percentage in the two lactations (*P* < 0.05; Additional file 3). Block 1 of *SLC22A7* was strongly associated with milk yield, fat yield, and protein yield in the first lactation, and milk yield and fat yield in the second lactation. Block 2 of *NGFR* had significant associations with milk yield, fat yield, and protein yield in the first lactation, and fat yield, fat percentage and protein yield in the second lactation. The Block 3 of *ARNTL* was significantly associated with milk yield, fat yield, protein yield and protein percentage in the first and second lactations.

### Functional variation prediction caused by SNPs

We predicted the TFBS changes of the four SNPs (19:g.37113872C > G, 19:g.37113157C > T, 19:g.37112276C > T and 15:g.39320936A > G) in the 5′ promoter region of *NGFR* and *ARNTL* genes by Jaspar software (Table 2), and found that all the four SNPs could change TFBSs.

**Table 2:** Transcription factor binding sites (TFBSs) prediction for *NGFR* and *ARNTL* genes

Gene	SNPs	Allele	Transcription factor	Relative score (≥0.80)	Predicted binding site sequence
*NGFR*	19:g.37113872C > G	C	SNAI2	0.86	CACAAGTAT
		G	SPI1	0.87	CAGAAG
			OSR2	0.90	TAACAGAAGTAT
	19:g.37113157C > T	T	STAT3	0.86	GGTCCTGGAAA
			FEV	0.88	CTGGAAAT
			ETV6	0.85	CCTGGAAATG
		C	GATA2	0.94	AGATG
			GATA1	0.93	AGATGT
	19:g.37112276C > T	T	MXI1	0.90	GGCACACGGG
		C	NRF1	0.85	GCGCACGGGCG
			SOHLH2	0.85	CGCACGGGCG
*ARNTL*	15:g.39320936A > G	G	DMRTA2	0.82	GTGTGCTACAGG
			OSR1	0.82	TGCTACAGGG
			PAX5	0.80	GTGTGCTACAGGGTGGGAA
			TFAP2C	0.80	TGCTACAGGGT
		A	FOXL1	0.82	GTGCTATA
			HOXC8	0.80	GCTATAGG
			MZF1	0.80	ATAGGGTGGG
			TBX6	0.80	AGTGTGCTAT

The allele C of 19:g.37113872C > G in *NGFR* created binding site (BS) for transcription factor SNAI2, and allele G created BS for SPI1 and OSR2. For 19:g.37113157C > T in *NGFR*, allele T invented BS for STAT3, FEV and ETV6, and allele C invented BS for GATA2 and GATA1. Allele T of 19:g.37112276C > T in *NGFR* created BS for MXI1, and allele C created BS for NRF1 and SOHLH2.

As for 15:g.39320936A > G in *ARNTL*, allele G created BS for transcription factors DMRTA2, OSR1, PAX5 and TFAP2C, and allele A created BS for FOXL1, HOXC8, MZF1 and TBX6.

Furthermore, the protein secondary structure variation of the missense mutation in *NGFR*, 19:g.37095131C > T, was predicted by SOPMA, and the results showed that α-helix varied from 22.61 to 23.78%, β-turn from 2.56 to 1.86%, and random coil from 65.50 to 65.03% when allele A mutated into G.

## Discussion

Our previous study considered *SLC22A7*, *NGFR*, *ARNTL* and *PPP2R2B* genes to be candidates to affect milk production traits in dairy cows [16]. In this study, we detected the polymorphisms of *SLC22A7*, *NGFR*, *ARNTL* and *PPP2R2B* genes, and found that there was a significant genetic association between the SNPs and haplotype blocks of these genes and milk production traits.

Transcription factors can affect gene expression by combining to TFBSs to regulate the transcription of target genes [33]. The SNP located at TFBS may affect the binding of transcription factors, resulting in differences in gene expression among individuals with different genotypes [34, 35]. In this study, we found the changes of TFBSs caused by the SNPs in 5 ‘flanking regions of *NGFR* and *ARNTL* genes. For 19:g.37113157C > T in *NGFR*, allele T invented BS for transcription factors STAT3, FEV and ETV6, and allele C invented BS for GATA2 and GATA1. In 15:g.39320936A > G of *ARNTL*, allele G created BS for DMRTA2, OSR1, PAX5 and TFAP2C, and allele A created BS for FOXL1, HOXC8, MZF1 and TBX6.

STAT3 is a transcription factor of the STAT family, which can be activated by tyrosine phosphorylation to inhibit the expression of gluconeogenic genes, thereby reducing blood glucose and maintaining blood glucose homeostasis [36]. The change of transcription factor FEV expression can affect the gene expression level of 5-hydroxytryptamine (5-HT) neurons in the central nervous system [37]. ETV6 is a transcriptional suppressor, which belongs to the ETS family and is highly conserved [38]. GATA2 inhibits the expression of genes related to cardiac development and up-regulates the important genes of hematopoietic differentiation in an indirect way [39]. GATA1 affects normal platelet production by regulating the expression of *NBEAL2* by long-distance enhancers [40]. A study has shown that DMRTA2 may suppress the expression of cyclin-dependent kinase inhibitor 2C to regulate spermatogenesis in zebrafish [41]. It has been found that OSR1 can inhibit the transcriptional activity of p53 and then inhibit the expression of oncogenes in renal cancer cells [42]. It is reported that the transcription factor PAX5 suppresses inappropriate genes in B cell lines and activates B cell-specific genes in B lymphocytes [43]. The down-regulation of tumor suppressor gene expression regulated by TFAP2C may be one of the carcinogenic causes of non-small cell lung cancer [44, 45]. FOXL1 can regulate the development of central nervous system by inhibiting the expression of Sonic Hedgehog protein in zebrafish [46]. HOXC8, as a transcriptional activator, induces the expression of transforming growth factor-β-1, which leads to the increase in the proliferation, anchorage-independent growth and migration of non-small cell lung cancer [47]. MZF1 has positive regulation on *CDC37* gene transcription by binding the regulatory sites, while MZF1 deletion reduces *CDC37* transcription and tumorigenicity of prostate cancer cells [48]. TBX6 is a member of the T-box transcription factor family, which regulates mouse embryonic development by promoting the expression of *Dll1* encoding Notch ligands [49].

These transcription factors can cause the activation or inhibition of gene expression, and in this study, they may interact to promote or suppress the expression of *NGFR* or *ARNTL* genes. Based on the phenotypic data of milk production traits with different genotypes, we found that the fat yield of genotype TT of 19:g.37113157C > T in *NGFR* was significantly higher than that of genotype CC, and the milk and protein yields of genotype AA in 15:g.39320936A > G of *ARNTL* were significantly higher than those of genotype GG. Thus, we speculated that the change of gene expression caused by SNP may be one of the reasons for the phenotypic changes of milk production traits in dairy cows.

In addition, we predicted the change of the protein secondary structure by the missense mutation (19:g.37095131C > T; cysteine (UGC) to tyrosine (UAC)) in exon 6 of the *NGFR* gene, and found that this missense mutation could change the protein secondary structure of NGFR. Generally speaking, the α-helix is located at the core of the protein and plays an important role in the conformational change of the protein [50]. The results of association analysis showed that this mutation was significantly associated with milk fat yield and fat percentage, so we speculated that this missense mutation, 19:g.37095131C > T, might affect the conformational stability of NGFR protein and ultimately influenced the milk fat traits of dairy cows, yet its biological function needs to be further studied.

## Conclusions

This study confirmed the significant genetic effects of *SLC22A7*, *NGFR*, *ARNTL* and *PPP2R2B* on milk production traits of Chinese Holstein cattle, and the valuable SNPs can be used as candidate genetic markers for molecular breeding of dairy cattle. Five SNPs were highlighted as the promising functional mutations for milk production traits, of which, 19:g.37113872C > G,19:g.37113157C > T and 19:g.37112276C > T in *NGFR* and 15:g.39320936A > G in *ARNTL*, might change the TFBSs to regulate expression of the corresponding gene, and the missense mutation of *NGFR*, 19:g.37095131C > T, could change the protein secondary structure. This study laid a foundation for further functional verification of *SLC22A7*, *NGFR*, *ARNTL* and *PPP2R2B*.

## Methods

### Animal selection and phenotypic data collection

In this study, we selected 947 daughters from 45 Chinese Holstein sire families in 22 farms of Beijing Shounong Animal Husbandry Development Co. LTD (Beijing, China) as the experimental population. The 45 sires were used for SNP identification and their daughters, 947 cows, were used for association analysis. Each sire family had an average of 21 daughters, and each cow had 3-generation pedigree information and Dairy Herd Improvement (DHI) records for milk yield, protein yield, protein percentage, fat yield and fat percentage (Additional file 4). The cows in each sire family were distributed in various dairy farms and maintained with the same feeding conditions.

We used the phenotypic data of 947 cows in the first lactation and 654 in the second lactation (293 cows merely completed the milking of first lactation) for the association analysis. The individual milk production phenotype of each cow was the data of the whole lactation period of the parity. The 305-day milk yield is calculated by multiplying the actual total milk yield by the corresponding estimated coefficient (Additional file 4). The 305-day milk fat and protein contents were obtained by multiplying the 305-day milk yield by the average milk fat and protein percentages, respectively. The average milk fat and protein percentages were the ratio of total milk fat and protein contents to total milk yield, respectively.

### Genomic DNA extraction

Frozen semen of the 45 bulls and blood samples of 947 cows were provided by Beijing Dairy Cattle Center (Beijing, China). We extracted genomic DNAs from the semen using salt-out procedure, and used a TIANamp Blood DNA Kit (Tiangen, Beijing, China) to extract DNAs from the blood. The quantity and quality of extracted DNAs (A260/A280 > 1.8) were determined by a NanoDrop2000 spectrophotometer (Thermo Science, Hudson, NH, USA) and gel electrophoresis (1.5%), respectively.

### SNP identification and linkage disequilibrium (LD) estimation

We designed primers (Additional file 5) with Primer3 (http://bioinfo.ut.ee/primer3-0.4.0/) to amplify all the coding regions and 2000 bp of 5′ and 3′ flanking regions of *SLC22A7*, *NGFR*, *ARNTL* and *PPP2R2B* genes based on the bovine reference genome sequences. Then the primers were synthesized by Beijing Genomics Institute (BGI, Beijing, China). We mixed the genomic DNAs of bull semen with the same amount, and then amplified them by PCR (Additional file 5). We used 2% gel electrophoresis to detect whether the PCR amplification products were qualified, and the qualified PCR products were sequenced.

After sequencing, we compared the sequences with the reference sequences (ARS-UCD1.2) on NCBI-BLAST (https://blast.ncbi.nlm. nih.gov/Blast.cgi) to identify the potential SNP. Subsequently, we genotyped the identified SNPs in 947 cows using Genotyping by Target Sequencing (GBTS) technology by Boruidi Biotechnology Co. Ltd. (Hebei, China). Further we calculated the allele frequency of each site and used the locus with minor allele frequency (MAF) > 0.05 for the following association analysis.

In addition, we used Haploview4.2 (Broad Institute of MIT and Harvard, Cambridge, MA, USA) to estimate the extent of linkage disequilibrium (LD) between the identified SNPs.

### Association analyses on Milk production traits

Associations between the SNPs or haplotype blocks and the milk production traits were analyzed using SAS 9.13 (SAS Institute Inc., Cary, NC, USA). The additive genetic relationship matrix A was constructed using the SAS, which was computed by tracing the pedigree back to three generations of 2827 involved individuals. Variance components were estimated based on the data of 30,000 Chinese Holstein cows in Beijing area by using the DMU package version 6.0 (University of Aarhus, Foulum, Denmark). Finally, the effects of the SNPs/haplotype blocks on first or second lactation milk production traits were estimated using the mixed procedure of SAS 9.13 software. With the following animal model, each trait was analyzed separately and each polymorphism/block was also fitted separately:

$$ Y=\upmu + hys+b\times M+G+a+e; $$

where Y is the phenotypic value of each trait of each cow; μ is the overall mean; hys is the fixed effect of farm (1–22: 22 farms), calving year (1–4: 2012–2015) and calving season (1: April–May; 2: June–August; 3: September–November and 4: December–March); M is the age of calving as a covariant, b is the regression coefficient of covariant M; G is the genotype or haplotype combination effect; a is the individual random additive genetic effect, the distribution is $\mathrm{N}\ \left(0,\mathbf{A}{\updelta}_a^2\right)$, **A** is a pedigree-based relationship matrix and the additive genetic variance is ${\updelta}_a^2$; and e is random residual, the distribution is $\mathrm{N}\ \left(0,\mathbf{I}{\updelta}_{\mathrm{e}}^2\right)$, the unit matrix I and the residual variance ${\updelta}_{\mathrm{e}}^2$. Bonferroni correction was carried out by multiple tests, the significance level was equal to the original *P* value divided by the number of genotype or haplotype combinations.

We also calculated the additive (a), dominant (d), and substitution (α) effects as follows: $a=\frac{AA- BB}{2}$; $\mathrm{d}= AB-\frac{AA+ BB}{2}$ ; *α* = *a* + *d*(*q* − *p*), where, AA, BB, and AB are the least square means of the milk production traits in the corresponding genotypes, p is the frequency of allele A, and q is the frequency of allele B.

### Biological function prediction

We used Jaspar software (http://jaspar.genereg.net/) to predict whether SNPs in the 5′ flanking region of *NGFR* and *ARNTL* genes changed the TFBS (relative score ≥ 0.80). We used SOPMA (https://prabi.ibcp.fr/htm/site/web/services/secondaryStructurePrediction#SOPMA) software to predict the effect of missense mutation on the secondary structure of protein, including α-helix, β-turn and random coil.

## Supplementary Information

**Additional file 1: Table S1**. Associations of 20 SNPs with milk yield and composition traits in Chinese Holstein cattle during first and second lactations.

**Additional file 2: Table S2**. Additive, dominant and allele substitution effects of 20 SNPs in *SLC22A7*, *NGFR*, *ARNTL* and *PPP2R2B* genes on milk yield and composition traits in Chinese Holstein cattle during two lactations.

**Additional file 3: Table S3**. Haplotypes analyses for *SLC22A7*, *NGFR* and *ARNTL* genes.

**Additional file 4: Table S4**. The phenotypic values for milk yield and composition in two lactations and pedigree information.

**Additional file 5: Table S5**. Primers and procedures for PCR used in SNPs identification of *SLC22A7*, *NGFR*, *ARNTL* and *PPP2R2B* genes.

## Data Availability

The information of genetic polymorphisms loci can be inquired on European Variation Archive (EVA; https://www.ebi.ac.uk/eva/) using the RS IDs (Table 1). The datasets generated during and/or analyzed during the current study are available in the article and its additional files.

## Abbreviations

ARNTL: Aryl hydrocarbon receptor nuclear translocator like
BS: Binding site
DHI: Dairy Herd Improvement
GS: Genomic selection
GBTS: Genotyping by Target Sequencing
5-HT: 5-hydroxytryptamine
NGFR: Nerve growth factor receptor
PPP2R2B: Protein phosphatase 2 regulatory subunit Bβ
QTL: Quantitative trait locus
SLC22A7: Solute carrier family 22 member 7
SNPs: Single nucleotide polymorphisms
TFBS: Transcription factor binding site
UTR: Untranslated region
