# Supplementary material for: Associations between polymorphisms of SLC22A7, NGFR, ARNTL and PPP2R2B genes and Milk production traits in Chinese Holstein
Source: BMC Genom Data. 2021 Nov 3;22:47. doi: 10.1186/s12863-021-01002-0 (PMC8567656; doi:10.1186/s12863-021-01002-0)
Supplement: Supplementary file 1 — Additional file 1: Table S1. Associations of 20 SNPs with milk yield and composition traits in Chinese Holstein cattle during first and second lactations. [file 12863_2021_1002_MOESM1_ESM.pdf]

Supplementary Information

Additional file 1: Table S1 Associations of 20 SNPs with milk yield and composition traits in Chinese Holstein cattle during first and second lactations.

Genes	SNPs	Lactation	Genotype (No.)	Milk yield (kg)	Fat yield (kg)	Fat percentage (%)	Protein yield (kg)	Protein percentage (%)
SLC22A7	23:g.16896145A>G	1	AA (26)	9797.11±159.19^{Aa}	332.72±6.52	3.42±0.06	291.38±4.75^{a}	2.98±0.02
			AG (259)	10330±69.71^{Bb}	340.71±3.04	3.32±0.03	303.77±2.22^{b}	2.95±0.01
			GG (662)	10246±60.60^{ABb}	338.52±2.71	3.33±0.03	303.04±1.97^{b}	2.97±0.01
		2	AA (20)	10286±181.21^{a}	372.28±7.42^{ab}	3.67±0.07	305.9±5.41^{ab}	2.99±0.03
			AG (177)	10803±78.36^{b}	387.18±3.40^{a}	3.59±0.03	318.2±2.48^{a}	2.96±0.01
			GG (458)	10602±63.59^{a}	380.31±2.86^{b}	3.61±0.03	312.81±2.08^{b}	2.97±0.01
	23:g.16899640A>G	1	AA (496)	10324±62.67^{Aa}	340.86±2.79^{a}	3.33±0.03	304.88±2.03^{Aa}	2.96±0.01
			AG (386)	10160±65.16^{Bb}	335.63±2.87^{b}	3.32±0.03	299.92±2.09^{Bb}	2.96±0.01
			GG (65)	10204±107.45^{ab}	343.28±4.49^{ab}	3.37±0.04	302.47±3.27^{ab}	2.97±0.02
		2	AA (343)	10668±68.26	382.53±3.03	3.60±0.03	314.87±2.21	2.96±0.01
			AG (271)	10624±70.22	380.44±3.1	3.61±0.03	313.57±2.26	2.97±0.01
			GG (41)	10682±132.42	388.16±5.49	3.65±0.05	312.21±4.00	2.94±0.02
	23:g.16900723A>T	1	AA (496)	10324±62.67^{Aa}	340.86±2.79^{a}	3.33±0.03	304.88±2.03^{Aa}	2.96±0.01
			AT (386)	10160±65.16^{Bb}	335.63±2.87^{b}	3.32±0.03	299.92±2.09^{Bb}	2.96±0.01
			TT (65)	10204±107.45^{ab}	343.28±4.49^{ab}	3.37±0.04	302.47±3.27^{ab}	2.97±0.02
		2	AA (343)	10668±68.26	382.53±3.03	3.60±0.03	314.87±2.21	2.96±0.01
			AT (271)	10624±70.22	380.44±3.10	3.61±0.03	313.57±2.26	2.97±0.01
			TT (41)	10682±132.42	388.16±5.49	3.65±0.05	312.21±4.00	2.94±0.02
	23:g.16900870G>T	1	GG (281)	10131±69.15^{Aa}	337.02±3.02	3.35±0.03	299.9±2.20^{Aa}	2.97±0.01
			GT (464)	10277±62.72^{ABb}	338.66±2.79	3.32±0.03	303.08±2.03^{ab}	2.96±0.01
			TT (202)	10366±76.28^{Bb}	342.51±3.30	3.33±0.03	306.31±2.40^{Bb}	2.97±0.01
		2	GG (188)	10602±77.17^{ab}	381±3.35^{ab}	3.62±0.03	314±2.44^{ab}	2.97±0.01
			GT (322)	10760±68.59^{Aa}	385.29±3.04^{a}	3.60±0.03	316.02±2.22^{a}	2.95±0.01
			TT (145)	10476±85.64^{Bb}	376.27±3.69^{b}	3.62±0.03	310.08±2.69^{b}	2.98±0.01
	23:g.16901383G>C	1	CC (280)	10364±71.22^{a}	341.25±3.11	3.32±0.03	306.42±2.27^{Aa}	2.97±0.01
			CG (477)	10234±62.19^{ab}	338.55±2.77	3.33±0.03	302.26±2.01^{ABb}	2.96±0.01
			GG (190)	10146±76.12^{b}	336.82±3.28	3.34±0.03	299.26±2.39^{Bb}	2.96±0.01

		2	CC (194)	10542±78.77^{a}	377.26±3.43^{Aa}	3.60±0.03	311.5±2.50^{a}	2.97±0.01
			CG (334)	10754±67.72^{b}	387.29±3.01^{B}	3.62±0.03	317.29±2.19^{b}	2.96±0.01
			GG (127)	10555±87.94^{ab}	376.39±3.77^{Aa}	3.59±0.04	310.49±2.75^{a}	2.96±0.01
NGFR	19:g.37113872C>G	1	CC (614)	10285±60.67	339.99±2.71	3.33±0.03	303.92±1.97	2.96±0.01
			CG (309)	10183±68.17	337.13±2.98	3.33±0.03	300.41±2.17	2.96±0.01
			GG (24)	10178±164.25	325.47±6.73	3.23±0.07	299.16±4.90	2.95±0.02
		2	CC (433)	10667±63.99	385.59±2.87^{Aa}	3.63±0.03^{a}	315.07±2.09	2.97±0.01
			CG (208)	10604±76.35	374.11±3.34^{Bb}	3.56±0.03^{b}	311.76±2.43	2.96±0.01
			GG (14)	10858±218.83	382.89±8.93^{ab}	3.55±0.09^{ab}	324.12±6.51	2.99±0.03
	19:g.37113157C>T	1	CC (245)	10308±71.99	336.8±3.12	3.29±0.03	303.58±2.28	2.96±0.01
			CT (496)	10213±61.73	339.45±2.75	3.35±0.03	301.89±2.00	2.96±0.01
			TT (206)	10277±75.16	339.92±3.26	3.33±0.03	303.72±2.37	2.96±0.01
		2	CC (165)	10685±80.37	375.57±3.48^{Aa}	3.55±0.03^{Aa}	314.73±2.54	2.96±0.01
			CT (336)	10578±67.63	383.18±3.00^{ABb}	3.64±0.03^{Bb}	312.34±2.18	2.96±0.01
			TT (154)	10762±84.80	387.39±3.66^{Bb}	3.60±0.03^{ab}	317.35±2.66	2.96±0.01
	19:g.37112276C>T	1	CC (613)	10282±60.67	340±2.71	3.33±0.03	303.85±1.98	2.96±0.01
			CT (307)	10185±68.24	337.15±2.98	3.33±0.03	300.48±2.17	2.96±0.01
			TT (27)	10265±156.32	328.68±6.41	3.23±0.06	301.75±4.68	2.95±0.02
		2	CC (432)	10668±63.97	385.51±2.87^{Aa}	3.63±0.03^{a}	315.07±2.09^{ab}	2.97±0.01
			CT (207)	10591±76.47	374.4±3.34^{Bb}	3.56±0.03^{b}	311.46±2.43^{a}	2.96±0.01
			TT (16)	11029±204.94	377.31±8.37^{ab}	3.44±0.08^{ab}	327.21±6.10^{b}	2.97±0.03
	19:g.37096050G>A	1	AA (151)	10250±82.34	334.61±3.52	3.30±0.03	301.03±2.57	2.95±0.01
			AG (461)	10243±62.86	339.51±2.79	3.34±0.03	302.59±2.03	2.96±0.01
			GG (335)	10258±66.84	339.56±2.94	3.33±0.03	303.41±2.14	2.97±0.01
		2	AA (95)	10689±97.49	374.92±4.15^{Aa}	3.53±0.04^{a}	316.07±3.02	2.97±0.01
			AG (315)	10592±68.81	379.12±3.04^{Aa}	3.61±0.03^{ab}	312.66±2.22	2.97±0.01
			GG (245)	10704±72.61	387.97±3.19^{B}	3.63±0.03^{b}	315.17±2.32	2.96±0.01
	19:g.37095131C>T	1	CC (333)	10255±66.94	339.48±2.94	3.33±0.03	303.34±2.14	2.97±0.01
			CT (461)	10253±62.89	339.72±2.79	3.34±0.03	302.84±2.03	2.96±0.01
			TT (153)	10225±82.07	334.15±3.51	3.30±0.03	300.37±2.56	2.95±0.01
		2	CC (242)	10707±72.72	388.28±3.19^{A}	3.63±0.03^{a}	315.23±2.32	2.96±0.01
			CT (317)	10590±68.97	378.86±3.05^{Bb}	3.61±0.03^{ab}	312.54±2.22	2.97±0.01
			TT (96)	10684±97.04	374.97±4.13^{Bb}	3.54±0.04^{b}	316.17±3.01	2.97±0.01

	19:g.37093264T>C	1	CC (161)	10243±81.05	333.94±3.47	3.29±0.03	300.92±2.53	2.95±0.01
			CT (476)	10239±62.33	340.11±2.77	3.34±0.03	302.73±2.01	2.97±0.01
			TT (310)	10269±68.26	339.03±2.99	3.32±0.03	303.37±2.18	2.96±0.01
		2	CC (101)	10701±95.98	376.23±4.09	3.54±0.04	317.02±2.98	2.98±0.01
			CT (328)	10630±68.32	381.11±3.03	3.61±0.03	313.84±2.20	2.97±0.01
			TT (226)	10652±74.66	385.67±3.26	3.63±0.03	313.25±2.38	2.95±0.01
	19:g.37091691C>A	1	AA (151)	10237±82.45	334.1±3.53	3.29±0.03	300.63±2.57	2.95±0.01
			AC (460)	10250±62.85	339.77±2.79	3.34±0.03	302.82±2.03	2.96±0.01
			CC (336)	10253±66.84	339.39±2.94	3.33±0.03	303.24±2.14	2.96±0.01
		2	AA (96)	10687±97.03	375.09±4.13^{Aa}	3.54±0.04^{a}	316.25±3.01	2.97±0.01
			AC(313)	10607±68.91	379.35±3.05^{Aa}	3.61±0.03^{ab}	312.98±2.22	2.97±0.01
			CC (246)	10685±72.56	387.66±3.18^{B}	3.64±0.03^{b}	314.69±2.32	2.96±0.01
ARNTL	15:g.39301344T>C	1	CC (110)	9993.38±89.73^{Aa}	334.41±3.81	3.36±0.04	295.07±2.78^{Aa}	2.96±0.01
			CT (472)	10201±62.34^{Ab}	337.34±2.77	3.33±0.03	301.48±2.02^{Ab}	2.96±0.01
			TT (365)	10384±65.40^{B}	342.07±2.88	3.32±0.03	306.29±2.10^{B}	2.96±0.01
		2	CC (77)	10578±104.16^{ab}	372.76±4.39^{Aa}	3.55±0.04	311.3±3.20^{ab}	2.95±0.01
			CT (328)	10740±66.75^{Aa}	386.54±2.97^{B}	3.62±0.03	317.31±2.16^{Aa}	2.97±0.01
			TT (249)	10546±71.85^{Bb}	378.66±3.15^{Aa}	3.62±0.03	310.64±2.30^{Bb}	2.96±0.02
	15:g.39312186T>C	1	CC (39)	10183±131.41^{ab}	334.84±5.43	3.31±0.05	298.2±3.96	2.94±0.02
			CT (336)	10155±66.76^{a}	337.25±2.94	3.35±0.03	301.01±2.14	2.97±0.01
			TT (572)	10304±60.67^{b}	339.89±2.70	3.32±0.03	303.75±1.97	2.96±0.01
		2	CC (26)	10817±165.22^{ab}	387.55±6.78^{ab}	3.61±0.07	318.6±4.94^{ab}	2.95±0.02
			CT (235)	10835±73.84^{Aa}	388.41±3.24^{Aa}	3.60±0.03	319.3±2.36^{Aa}	2.96±0.01
			TT (393)	10540±64.74^{Bb}	378.36±2.89^{Bb}	3.61±0.03	311.18±2.11^{Bb}	2.97±0.01
	15:g.39320936A>G	1	AA (693)	10291±60.13^{Aa}	339.85±2.69	3.33±0.02	304.28±1.96^{Aa}	2.97±0.01^{a}
			AG (230)	10195±71.28^{Aa}	337.34±3.10	3.32±0.03	299.91±2.25^{ABb}	2.95±0.01^{b}
			GG (24)	9613.54±159.18^{B}	327.76±6.52	3.41±0.06	288.2±4.75^{Bc}	3.00±0.02^{ab}
		2	AA (476)	10689±63.70^{Aa}	384.77±2.86^{a}	3.62±0.03	315.31±2.08	2.96±0.01^{Aa}
			AG (161)	10603±79.48^{ABa}	376.53±3.44^{b}	3.57±0.03	311.76±2.51	2.95±0.01^{Aa}
			GG (17)	10092±200.37^{Bb}	366.7±8.19^{ab}	3.65±0.08	307.59±5.97	3.06±0.03^{B}
PPP2R2B	7:g.58088217C>T	1	CC (738)	10268±59.20	340.45±2.66^{a}	3.34±0.02	303.59±1.93^{a}	2.96±0.01
			CT (200)	10166±77.26	332.89±3.34^{b}	3.31±0.03	298.81±2.43^{b}	2.95±0.01

		TT (9)	10332±264.07	329.58±10.69^{ab}	3.20±0.11	303.39±7.80^{ab}	2.94±0.04
	2	CC (524)	10655±61.84	382.42±2.79	3.61±0.03	314.06±2.03	2.96±0.01
		CT (126)	10626±90.40	380.11±3.87	3.62±0.04	314.69±2.82	2.98±0.01
		TT (5)	10563±359.05	379.28±14.53	3.64±0.14	309.22±10.60	2.93±0.05
7:g.57855248C>T	1	CC (759)	10275±58.99^{a}	340.35±2.65^{Aa}	3.33±0.02	303.85±1.93^{Aa}	2.97±0.01
		CT (182)	10103±79.88^{b}	331.57±3.44^{Bb}	3.31±0.03	296.65±2.50^{Bb}	2.95±0.01
		TT (6)	10488±336.67^{ab}	336.43±13.62^{ab}	3.21±0.1	304.35±9.94^{ab}	2.91±0.05
	2	CC (540)	10645±61.47	381.66±2.78	3.60±0.03	313.81±2.02	2.96±0.01
		CT (112)	10669±95.33	385.7±4.07	3.66±0.04	316.23±2.96	2.98±0.01
		TT (3)	10742±481.97	352.4±19.49	3.27±0.19	309.78±14.21	2.88±0.07
7:g.57855119T>C	1	CC (561)	10277±60.35	341.39±2.70^{Aa}	3.35±0.02	304.29±1.96^{Aa}	2.97±0.01^{Aa}
		CT (341)	10191±67.71	333.96±2.97^{Bb}	3.30±0.03	299.29±2.16^{Bb}	2.94±0.01^{Bb}
		TT (45)	10259±124.85	334.59±5.18^{ab}	3.29±0.05	303.16±3.78^{ab}	2.96±0.02^{ab}
	2	CC (389)	10621±64.32	379.5±2.88^{a}	3.59±0.03	312.58±2.10	2.96±0.01
		CT (230)	10695±75.06	386.97±3.29^{b}	3.65±0.03	317.02±2.39	2.98±0.01
		TT (36)	10756±142.26	383.81±5.90^{ab}	3.59±0.06	316.7±4.30	2.94±0.02
7:g.57794491G>T	1	GG (664)	10275±60.89	339.99±2.72	3.33±0.03	303.77±1.98	2.96±0.01
		GT (254)	10169±69.92	334.86±3.05	3.32±0.03	299.95±2.22	2.96±0.01
		TT (29)	10409±151.37	348.45±6.22	3.38±0.06	304.89±4.53	2.94±0.02
	2	GG (463)	10688±65.00	382±2.92^{Aa}	3.60±0.03	314.97±2.12	2.96±0.01
		GT (171)	10529±78.90	377.92±3.42^{Aa}	3.60±0.03	311.38±2.49	2.97±0.01
		TT (21)	10840±182.65	404.88±7.48^{B}	3.75±0.07	318.85±5.46	2.95±0.03

Note: The number in the table represents the mean ± standard deviation; the number in the bracket represents the number of cows for the corresponding genotype; P value shows the significance for the genetic effects of SNPs; a, b, c within the same column with different superscripts means $P < 0.05$; and A, B, C within the same column with different superscripts means $P < 0.01$.
