# Supplementary material for: Associations between polymorphisms of SLC22A7, NGFR, ARNTL and PPP2R2B genes and Milk production traits in Chinese Holstein
Source: BMC Genom Data. 2021 Nov 3;22:47. doi: 10.1186/s12863-021-01002-0 (PMC8567656; doi:10.1186/s12863-021-01002-0)
Supplement: Supplementary file 2 — Additional file 2: Table S2. Additive, dominant and allele substitution effects of 20 SNPs in SLC22A7, NGFR, ARNTL and PPP2R2B genes on milk yield and composition traits in Chinese Holstein cattle during two lactations. [file 12863_2021_1002_MOESM2_ESM.pdf]

Additional file 2: Table S2 Additive, dominant and allele substitution effects of 20 SNPs in *SLC22A7*, *NGFR*, *ARNTL* and *PPP2R2B* genes on milk yield and composition traits in Chinese Holstein cattle during two lactations.

Table S2-1 Additive, dominant and allele substitution effects of SNPs in *SLC22A7*.

SNPs	Lactation	Effect	Milk yield (kg)	Fat yield (kg)	Fat percentage (%)	Protein yield (kg)	Protein percentage (%)
23:g.16896145A>G	1	Additive effect(a)	-224.68**	-2.90	0.05	-5.83*	0.01
		Dominant effect(d)	308.61**	5.09	-0.06	6.56*	-0.03*
		Substitution effect(α)	-431.94**	-6.32	0.08	-10.23**	0.03
	2	Additive effect(a)	-158.08	-4.01	0.03	-3.46	0.01
		Dominant effect(d)	359.02**	10.88**	-0.05	8.84**	-0.02
		Substitution effect(α)	-397.97**	-11.28	0.06	-9.37*	0.02
23:g.16899640A>G	1	Additive effect(a)	60.16	-1.21	-0.02	1.20	-0.002
		Dominant effect(d)	-104.18	-6.44**	-0.02	-3.75*	-0.01
		Substitution effect(α)	12.74	-4.14	-0.03	-0.51	-0.005
	2	Additive effect(a)	-7.27	-2.82	-0.02	1.33	0.01
		Dominant effect(d)	-50.94	-4.91	-0.02	0.03	0.02
		Substitution effect(α)	-30.95	-5.10	-0.03	1.34	0.02
23:g.16900723A>T	1	Additive effect(a)	60.16	-1.21	-0.02	1.20	-0.002
		Dominant effect(d)	-104.18	-6.44**	-0.02	-3.75*	-0.01
		Substitution effect(α)	12.74	-4.14	-0.03	-0.51	-0.005
	2	Additive effect(a)	-7.27	-2.82	-0.02	1.33	0.01
		Dominant effect(d)	-50.94	-4.91	-0.02	0.03	0.02
		Substitution effect(α)	-30.95	-5.10	-0.03	1.34	0.02

23:g.16900870G>T	1	Additive effect(a)	-117.56**	-2.74	0.01	-3.21**	0.0008
		Dominant effect(d)	28.93	-1.11	-0.02	-0.03	-0.01
		Substitution effect(α)	-115.15**	-2.84	0.01	-3.21**	0.00008
	2	Additive effect(a)	63.26	2.36	-0.0016	1.96	-0.0003
		Dominant effect(d)	221.42**	6.65**	-0.02	3.98*	-0.02**
		Substitution effect(α)	77.12	2.78	-0.003	2.21	-0.0017
23:g.16901383G>C	1	Additive effect(a)	108.95**	2.21	-0.01	3.58**	0.01
		Dominant effect(d)	-20.58	-0.49	-0.0015	-0.58	-0.001
		Substitution effect(α)	107**	2.17	-0.01	3.52**	0.01
	2	Additive effect(a)	-6.37	0.44	0.01	0.51	0.01
		Dominant effect(d)	205.58**	10.47**	0.02	6.29**	-0.002
		Substitution effect(α)	15.62	1.56	0.01	1.18	0.01

Note: The number in the table represents the mean; * indicates $P < 0.05$; ** indicates $P < 0.01$.

Table S2-2 Additive, dominant and allele substitution effects of SNPs in *NGFR*.

SNPs	Lactation	Effect	Milk yield (kg)	Fat yield (kg)	Fat percentage (%)	Protein yield (kg)	Protein percentage (%)
19:g.37113872C>G	1	Additive effect(a)	53.33	7.26*	0.05	2.38	0.01
		Dominant effect(d)	-48.69	4.40	0.05	-1.13	0.005
		Substitution effect(α)	23.00	10.00*	0.09	1.68	0.01
	2	Additive effect(a)	-95.24	1.35	0.04	-4.53	-0.01
		Dominant effect(d)	-157.95	-10.13*	-0.04	-7.83*	-0.02
		Substitution effect(α)	-195.95	-5.11	0.02	-9.52	-0.03
19:g.37113157C>T	1	Additive effect(a)	15.92	-1.56	-0.02	-0.07	-0.003
		Dominant effect(d)	-79.70	1.09	0.03	-1.76	0.01
		Substitution effect(α)	12.63	-1.52	-0.02	-0.15	-0.003
	2	Additive effect(a)	-38.60	-5.91**	-0.03	-1.31	-0.0005
		Dominant effect(d)	-145.96*	1.70	0.07**	-3.70*	0.002
		Substitution effect(α)	-41.28	-5.88**	-0.03	-1.38	-0.0005
19:g.37112276C>T	1	Additive effect(a)	8.69	5.66	0.05	1.05	0.01
		Dominant effect(d)	-88.82	2.81	0.05	-2.32	0.01
		Substitution effect(α)	-46.27	7.40	0.08	-0.39	0.01
	2	Additive effect(a)	-180.45	4.10	0.10*	-6.07*	-0.004
		Dominant effect(d)	-257.08*	-7.01	0.03	-9.67**	-0.01
		Substitution effect(α)	-343.6*	-0.35	0.11	-12.21*	-0.01
19:g.37096050G>A	1	Additive effect(a)	-4.34	-2.47	-0.02	-1.19	-0.007
		Dominant effect(d)	-10.56	2.42	0.02	0.37	0.005

		Substitution effect(α)	-2.29	-2.94	-0.02	-1.26	-0.01
	2	Additive effect(a)	-7.24	-6.53**	-0.05*	0.45	0.01
		Dominant effect(d)	-104.6	-2.32	0.03	-2.96	0.003
		Substitution effect(α)	16.42	-6.00**	-0.06*	1.12	0.01
19:g.37095131C>T	1	Additive effect(a)	14.80	2.66	0.02	1.48	0.01
		Dominant effect(d)	12.80	2.90	0.02	0.98	0.004
		Substitution effect(α)	17.24	3.21	0.02	1.67	0.01
	2	Additive effect(a)	11.18	6.65**	0.05*	-0.47	-0.01
		Dominant effect(d)	-105.73	-2.76	0.02	-3.15	0.002
		Substitution effect(α)	-12.28	6.04**	0.05*	-1.17	-0.01
19:g.37093264T>C	1	Additive effect(a)	-13.17	-2.54	-0.02	-1.22	-0.01
		Dominant effect(d)	-16.81	3.62	0.04	0.59	0.01
		Substitution effect(α)	-10.52	-3.11	-0.02	-1.32	-0.01
	2	Additive effect(a)	24.35	-4.72*	-0.04*	1.89	0.01
		Dominant effect(d)	-46.27	0.16	0.03	-1.30	0.0004
		Substitution effect(α)	33.04	-4.75*	-0.05*	2.13	0.01
19:g.37091691C>A	1	Additive effect(a)	-7.92	-2.65	-0.02	-1.30	-0.01
		Dominant effect(d)	4.91	3.02	0.02	0.88	0.01
		Substitution effect(α)	-8.88	-3.24	-0.02	-1.48	-0.01
	2	Additive effect(a)	1.18	-6.28**	-0.05*	0.78	0.01
		Dominant effect(d)	-78.66	-2.03	0.02	-2.49	-0.0002
		Substitution effect(α)	18.97	-5.83*	-0.06*	1.34	0.01

Note: The number in the table represents the mean; * indicates $P < 0.05$; ** indicates $P < 0.01$.

Table S2-3 Additive, dominant and allele substitution effects of SNPs in *ARNTL*.

SNPs	Lactation	Effect	Milk yield (kg)	Fat yield (kg)	Fat percentage (%)	Protein yield (kg)	Protein percentage (%)
15:g.39301344T>C	1	Additive effect(a)	-195.29**	-3.83*	0.02	-5.61**	-0.002
		Dominant effect(d)	12.41	-0.90	-0.01	0.80	0.01
		Substitution effect(α)	-198.63**	-3.58	0.02	-5.82**	-0.003
	2	Additive effect(a)	15.76	-2.95	-0.03	0.33	-0.01
		Dominant effect(d)	178.47**	10.83**	0.03	6.34**	0.01
		Substitution effect(α)	-31.18	-5.80*	-0.04	-1.34	-0.01
15:g.39312186T>C	1	Additive effect(a)	-60.39	-2.52	-0.01	-2.78	-0.01
		Dominant effect(d)	-89.33	-0.12	0.03	0.03	0.03*
		Substitution effect(α)	-10.11	-2.46	-0.02	-2.80	-0.03
	2	Additive effect(a)	138.48	4.60	-0.002	3.71	-0.01
		Dominant effect(d)	156	5.46	-0.01	4.41	-0.0008
		Substitution effect(α)	50.93	1.54	0.003	1.24	-0.01
15:g.39320936A>G	1	Additive effect(a)	338.95**	6.05*	-0.04	8.04**	-0.02
		Dominant effect(d)	242.46**	3.53	-0.05	3.67	-0.04**
		Substitution effect(α)	510.23**	8.54	-0.07	10.63**	-0.04*
	2	Additive effect(a)	298.81**	9.03*	-0.01	3.86	-0.05**
		Dominant effect(d)	212.23	0.80	-0.06	0.31	-0.06**
		Substitution effect(α)	447.75**	9.59	-0.06	4.08	-0.09**

Note: The number in the table represents the mean; * indicates $P < 0.05$; ** indicates $P < 0.01$.

Table S2-4 Additive, dominant and allele substitution effects of SNPs in *PPP2R2B*.

SNPs	Lactation	Effect	Milk yield (kg)	Fat yield (kg)	Fat percentage (%)	Protein yield (kg)	Protein percentage (%)
7:g.58088217C>T	1	Additive effect(a)	-31.77	5.43	0.07	0.10	0.01
		Dominant effect(d)	-134.06	-2.13	0.04	-4.68	-0.001
		Substitution effect(α)	-134.97	3.79	0.09	-3.51	0.01
	2	Additive effect(a)	46.03	1.57	-0.02	2.42	0.02
		Dominant effect(d)	17.02	-0.74	-0.005	3.05	0.03
		Substitution effect(α)	59.51	0.98	-0.02	4.83	0.04
7:g.57855248C>T	1	Additive effect(a)	-106.48	1.96	0.06	-0.25	0.03
		Dominant effect(d)	-278.83	-6.82	0.04	-7.45	0.01
		Substitution effect(α)	-328.21	-3.46	0.09	-6.17	0.04
	2	Additive effect(a)	-48.30	14.63	0.17	2.02	0.04
		Dominant effect(d)	-25.06	18.67	0.23*	4.44	0.06
		Substitution effect(α)	-68.85	29.93	0.35*	5.65	0.09
7:g.57855119T>C	1	Additive effect(a)	9.22	3.40	0.03	0.56	0.004
		Dominant effect(d)	-76.81	-4.03	-0.02	-4.43*	-0.02*
		Substitution effect(α)	-32.63	1.20	0.02	-1.85	-0.01
	2	Additive effect(a)	-67.86	-2.15	-0.001	-2.06	0.01

		Dominant effect(d)	7.02	5.31	0.05	2.38	0.02
		Substitution effect(α)	-64.10	0.70	0.03	-0.79	0.02
7:g.57794491G>T	1	Additive effect(a)	-67.02	-4.23	-0.02	-0.56	0.01
		Dominant effect(d)	-172.9*	-9.36**	-0.04	-4.37	0.01
		Substitution effect(α)	-182.97	-10.50*	-0.05	-3.49	0.02
	2	Additive effect(a)	-75.64	-11.44**	-0.08*	-1.94	0.004
		Dominant effect(d)	-235.19*	-15.52**	-0.07	-5.53	0.02
		Substitution effect(α)	-233.87	-21.88**	-0.12*	-5.66	0.02

Note: The number in the table represents the mean; * indicates $P < 0.05$; ** indicates $P < 0.01$.
