# Supplementary material for: Associations between polymorphisms of SLC22A7, NGFR, ARNTL and PPP2R2B genes and Milk production traits in Chinese Holstein
Source: BMC Genom Data. 2021 Nov 3;22:47. doi: 10.1186/s12863-021-01002-0 (PMC8567656; doi:10.1186/s12863-021-01002-0)
Supplement: Supplementary file 3 — Additional file 3: Table S3. Haplotypes analyses for SLC22A7, NGFR and ARNTL genes. [file 12863_2021_1002_MOESM3_ESM.pdf]

Additional file 3: Table S3 Haplotypes analyses for *SLC22A7*, *NGFR* and *ARNTL* genes.

Table S3-1 Haplotypes analyses for *SLC22A7*.

Lactation	Haplotype combination	Milk yield (kg)	Fat yield (kg)	Fat percentage (%)	Protein yield (kg)	Protein percentage (%)
1	H1H1 (202)	10540±76.42^{a}	347.46±3.32^{Aa}	3.31±0.03	311.76±2.42^{Aa}	2.95±0.01
	H1H2 (240)	10305±71.21^{b}	338.36±3.12^{ABbc}	3.29±0.03	304.06±2.27^{BCbc}	2.95±0.01
	H1H3 (138)	10553±81.74^{a}	346.73±3.50^{ABab}	3.29±0.03	310.9±2.56^{ABCab}	2.94±0.01
	H1H4 (69)	10612±104.65^{a}	345.36±4.38^{ABabc}	3.27±0.04	315.19±3.19^{Aa}	2.97±0.01
	H2H2 (64)	10335±107.68^{ab}	344.53±4.50^{ABabc}	3.33±0.04	305.74±3.28^{ABCabc}	2.95±0.02
	H2H3 (87)	10322±95.64^{ab}	333.67±4.02^{Bc}	3.24±0.04	301.91±2.93^{Cc}	2.92±0.01
2	H1H1 (146)	10768±86.10^{Aa}	389.09±3.72^{a}	3.62±0.04	321.63±2.71	2.99±0.01
	H1H2 (170)	10872±80.35^{ab}	389.29±3.50^{a}	3.58±0.03	322.64±2.55	2.97±0.01
	H1H3 (99)	11159±95.79^{Bb}	401.99±4.08^{b}	3.58±0.04	329.9±2.97	2.95±0.01
	H1H4 (43)	10748±131.73^{ab}	383.44±5.47^{a}	3.58±0.05	320.54±3.99	2.99±0.02
	H2H2 (40)	10891±135.58^{ab}	390.81±5.64^{ab}	3.59±0.05	321.55±4.11	2.95±0.02
	H2H3 (57)	10931±119.65^{ab}	388.07±5.01^{ab}	3.55±0.05	322.63±3.65	2.95±0.02

Note: The number in the table represents the mean ± standard deviation; H means haplotype; H1: GAATC, H2: GGTGG, H3: AAAGG, H4: GAAGC; the number in the bracket represents the number of cows for the Haplotype combination; P value shows the significance for the genetic effects of Haplotype combination; a, b, c, d, e within the same column with different superscripts means $P < 0.05$; and A, B, C, D, E within the same column with different superscripts means $P < 0.01$.

Table S3-2 Haplotypes analyses for *NGFR*.

Lactation	Haplotype combination	Milk yield (kg)	Fat yield (kg)	Fat percentage (%)	Protein yield (kg)	Protein percentage (%)
1	H1H1 (204)	10450±75.53^{ab}	345.11±3.28^{ab}	3.31±0.03	309.75±2.39^{ab}	2.96±0.01
	H1H2 (185)	10546±77.88^{a}	351.37±3.37^{a}	3.35±0.03	312.46±2.46^{Aa}	2.96±0.01
	H1H3 (189)	10284±76.93^{b}	340.7±3.31^{b}	3.32±0.03	303.2±2.41^{Bb}	2.95±0.01
	H1H4 (93)	10461±95.03^{ab}	343.94±4.02^{ab}	3.30±0.04	309.04±2.93^{ab}	2.96±0.01
	H2H3 (86)	10342±97.81^{ab}	342.96±4.12^{ab}	3.33±0.04	305.64±2.00^{ab}	2.96±0.01
2	H1H1 (152)	10873±84.68	393.97±3.67^{ab}	3.62±0.03^{ab}	322.03±2.67^{ab}	2.97±0.01
	H1H2 (120)	10915±90.98	395.66±3.91^{ab}	3.63±0.04^{ab}	323.92±2.85^{a}	2.97±0.01
	H1H3 (131)	10666±91.11	385.69±3.91^{Aa}	3.64±0.04^{ab}	314.75±2.85^{b}	2.95±0.01
	H1H4 (63)	10694±115.43	403.7±4.84^{Bb}	3.76±0.05^{Aa}	314.62±3.53^{ab}	2.94±0.02
	H2H3 (56)	10931±118.28	386.04±4.94^{Aab}	3.53±0.05^{Bb}	321±3.61^{ab}	2.94±0.02

Note: The number in the table represents the mean ± standard deviation; H means haplotype; H1: CTCGCTC, H2: ACTACCC, H3: ACTATCG, H4: CTCGCCC; the number in the bracket represents the number of cows for the Haplotype combination; P value shows the significance for the genetic effects of Haplotype combination; a, b, c, d, e within the same column with different superscripts means $P < 0.05$; and A, B, C, D, E within the same column with different superscripts means $P < 0.01$.

Table S3-3 Haplotypes analyses for *ARNTL*.

Lactation	Haplotype combination	Milk yield (kg)	Fat yield (kg)	Fat percentage (%)	Protein yield (kg)	Protein percentage (%)
1	H1H1 (365)	10490±66.10^{A}	348.86±2.92^{Aa}	3.34±0.03	310.14±2.12^{Aa}	2.96±0.01^{ab}
	H1H2 (289)	10275±68.07^{Bb}	342.95±2.98^{ab}	3.35±0.03	305.23±2.17^{ABb}	2.98±0.01^{Aa}
	H1H3 (183)	10260±76.05^{Bb}	339.59±3.28^{Bb}	3.32±0.03	301.38±2.39^{Bbc}	2.94±0.01^{Bb}
	H2H3 (47)	10040±121.47^{Bb}	339.13±5.04^{ab}	3.38±0.05	295.78±3.67^{Bc}	2.95±0.02^{ab}
2	H1H1 (249)	10773±73.34^{Aa}	386.92±3.23^{Aa}	3.61±0.03	319.98±2.35^{Aa}	2.97±0.01^{Aa}
	H1H2 (201)	11073±77.05^{Bb}	400.83±3.37^{B}	3.62±0.03	328.44±2.45^{B}	2.96±0.01^{ABa}
	H1H3 (127)	10745±84.89^{Aa}	382.59±3.64^{Aa}	3.57±0.03	317.49±2.65^{Aa}	2.96±0.01^{ABa}
	H2H3 (34)	10791±143.47^{ab}	376.45±5.93^{Aa}	3.49±0.06	313.83±4.32^{Aa}	2.90±0.02^{Bb}

Note:The number in the table represents the mean ± standard deviation; H means haplotype; H1: TTA, H2: CCA, H3: CTG; the number in the bracket represents the number of cows for the Haplotype combination; P value shows the significance for the genetic effects of Haplotype combination; a, b, c, d, e within the same column with different superscripts means $P < 0.05$; and A, B, C, D, E within the same column with different superscripts means $P < 0.01$.

Additional file 4: Table S4 The phenotypic values for milk yield and composition in two lactations and pedigree information.

Table S4-1 The estimated coefficient for the 305-day milk yield calculation.

Days in milk	First lactation	Second lactation	Days in milk	First lactation	Second lactation
30	8.32	7.42	180	1.51	1.41
40	6.24	5.57	190	1.44	1.35
50	4.99	4.47	200	1.33	1.3
60	4.16	3.74	210	1.32	1.26
70	3.58	3.23	220	1.27	1.22
80	3.15	2.85	230	1.23	1.18
90	2.82	2.56	240	1.19	1.14
100	2.55	2.32	250	1.15	1.11
110	2.34	2.13	260	1.12	1.09
120	2.16	1.98	270	1.08	1.06
130	2.01	1.85	280	1.06	1.04
140	1.88	1.73	290	1.03	1.03
150	1.77	1.64	300	1.01	1.01
160	1.61	1.55	>305	actual total milk yield	
170	1.58	1.48			
