# Supplementary material for: Associations between polymorphisms of SLC22A7, NGFR, ARNTL and PPP2R2B genes and Milk production traits in Chinese Holstein
Source: BMC Genom Data. 2021 Nov 3;22:47. doi: 10.1186/s12863-021-01002-0 (PMC8567656; doi:10.1186/s12863-021-01002-0)
Supplement: Supplementary file 4 — Additional file 4: Table S4. The phenotypic values for milk yield and composition in two lactations and pedigree information. [file 12863_2021_1002_MOESM4_ESM.pdf]

Table S3-3 Haplotypes analyses for *ARNTL*.

Lactation	Haplotype combination	Milk yield (kg)	Fat yield (kg)	Fat percentage (%)	Protein yield (kg)	Protein percentage (%)
1	H1H1 (365)	$10490±66.10^{A}$	$348.86±2.92^{Aa}$	3.34±0.03	$310.14±2.12^{Aa}$	$2.96±0.01^{ab}$
	H1H2 (289)	$10275±68.07^{Bb}$	$342.95±2.98^{ab}$	3.35±0.03	$305.23±2.17^{ABb}$	$2.98±0.01^{Aa}$
	H1H3 (183)	$10260±76.05^{Bb}$	$339.59±3.28^{Bb}$	3.32±0.03	$301.38±2.39^{Bbc}$	$2.94±0.01^{Bb}$
	H2H3 (47)	$10040±121.47^{Bb}$	$339.13±5.04^{ab}$	3.38±0.05	$295.78±3.67^{Bc}$	$2.95±0.02^{ab}$
2	H1H1 (249)	$10773±73.34^{Aa}$	$386.92±3.23^{Aa}$	3.61±0.03	$319.98±2.35^{Aa}$	$2.97±0.01^{Aa}$
	H1H2 (201)	$11073±77.05^{Bb}$	$400.83±3.37^{B}$	3.62±0.03	$328.44±2.45^{B}$	$2.96±0.01^{ABa}$
	H1H3 (127)	$10745±84.89^{Aa}$	$382.59±3.64^{Aa}$	3.57±0.03	$317.49±2.65^{Aa}$	$2.96±0.01^{ABa}$
	H2H3 (34)	$10791±143.47^{ab}$	$376.45±5.93^{Aa}$	3.49±0.06	$313.83±4.32^{Aa}$	$2.90±0.02^{Bb}$

Note:The number in the table represents the mean ± standard deviation; H means haplotype; H1: TTA, H2: CCA, H3: CTG; the number in the bracket represents the number of cows for the Haplotype combination; P value shows the significance for the genetic effects of Haplotype combination; a, b, c, d, e within the same column with different superscripts means $P < 0.05$; and A, B, C, D, E within the same column with different superscripts means $P < 0.01$.

Additional file 4: Table S4 The phenotypic values for milk yield and composition in two lactations and pedigree information.

Table S4-1 The estimated coefficient for the 305-day milk yield calculation.

Days in milk	First lactation	Second lactation	Days in milk	First lactation	Second lactation
30	8.32	7.42	180	1.51	1.41
40	6.24	5.57	190	1.44	1.35
50	4.99	4.47	200	1.33	1.3
60	4.16	3.74	210	1.32	1.26
70	3.58	3.23	220	1.27	1.22
80	3.15	2.85	230	1.23	1.18
90	2.82	2.56	240	1.19	1.14
100	2.55	2.32	250	1.15	1.11
110	2.34	2.13	260	1.12	1.09
120	2.16	1.98	270	1.08	1.06
130	2.01	1.85	280	1.06	1.04
140	1.88	1.73	290	1.03	1.03
150	1.77	1.64	300	1.01	1.01
160	1.61	1.55	>305	actual total milk yield	
170	1.58	1.48			

Table S4-2 Descriptive statistics of phenotypic values for dairy production traits of the two lactations.

Traits	Lactation	Mean	SD	Min	Max	CV
Milk yield (kg)	1	10420.11	1483.71	6057.96	14505.68	0.14
	2	10907.51	1916.08	4639.17	16512.08	0.18
Fat yield (kg)	1	352.62	61.54	184.80	537.97	0.17
	2	393.28	87.05	104.05	741.92	0.22
Fat percentage (%)	1	3.39	0.43	2.06	4.74	0.13
	2	3.61	0.52	2.21	5.43	0.14
Protein yield (kg)	1	315.41	48.52	157.73	457.53	0.15
	2	324.14	59.01	108.06	467.03	0.18
Protein percentage (%)	1	3.03	0.20	2.24	3.51	0.07
	2	2.97	0.19	2.20	3.54	0.07

Note: SD: standard deviation; Min: minimum; Max: maximum; CV: coefficient of variation.

Table S4-3 Records of phenotypic values for dairy production traits of the first lactation.

ID	Milk yield (kg)	Fat yield (kg)	Fat percentage (%)	Protein yield (kg)	Protein percentage (%)
110046120121	11428.69	387.7181	3.3925	334.3462	2.9255
110046120018	7749.414	218.3088	2.8171	246.6096	3.1823
110046011145	11582.9	321.2284	2.7733	379.3051	3.2747
110046120099	10025.67	404.4955	4.0346	309.3218	3.0853
110046011040	10649.24	332.2242	3.1197	346.8669	3.2572
110046011147	13055.32	333.7984	2.5568	409.2059	3.1344
110046120034	7246.431	236.8931	3.2691	228.335	3.151
110046011262	11257.12	262.3133	2.3302	334.1787	2.9686
110046120028	11892.57	324.8218	2.7313	369.1454	3.104
110046011253	14349.65	392.6782	2.7365	451.4974	3.1464
110046011155	10446.15	277.8573	2.6599	334.0994	3.1983
110046120051	11911.43	346.587	2.9097	357.2715	2.9994
110046120024	11352.88	338.963	2.9857	348.6925	3.0714
110046011179	9803.675	398.0684	4.0604	319.5606	3.2596
110046011068	8058.141	297.7483	3.695	232.1873	2.8814
110046011267	10690.11	289.6272	2.7093	343.0564	3.2091
110046011279	14070.55	321.5965	2.2856	403.5716	2.8682
110046011158	12226.14	356.4285	2.9153	391.6398	3.2033
110046120001	8880.151	229.1967	2.581	288.8269	3.2525
110046011259	8379.923	212.5986	2.537	264.3531	3.1546
110046011144	13260.45	390.6795	2.9462	419.574	3.1641
110046120006	13092.73	352.4038	2.6916	421.4287	3.2188
110046120023	13082.88	386.8476	2.9569	401.9322	3.0722
110046011178	11491.96	307.2951	2.674	355.9521	3.0974
110046011037	10639.05	310.4368	2.9179	332.832	3.1284

110046120126	13035.44	425.0336	3.2606	376.281	2.8866
110046011153	11987.12	328.639	2.7416	380.8549	3.1772
110046120103	11000.16	333.7229	3.0338	286.9392	2.6085
110046011189	11685	376.1752	3.2193	376.9815	3.2262
110046120027	8432.702	266.6842	3.1625	268.6659	3.186
110046011138	11499.84	333.0698	2.8963	334.6107	2.9097
110046011265	10015.13	295.8169	2.9537	351.3307	3.508
110046120089	10222.34	309.7981	3.0306	312.5582	3.0576
110046011263	9780.431	313.7953	3.2084	317.8933	3.2503
110046011148	10410.27	284.2109	2.7301	331.6193	3.1855
110046011198	12677.66	362.6953	2.8609	372.7487	2.9402
110045X11128	11887.36	400.9131	3.3726	372.8827	3.1368
110045010227	12691.79	381.6674	3.0072	370.5875	2.9199
110045011241	12339.58	406.6384	3.2954	353.8743	2.8678
110045011254	14222.98	400.9884	2.8193	399.7794	2.8108
110045010262	12710.14	392.9721	3.0918	387.6465	3.0499
110045120031	10854.59	283.8475	2.615	313.6867	2.8899
110045120048	10963.61	358.9158	3.2737	325.1589	2.9658
110045120030	10829.97	299.9576	2.7697	343.8947	3.1754
110045011118	11940.93	386.3846	3.2358	358.5861	3.003
110045120009	9615.884	334.5847	3.4795	313.2855	3.258
110045011270	11416.07	364.7664	3.1952	328.2235	2.8751
110045X11253	8760.024	290.4824	3.316	277.6139	3.1691
110045120023	10020.9	307.0805	3.0644	311.3995	3.1075
110045011252	9224.58	309.743	3.3578	290.0485	3.1443
110045011251	10743.93	356.7092	3.3201	347.3405	3.2329
110045011200	10089.26	328.1431	3.2524	313.9778	3.112
110045120036	8680.353	233.4581	2.6895	278.5004	3.2084
110045120053	10411.17	340.081	3.2665	323.3502	3.1058
110045120021	9253.346	360.0662	3.8912	297.7819	3.2181
110045011244	10917.88	337.1115	3.0877	302.6874	2.7724
110045120063	10888.15	378.2977	3.4744	349.0195	3.2055
110045011278	11365.66	381.693	3.3583	354.9042	3.1226
110045120099	10607.21	323.0639	3.0457	313.2098	2.9528
110045010278	10411.61	214.3335	2.0586	288.308	2.7691
110045011247	12366.78	349.9305	2.8296	360.5041	2.9151
110045011266	12249.5	425.5598	3.4741	361.4581	2.9508
110045120025	8080.682	254.994	3.1556	244.3113	3.0234
110045011284	9169.143	300.1335	3.2733	295.0905	3.2183
110045011193	11320.6	370.5797	3.2735	361.3082	3.1916
110024011202	10488.93	412.6451	3.9341	337.7017	3.2196
110024120036	9270.714	335.4886	3.6188	284.9354	3.0735
110024011337	9182.029	334.9513	3.6479	292.2273	3.1826
110024011336	11039.83	356.0123	3.2248	333.745	3.0231

110024011270	9778.051	326.1273	3.3353	297.1452	3.0389
110024011126	8189.991	321.8093	3.9293	260.581	3.1817
110024011313	6671.729	274.2347	4.1104	206.4366	3.0942
110024120030	11173.28	419.8138	3.7573	355.5116	3.1818
110024120004	9251.637	392.0289	4.2374	293.0271	3.1673
110024011297	10604.05	380.4522	3.5878	338.4071	3.1913
110024011258	10935.53	388.08	3.5488	351.0414	3.2101
110024120091	8027.035	290.2175	3.6155	230.7211	2.8743
110024011333	9127.209	341.1021	3.7372	272.0365	2.9805
110024011192	8404.319	297.0254	3.5342	253.3566	3.0146
110024011253	10760.68	389.0418	3.6154	315.4387	2.9314
110024011338	10783.65	367.6794	3.4096	326.1623	3.0246
110024120125	10658.63	349.8909	3.2827	302.6732	2.8397
110024011304	10412.02	372.8961	3.5814	304.0519	2.9202
110024011201	8180.23	335.5203	4.1016	245.9632	3.0068
110024011308	7867.972	331.7058	4.2159	241.5861	3.0705
110024120032	6681.833	251.9118	3.7701	216.8589	3.2455
110024011299	10505.75	318.7445	3.034	310.8967	2.9593
110024011315	7584.633	291.0375	3.8372	229.2607	3.0227
110024011275	7459.85	263.2805	3.5293	210.7557	2.8252
110024011300	9879.639	373.974	3.7853	291.3802	2.9493
110024120005	9785.147	315.4731	3.224	307.7037	3.1446
110024120008	10273.02	370.8151	3.6096	296.7979	2.8891
110024011343	9131.123	331.9072	3.6349	285.795	3.1299
110024011295	9609.195	333.6985	3.4727	293.2438	3.0517
110024011028	8858.58	341.4983	3.855	271.4623	3.0644
110024011327	10799.06	364.479	3.3751	304.8034	2.8225
110024011335	8845.058	335.7672	3.7961	267.0234	3.0189
110024010118	9793.098	307.4249	3.1392	295.5851	3.0183
110024011263	8697.527	334.3068	3.8437	286.4617	3.2936
110024011339	8763.894	343.6849	3.9216	272.5133	3.1095
110024011326	7893.947	265.8839	3.3682	230.9216	2.9253
110024011283	9733.33	368.2508	3.7834	310.172	3.1867
110024011204	11104.83	403.8049	3.6363	313.1007	2.8195
110024011322	10014.43	356.814	3.563	298.9306	2.985
110024120018	9884.9	367.2438	3.7152	310.1091	3.1372
110022511428	10737.56	317.1446	2.9536	314.0844	2.9251
110022511520	6168.708	190.3108	3.0851	185.1538	3.0015
110022110418	12670.79	414.7785	3.2735	396.4945	3.1292
110022511431	12288.23	400.3627	3.2581	363.1539	2.9553
110022512164	9884.664	287.9304	2.9129	291.6668	2.9507
110022511446	12372.91	379.5018	3.0672	376.5075	3.043
110022512003	11641.71	316.1306	2.7155	338.2731	2.9057
110022511533	10079.76	325.9896	3.2341	297.8166	2.9546

110022512014	11941.2	400.1496	3.351	350.4026	2.9344
110022512083	12630.33	383.1159	3.0333	366.6839	2.9032
110022512138	9626.21	302.2245	3.1396	279.988	2.9086
110022511408	10158.63	338.3025	3.3302	324.7001	3.1963
110022511461	10398.22	323.915	3.1151	304.5743	2.9291
110022512007	12187.73	321.2441	2.6358	370.5191	3.0401
110022110399	12021.82	299.4275	2.4907	294.0657	2.4461
110022512159	10643.51	330.7043	3.1071	306.5755	2.8804
110022512001	12264.65	383.0618	3.1233	350.1067	2.8546
110022512077	10272.67	327.4105	3.1872	286.289	2.7869
110022511424	8152.56	286.856	3.5186	256.6589	3.1482
110022512112	9999.298	316.7178	3.1674	287.7498	2.8777
110022511522	9413.12	283.0713	3.0072	274.223	2.9132
110022511556	11506.03	385.3716	3.3493	349.6223	3.0386
110022512118	10377.56	328.9687	3.17	308.9919	2.9775
110022511411	12298.3	392.4633	3.1912	363.8452	2.9585
110022512158	9255.435	327.6239	3.5398	271.5822	2.9343
110022511512	12229.17	395.687	3.2356	369.3331	3.0201
110022512163	10245.34	313.8557	3.0634	301.0285	2.9382
110022512010	10887.38	353.8181	3.2498	321.0253	2.9486
110022511555	11921.49	397.2957	3.3326	392.1933	3.2898
110022512023	8673.016	296.8253	3.4224	260.7629	3.0066
110022511405	10990.41	348.0002	3.1664	324.6346	2.9538
110022512144	10460.17	368.5642	3.5235	293.5752	2.8066
110022512177	10482.63	370.3515	3.533	305.6946	2.9162
110022511443	10385.99	302.5128	2.9127	323.5029	3.1148
110022511495	8097.628	266.3715	3.2895	248.7429	3.0718
110022511558	10268.36	313.4416	3.0525	307.7529	2.9971
110022511481	9927.995	346.5664	3.4908	305.9113	3.0813
110022511076	11303	384.7768	3.4042	367.2572	3.2492
110022511458	9963.807	331.2169	3.3242	314.119	3.1526
110022511539	10282.98	315.9548	3.0726	304.0573	2.9569
110022512113	12782.93	354.7391	2.7751	367.9567	2.8785
110022511425	12795.52	452.0784	3.5331	400.4741	3.1298
110022511457	11147.05	357.5306	3.2074	341.267	3.0615
110022511307	12385.06	378.7228	3.0579	369.6817	2.9849
110022511110	6251.602	193.4371	3.0942	178.3582	2.853
110022511552	11521.58	325.7495	2.8273	336.3839	2.9196
110022511355	13349.95	430.9231	3.2279	399.3238	2.9912
110022511549	12058.7	392.4262	3.2543	361.2304	2.9956
110022511500	10919.72	369.4468	3.3833	331.5991	3.0367
110022511497	10989.83	333.4096	3.0338	311.1771	2.8315
110022512093	11475.57	290.7222	2.5334	323.8177	2.8218
110022511496	13601.75	450.6395	3.3131	397.2662	2.9207

110022511536	12040.5	359.1079	2.9825	349.2829	2.9009
110022512133	12022.39	324.3039	2.6975	353.2418	2.9382
110022512126	10570.34	321.867	3.045	313.6855	2.9676
110022512108	10539.18	324.7648	3.0815	320.1908	3.0381
110022511488	10865.86	391.9099	3.6068	327.7796	3.0166
110022511448	10892.16	358.2323	3.2889	331.4266	3.0428
110022511510	11953.36	350.7355	2.9342	357.776	2.9931
110022511482	13430.43	360.0295	2.6807	399.0987	2.9716
110022511547	11740	394.8984	3.3637	355.6516	3.0294
110022511544	11447.4	369.9569	3.2318	350.897	3.0653
110022512073	13092.14	363.2152	2.7743	375.0898	2.865
110022512032	10973.05	337.6956	3.0775	344.2026	3.1368
110022511494	12143.53	434.2163	3.5757	385.5086	3.1746
110055120048	11047.76	328.8144	2.9763	312.6847	2.8303
110055011344	8007.998	212.4282	2.6527	194.1139	2.424
110055011269	12037.71	372.4106	3.0937	365.9343	3.0399
110055011343	10457.16	317.3854	3.0351	301.8565	2.8866
110055011323	9500.814	338.1245	3.5589	278.0128	2.9262
110055011212	9121.152	263.9205	2.8935	283.4307	3.1074
110055011316	9345.371	246.9888	2.6429	224.3169	2.4003
110055120016	9767.892	330.9752	3.3884	301.5641	3.0873
110055011308	10244.61	316.4152	3.0886	317.3577	3.0978
110055011292	9820.393	374.5302	3.8138	303.6171	3.0917
110055011284	10933.73	345.3854	3.1589	334.1893	3.0565
110055011280	10767.87	377.5431	3.5062	344.5826	3.2001
110055011325	9293.095	297.5742	3.2021	289.6936	3.1173
110055011367	9667.046	333.6678	3.4516	289.8084	2.9979
110055011390	9789.516	328.4676	3.3553	308.9669	3.1561
110055011348	11935.4	423.0025	3.5441	343.5127	2.8781
110055H12003	11474.52	352.4858	3.0719	341.5277	2.9764
110055011321	9853.785	300.1167	3.0457	284.3014	2.8852
110055011386	8670.339	296.0747	3.4148	260.6477	3.0062
110055011317	8982.253	260.8446	2.904	278.7013	3.1028
110055H11288	7750.232	269.0261	3.4712	230.4222	2.9731
110055011303	8410.407	296.0968	3.5206	262.3206	3.119
110055011377	8805.252	268.2608	3.0466	264.3953	3.0027
110055011330	11341	321.6535	2.8362	333.9358	2.9445
110055H11272	9925.782	339.1243	3.4166	299.3517	3.0159
110055120015	10721.05	318.9726	2.9752	312.0897	2.911
110055011267	9560.018	229.3831	2.3994	292.7947	3.0627
110055011327	11023.8	345.7503	3.1364	315.8648	2.8653
110055011268	9611.956	299.6143	3.1171	317.3868	3.302
110055011356	11319.08	408.1322	3.6057	329.9174	2.9147
110055011310	12648.47	384.1973	3.0375	378.6193	2.9934

110055011320	9604.049	299.4543	3.118	292.3184	3.0437
110055011339	10494.61	253.9695	2.42	261.3052	2.4899
110055011346	9630.113	334.7331	3.4759	299.641	3.1115
110055011351	10569.82	297.9526	2.8189	317.3588	3.0025
110055011181	12155.02	350.6602	2.8849	374.4233	3.0804
110055011301	10934.13	310.6933	2.8415	328.2754	3.0023
110055011326	7335.403	272.0701	3.709	227.889	3.1067
110055011328	10212	369.2863	3.6162	304.6954	2.9837
110055011300	10377.35	292.8281	2.8218	297.8611	2.8703
110055011359	10296.33	335.7223	3.2606	315.212	3.0614
110055H12008	8659.235	218.5937	2.5244	256.1835	2.9585
110055011337	6899.368	241.5607	3.5012	189.3256	2.7441
110055H11298	8065.096	300.6668	3.728	246.8806	3.0611
110055011385	10876.99	361.0399	3.3193	328.5939	3.021
110055011354	11924.07	374.2609	3.1387	360.3455	3.022
110055011368	10344.93	323.8999	3.131	298.3996	2.8845
110055120002	10707.56	364.0679	3.4001	330.596	3.0875
110055011304	11293.31	324.9536	2.8774	339.33	3.0047
110026211175	11458.82	445.7594	3.8901	352.7253	3.0782
110026211259	8143.5	301.6027	3.7036	249.2725	3.061
110032010296	11034.17	293.1117	2.6564	266.4753	2.415
110032011031	10220.67	276.275	2.7031	249.5173	2.4413
110032011268	10065.24	346.516	3.4427	295.8777	2.9396
110032011267	9854.61	297.0968	3.0148	323.2706	3.2804
110032011290	8679.663	304.4131	3.5072	275.9439	3.1792
110032011298	10931.57	309.1557	2.8281	326.7665	2.9892
110032011003	9725.651	263.3317	2.7076	226.3548	2.3274
110026211164	11126.51	410.6682	3.6909	347.3139	3.1215
110032012064	9015.934	266.1864	2.9524	262.3366	2.9097
110032120092	8696.596	307.729	3.5385	248.9488	2.8626
110032011260	10605.83	341.4441	3.2194	319.1507	3.0092
110032011281	10587.15	337.783	3.1905	308.0755	2.9099
110032010323	10123.05	290.7643	2.8723	257.9656	2.5483
110032011301	9964.701	299.8079	3.0087	304.9099	3.0599
110032011058	10206.41	284.2485	2.785	284.1362	2.7839
110032011274	9574.935	305.0287	3.1857	281.9052	2.9442
110032011259	10143.62	292.5421	2.884	314.9899	3.1053
110032011303	9209.133	243.425	2.6433	289.4062	3.1426
110026211178	12732.14	474.2721	3.725	393.3594	3.0895
110032120097	9444.949	307.5559	3.2563	281.2045	2.9773
110032011262	10278.07	308.3319	2.9999	306.3997	2.9811
110032012124	9294.226	322.6212	3.4712	283.7341	3.0528
110032012069	9294.674	299.084	3.2178	282.6789	3.0413
110032011273	9985.551	259.0651	2.5944	297.0402	2.9747

110032012063	9418.437	266.0897	2.8252	282.9393	3.0041
110032012071	8550.499	289.4002	3.3846	264.8346	3.0973
110032012117	6830.811	258.956	3.791	214.1459	3.135
110032012099	8508.397	336.8134	3.9586	274.0555	3.221
110032012119	9413.926	297.5742	3.161	288.2733	3.0622
110026211250	9982.25	369.503	3.7016	306.8544	3.074
110032011270	7649.501	265.9578	3.4768	239.896	3.1361
110032120096	6690.695	262.2619	3.9198	208.9571	3.1231
110032011261	9205.368	213.3068	2.3172	270.8864	2.9427
110032011282	10173.14	309.6296	3.0436	312.6612	3.0734
110026210210	14429.57	507.387	3.5163	422.5123	2.9281
110026811296	10288.02	340.1012	3.3058	296.7372	2.8843
110026120036	11143.2	419.4969	3.7646	331.8556	2.9781
110026211271	11065.69	404.6834	3.6571	346.1902	3.1285
110026211136	12272.9	438.4371	3.5724	379.7113	3.0939
110026120011	9345.531	379.3818	4.0595	293.1319	3.1366
110026210045	10039.88	316.0756	3.1482	305.9755	3.0476
110026210088	13497.03	372.1671	2.7574	327.7214	2.4281
110026120054	11442.04	467.9336	4.0896	367.3696	3.2107
110026120079	8622.412	342.2408	3.9692	267.7259	3.105
110026211276	11318.45	445.6072	3.937	340.4702	3.0081
110026211137	10802.46	397.671	3.6813	350.5722	3.2453
110026120048	11801.4	427.0455	3.6186	359.9545	3.0501
110026120039	10787.36	404.6337	3.751	336.7273	3.1215
110026211278	13104.58	399.6111	3.0494	380.6357	2.9046
110026120057	12159.66	426.9501	3.5112	368.9363	3.0341
110026120027	11216.18	452.9428	4.0383	345.5928	3.0812
110026120005	10050.79	366.5122	3.6466	299.6442	2.9813
110026211200	12278.81	495.4377	4.0349	391.1906	3.1859
110026211238	10522.06	411.7386	3.9131	345.2076	3.2808
110026211231	10852.79	393.3704	3.6246	330.0877	3.0415
110026211192	12308.8	493.5335	4.0096	425.9705	3.4607
110026120052	10450.41	404.828	3.8738	326.5649	3.1249
110026812009	10702.5	363.7138	3.3984	316.4301	2.9566
110026120004	13015.25	419.1039	3.2201	402.6526	3.0937
110026211236	10128.73	365.4446	3.608	335.1698	3.3091
110026120019	10428.23	402.8113	3.8627	320.543	3.0738
110026211253	11964.1	425.6709	3.5579	367.286	3.0699
110026120020	12120.3	470.195	3.8794	374.1416	3.0869
110026211268	10869.68	399.1019	3.6717	341.7752	3.1443
110026211275	11405.89	444.9209	3.9008	355.168	3.1139
110026210051	12445.81	472.9159	3.7998	384.4262	3.0888
110026120024	11619.08	432.0786	3.7187	356.4965	3.0682
110026211004	12248.36	429.5009	3.5066	375.2897	3.064

110026120017	10590.37	405.2934	3.827	317.6051	2.999
110026120044	12402.31	456.4671	3.6805	373.6693	3.0129
110026211285	9881.968	382.0863	3.8665	284.7193	2.8812
110026209249	13889.72	434.5676	3.1287	457.5273	3.294
110026211239	12929.48	487.3639	3.7694	399.4305	3.0893
110026211289	13195.76	438.4425	3.3226	379.2991	2.8744
110026211277	11686.22	471.1066	4.0313	363.3012	3.1088
110026210219	10353.11	282.961	2.7331	255.7012	2.4698
110026211233	10253.31	405.8363	3.9581	334.2375	3.2598
110026811208	14209.56	489.3346	3.4437	428.8019	3.0177
110026120007	11494.34	427.2216	3.7168	344.5543	2.9976
110026120055	12207.11	400.552	3.2813	352.5048	2.8877
110026211142	6057.964	209.5753	3.4595	183.8531	3.0349
110026120013	11357.14	406.5287	3.5795	339.6238	2.9904
110026120028	10939.66	385.9949	3.5284	328.6273	3.004
110026211251	10700.29	383.1881	3.5811	332.1798	3.1044
110026210206	14019.33	454.8572	3.2445	356.1752	2.5406
110026211230	11711.89	437.6381	3.7367	373.1173	3.1858
110026211258	12308.79	396.1337	3.2183	368.3405	2.9925
110033012066	8941.487	295.7308	3.3074	269.1656	3.0103
110033010067	11200.41	382.8301	3.418	335.8668	2.9987
110033011239	9140.158	261.4176	2.8601	270.8503	2.9633
110033011030	10337.26	359.2611	3.4754	300.2354	2.9044
110033011232	10408.13	395.686	3.8017	331.447	3.1845
110033011235	11865.13	371.1176	3.1278	363.3222	3.0621
110033012006	9546.075	311.8416	3.2667	278.5258	2.9177
110033011225	11179.56	348.9477	3.1213	343.358	3.0713
110033012034	10558.2	338.3271	3.2044	325.9106	3.0868
110033011228	12899.88	443.3948	3.4372	387.9124	3.0071
110033011237	11759.71	398.5482	3.3891	365.9856	3.1122
110033011234	12559.1	411.7502	3.2785	378.1797	3.0112
110033011230	12914.26	363.2652	2.8129	372.3181	2.883
110033011236	11718.66	433.7074	3.701	361.8252	3.0876
110033012021	10791.74	350.7747	3.2504	320.0074	2.9653
110033012018	9717.042	320.5458	3.2988	304.192	3.1305
110033012009	9072.287	343.5584	3.7869	270.6354	2.9831
110033012011	10793.38	390.8284	3.621	342.787	3.1759
110033011081	13515.36	411.1643	3.0422	415.7865	3.0764
110033011229	10472.86	272.5457	2.6024	322.6374	3.0807
110033012043	9779.77	343.71	3.5145	298.7231	3.0545
110033011242	9767.302	309.7993	3.1718	290.4014	2.9732
110033011220	12542.34	419.9302	3.3481	392.2869	3.1277
110033011219	13719.86	368.7899	2.688	404.8183	2.9506
110033011256	13993.57	428.917	3.0651	414.3637	2.9611

110033011248	12029.18	403.0858	3.3509	369.6808	3.0732
110033012023	10957.46	329.4031	3.0062	324.1654	2.9584
110033011053	11942.82	448.7277	3.7573	381.609	3.1953
110033012024	9277.496	306.779	3.3067	272.1553	2.9335
110033011231	9939.351	341.5658	3.4365	305.2375	3.071
110033011200	13141.51	346.5679	2.6372	397.0444	3.0213
110033011145	11925.82	410.6059	3.443	369.1399	3.0953
110033012025	10936.18	387.4031	3.5424	335.1391	3.0645
110033012058	6953.905	269.0674	3.8693	214.0064	3.0775
110033011241	10883.36	285.2745	2.6212	319.4809	2.9355
110033011188	11772.27	448.4412	3.8093	352.4383	2.9938
110033011080	11549.26	445.4203	3.8567	365.1183	3.1614
110033011086	11864.67	425.7755	3.5886	373.8795	3.1512
110050011271	12262.05	426.6089	3.4791	368.2661	3.0033
110050011291	12267.37	424.4509	3.46	417.6057	3.4042
110050011201	11490.11	385.1716	3.3522	356.1361	3.0995
110050011215	10308.3	361.5122	3.507	330.1337	3.2026
110050011250	10410.36	404.7549	3.888	325.9589	3.1311
110050011302	10828.36	414.3146	3.8262	328.4999	3.0337
110050011312	11742.54	339.5004	2.8912	316.6846	2.6969
110050011288	10872.67	389.7416	3.5846	332.9646	3.0624
110050011258	10913.3	416.7025	3.8183	346.7264	3.1771
110050011253	11375.98	382.688	3.364	374.8385	3.295
110050011315	12814.52	376.6315	2.9391	368.2124	2.8734
110050011307	10050.32	329.5199	3.2787	323.2787	3.2166
110050011249	10373.16	422.9863	4.0777	350.7788	3.3816
110050011276	9858.52	392.4677	3.981	301.9763	3.0631
110050011224	11612.41	428.6373	3.6912	372.2474	3.2056
110050N11162	11467.05	452.7076	3.9479	346.7865	3.0242
110050120041	11421.1	369.6553	3.2366	366.8914	3.2124
110050011275	11236.49	339.6117	3.0224	257.2032	2.289
110050011263	9348.537	339.5015	3.6316	314.5222	3.3644
110050011193	12318.12	463.0751	3.7593	381.6646	3.0984
110050011284	10774.14	365.8466	3.3956	327.4583	3.0393
110050120010	10832.41	397.7013	3.6714	332.7718	3.072
110050011310	12183.14	430.3937	3.5327	327.7264	2.69
110050011252	12041.72	412.0555	3.4219	387.2014	3.2155
110050011277	12683.02	420.6198	3.3164	391.4615	3.0865
110050011194	11553.4	486.9872	4.2151	371.3262	3.214
110050N11132	11211.22	445.6122	3.9747	341.7179	3.048
110050120001	10845.28	391.8617	3.6132	315.9772	2.9135
110050011261	11444.25	413.0458	3.6092	348.3286	3.0437
110050011325	11679.46	410.4163	3.514	346.2961	2.965
110050120043	11425.71	428.2013	3.7477	338.6466	2.9639

110050011257	9578.213	370.6385	3.8696	311.2823	3.2499
110050011232	13471.64	500.3503	3.7141	410.6022	3.0479
110050011327	10363.31	343.0983	3.3107	315.1277	3.0408
110050011297	12556.4	434.2002	3.458	355.2456	2.8292
110050011301	9713.051	319.404	3.2884	245.7693	2.5303
110050011326	11929.45	381.2892	3.1962	360.9495	3.0257
110050011286	11893.86	434.2212	3.6508	345.8735	2.908
110050011320	11663.51	430.9901	3.6952	364.4498	3.1247
110050120007	11753.54	434.0349	3.6928	363.0317	3.0887
110073011313	11204.57	380.171	3.393	342.4228	3.0561
110073440155	11237.18	319.3381	2.8418	290.0316	2.581
110073120010	10253.2	233.3732	2.2761	316.906	3.0908
110073011099	10388.65	409.6662	3.9434	308.9586	2.974
110073011316	9274.649	319.7713	3.4478	295.7964	3.1893
110073010220	9441.254	340.1684	3.603	314.5165	3.3313
110073011003	12051.33	337.5818	2.8012	295.9324	2.4556
110073120002	10867.1	368.1447	3.3877	340.2163	3.1307
110073011246	11647.56	481.5683	4.1345	390.8921	3.356
110073011312	10811.74	312.7513	2.8927	311.5512	2.8816
110073120012	9523.3	340.8008	3.5786	298.2317	3.1316
110073011281	10619.25	318.6626	3.0008	347.2284	3.2698
110073011200	11985.33	406.6982	3.3933	379.3716	3.1653
110073441440	9708.192	408.511	4.2079	313.3901	3.2281
110073011314	10439.99	281.932	2.7005	305.8187	2.9293
110073320313	6819.316	235.9824	3.4605	185.7241	2.7235
110073441278	12185.11	439.8093	3.6094	393.9689	3.2332
110073011301	6559.156	195.9351	2.9872	203.7733	3.1067
110073010170	9914.773	295.2619	2.978	243.259	2.4535
110073320322	6208.594	238.4472	3.8406	200.2396	3.2252
110073011303	9042.522	319.9606	3.5384	276.3304	3.0559
110073011215	12152.47	386.8253	3.1831	383.301	3.1541
110073011287	10498.08	374.0048	3.5626	335.6133	3.1969
110073441329	8604.155	364.5839	4.2373	290.4419	3.3756
110073011289	12808.98	430.3434	3.3597	371.1275	2.8974
110073440233	11179.87	346.0729	3.0955	250.0267	2.2364
110073011266	8527.808	346.519	4.0634	277.8189	3.2578
110073011305	12272.92	450.8947	3.6739	373.5262	3.0435
110073441279	10773.16	357.238	3.316	344.5041	3.1978
110073441460	10231.39	287.011	2.8052	332.643	3.2512
110073120081	11918.43	437.1801	3.6681	351.9752	2.9532
110073090169	11490.25	349.6941	3.0434	292.1165	2.5423
110073090167	11727.1	359.6114	3.0665	275.0004	2.345
110073441255	11542.25	371.4066	3.2178	360.603	3.1242
110019011476	10842.46	378.4559	3.4905	318.9634	2.9418

110019011525	9126.579	359.2495	3.9363	242.0004	2.6516
110019011550	9334.859	362.0338	3.8783	276.5732	2.9628
110019011526	10247.12	401.3385	3.9166	286.4479	2.7954
110019120091	9290.396	326.6503	3.516	275.6089	2.9666
110019011461	8324.475	337.8238	4.0582	254.2128	3.0538
110019011478	9126.252	366.8753	4.02	266.6326	2.9216
110019011370	9661.545	391.7177	4.0544	298.706	3.0917
110019011450	10432.55	371.2216	3.5583	291.8403	2.7974
110019011554	8367.999	330.9376	3.9548	257.8933	3.0819
110019011605	9450.65	351.9895	3.7245	258.1918	2.732
110019011290	9167.55	353.024	3.8508	270.9011	2.955
110019011574	9356.82	356.158	3.8064	277.2051	2.9626
110019011498	10533.68	389.7249	3.6998	331.874	3.1506
110019120149	8338.831	240.7087	2.8866	189.0246	2.2668
110019011508	9494.94	340.669	3.5879	276.4832	2.9119
110019011485	9679.312	319.7464	3.3034	268.3008	2.7719
110019011427	10413.4	468.0509	4.4947	353.5244	3.3949
110019120114	9512.055	377.0293	3.9637	288.9001	3.0372
110019011488	9588.82	315.9228	3.2947	271.0472	2.8267
110019011462	9782.697	407.1754	4.1622	291.8179	2.983
110019011546	9713.05	360.7912	3.7145	286.603	2.9507
110019120126	8640.47	331.3879	3.8353	249.9429	2.8927
110019011472	8737.749	278.7429	3.1901	265.7761	3.0417
110019011497	10380.55	385.762	3.7162	343.181	3.306
110019011072	13134.75	398.18	3.0315	364.3974	2.7743
110019011492	8919.714	385.5011	4.3219	263.0335	2.9489
110019011369	9781.271	355.9405	3.639	306.1342	3.1298
110019011487	9638.713	331.2826	3.437	294.5398	3.0558
110019010049	10801.27	395.402	3.6607	351.0412	3.25
110019011446	9617.342	370.4985	3.8524	283.0865	2.9435
110019011467	9400.51	380.26	4.0451	286.6498	3.0493
110019011522	7865.504	302.3972	3.8446	227.9423	2.898
110019120003	10102.96	393.8536	3.8984	296.8451	2.9382
110019011314	9756.638	433.8289	4.4465	279.674	2.8665
110019011576	9196.618	369.8236	4.0213	281.7752	3.0639
110019011475	9118.076	369.7927	4.0556	265.8284	2.9154
110019120049	8532.886	331.3746	3.8835	251.046	2.9421
110019011473	9602.013	382.8419	3.9871	278.6888	2.9024
110019011469	10506.33	429.6984	4.0899	329.3419	3.1347
110019011381	10599.66	410.4823	3.8726	328.2078	3.0964
110019011371	9401.397	360.9854	3.8397	284.8999	3.0304
110019011490	10840.73	436.4586	4.0261	318.338	2.9365
110019011227	7319.008	252.0447	3.4437	225.9963	3.0878
110019120171	9240.71	282.8951	3.0614	222.9414	2.4126

110019011577	9354.5	373.3662	3.9913	278.9231	2.9817
110015011475	11945.3	475.4706	3.9804	389.4645	3.2604
110015011513	13015.05	490.9797	3.7724	408.7115	3.1403
110015011509	11782.79	401.2512	3.4054	356.9362	3.0293
110015120073	10077.09	377.6088	3.7472	304.3685	3.0204
110015011505	10243.83	375.836	3.6689	328.6939	3.2087
110015011571	10866.08	455.7127	4.1939	361.1017	3.3232
110015011586	9241.045	390.8408	4.2294	304.0766	3.2905
110015011667	8070.004	262.7513	3.2559	261.3874	3.239
110015011560	13114.1	469.3144	3.5787	391.7051	2.9869
110015011548	13585.11	430.0231	3.1654	407.3903	2.9988
110015011533	10082.41	379.5219	3.7642	320.6205	3.18
110015120050	9998.069	397.0233	3.971	321.228	3.2129
110015011585	10021.1	429.725	4.2882	315.284	3.1462
110015011681	11139.55	447.5871	4.018	355.5298	3.1916
110015011598	9615.076	343.6524	3.5741	292.5002	3.0421
110015011652	11278.24	438.6897	3.8897	347.9901	3.0855
110015120028	10055.34	381.9118	3.7981	336.0795	3.3423
110015011211	7274.339	284.1939	3.9068	226.6029	3.1151
110015120007	11603.09	361.6566	3.1169	357.4679	3.0808
110015011676	11629.83	403.334	3.4681	363.1064	3.1222
110015011684	8533.61	315.8801	3.7016	257.8601	3.0217
110015120089	10221.48	345.3941	3.3791	302.2186	2.9567
110015011570	9070.67	341.9008	3.7693	292.6289	3.2261
110015011493	10853.75	382.4429	3.5236	351.7484	3.2408
110015011387	12242.5	406.1694	3.3177	394.2575	3.2204
110015011582	9500.062	371.7564	3.9132	275.8818	2.904
110015011669	10748.45	361.9432	3.3674	338.8663	3.1527
110015120022	8422.748	356.8718	4.237	289.4477	3.4365
110015011228	11204	433.987	3.8735	372.5218	3.3249
110015011603	10107.51	363.375	3.5951	314.6669	3.1132
110015011647	11887.21	407.3866	3.4271	354.6906	2.9838
110015011562	10763.95	412.1193	3.8287	330.4317	3.0698
110015120070	10728.79	360.2943	3.3582	323.8486	3.0185
110015011537	13114.66	480.1408	3.6611	423.0921	3.2261
110015011534	10906.28	406.4662	3.7269	344.8348	3.1618
110015011563	9032.513	301.5414	3.3384	289.5553	3.2057
110015011592	10164.3	315.6115	3.1051	325.4303	3.2017
110015011614	10923.35	427.1359	3.9103	355.719	3.2565
110015011645	9120.334	350.0293	3.8379	285.2567	3.1277
110015011685	7639.307	330.6598	4.3284	245.2447	3.2103
110015011500	9527.761	357.2529	3.7496	315.2927	3.3092
110015011530	10136.6	359.5553	3.5471	331.2336	3.2677
110015011595	9832.573	394.591	4.0131	316.6285	3.2202

110015011649	8321	326.9654	3.9294	262.2114	3.1512
110015120012	11483.54	379.0831	3.3011	370.6083	3.2273
110015011641	10446.12	356.8814	3.4164	341.5256	3.2694
110015011527	9869.901	379.3102	3.8431	323.0813	3.2734
110015011634	10919.75	392.8162	3.5973	340.6089	3.1192
110015011638	12209.04	406.0848	3.3261	380.2993	3.1149
110015011581	9104.701	350.6858	3.8517	282.2093	3.0996
110015011701	9224.474	316.7869	3.4342	308.3465	3.3427
110015010357	12065.03	428.0913	3.5482	369.8534	3.0655
110015011547	10156.32	285.9918	2.8159	313.2412	3.0842
110015011550	11871.78	451.5193	3.8033	366.4224	3.0865
110015011700	12389.91	436.8805	3.5261	403.4154	3.256
110015011267	11753.13	413.7337	3.5202	383.7045	3.2647
110015011339	10484.01	372.3814	3.5519	331.2736	3.1598
110015011698	10136.38	315.9915	3.1174	311.8964	3.077
110015011589	9295.464	407.067	4.3792	304.789	3.2789
110015011670	12382.64	441.9737	3.5693	386.5242	3.1215
110015011192	9619.763	367.6673	3.822	331.9203	3.4504
110015011554	7234.235	293.2252	4.0533	231.6908	3.2027
110015011686	9329.665	332.6305	3.5653	299.6222	3.2115
110015011528	11340.45	444.1261	3.9163	373.373	3.2924
110015120015	11542.49	384.2381	3.3289	355.9128	3.0835
110015011351	10846.16	446.3954	4.1157	360.3853	3.3227
110015011619	10588.77	371.147	3.5051	338.0571	3.1926
110015011610	8877.152	350.8517	3.9523	296.923	3.3448
110015011503	10789.83	390.333	3.6176	348.1232	3.2264
110015120045	8711.303	320.4366	3.6784	276.9933	3.1797
110015011661	10424.64	375.4851	3.6019	326.2391	3.1295
110015120080	9722.582	330.4414	3.3987	312.5616	3.2148
110015011696	10817.56	379.3609	3.5069	349.8614	3.2342
110015011658	7726.826	305.1633	3.9494	266.0114	3.4427
110015011519	9695.767	347.952	3.5887	315.316	3.2521
110015011542	8711.129	320.465	3.6788	287.0143	3.2948
110015011476	11039.8	427.3175	3.8707	354.3665	3.2099
110015011499	11751.98	384.5601	3.2723	365.0753	3.1065
110015011593	12396.35	498.5316	4.0216	407.3193	3.2858
110015011576	9839.574	373.8448	3.7994	316.8638	3.2203
110015011605	8580.296	334.0738	3.8935	284.8229	3.3195
110015011639	11615.02	333.0723	2.8676	343.9091	2.9609
110015120048	11585.64	312.0707	2.6936	357.5328	3.086
110015011556	10313.28	381.1479	3.6957	326.0338	3.1613
110015011526	8344.835	292.6617	3.5071	275.8969	3.3062
110015011622	12174.65	430.3009	3.5344	387.1782	3.1802
110015011394	10056.89	404.4278	4.0214	336.9762	3.3507

110015011099	9285	368.6424	3.9703	293.9445	3.1658
110015011511	11275.86	441.5287	3.9157	352.4382	3.1256
110015011569	9411.135	354.8092	3.7701	304.6573	3.2372
110015011693	10866.22	456.9465	4.2052	353.2392	3.2508
110015120030	11409.05	480.6975	4.2133	373.1672	3.2708
110015011642	11088.69	395.3229	3.5651	346.6546	3.1262
110015011704	11507.05	429.4546	3.7321	365.0497	3.1724
110016011001	8271.033	274.8382	3.3229	264.3339	3.1959
110016010278	9948.14	260.4423	2.618	252.9315	2.5425
110016120007	8350.626	309.3907	3.705	246.5689	2.9527
110016011271	8792.384	275.1665	3.1296	265.8905	3.0241
110016011201	9297.992	297.8147	3.203	284.2954	3.0576
110016011014	6907.406	184.8007	2.6754	180.5665	2.6141
110016011193	11930.18	460.1471	3.857	374.5481	3.1395
110016010247	11907.27	335.7135	2.8194	304.9213	2.5608
110016010295	10713.38	244.5328	2.2825	266.1952	2.4847
110016010290	9746.832	256.0395	2.6269	250.5228	2.5703
110016011249	9113.451	334.0718	3.6657	283.7382	3.1134
110016011251	8906.612	314.9645	3.5363	268.6234	3.016
110016011256	10150.5	384.8156	3.7911	301.3886	2.9692
110016011234	9144.273	327.1821	3.578	278.946	3.0505
110016011231	9630.611	381.4107	3.9604	313.5438	3.2557
110016011291	8289.07	299.5504	3.6138	249.3269	3.0079
110016011247	8760.758	336.781	3.8442	264.6888	3.0213
110016010258	9896.657	343.8395	3.4743	299.4629	3.0259
110016011257	8489.421	354.306	4.1735	263.9616	3.1093
110016011224	11162.58	410.4369	3.6769	345.426	3.0945
110016011011	10004.87	294.8135	2.9467	296.1741	2.9603
110016011289	9693.476	313.0993	3.23	282.9913	2.9194
110016011281	10660.5	321.9576	3.0201	317.6615	2.9798
110016011242	10646.11	366.7904	3.4453	300.71	2.8246
110016011237	12264.73	429.6704	3.5033	365.0107	2.9761
110016011278	10299.21	375.1691	3.6427	308.1728	2.9922
110016010294	9046.972	251.931	2.7847	221.4065	2.4473
110017T11107	10157.59	266.0475	2.6192	312.4575	3.0761
110017R11357	10506.81	370.5121	3.5264	345.1171	3.2847
110017T11326	7572.936	227.1805	2.9999	217.6235	2.8737
110017F11679	11197.45	361.8007	3.2311	342.709	3.0606
110017K11239	9914.032	343.9773	3.4696	310.6562	3.1335
110017K11174	10137.56	280.2833	2.7648	320.2962	3.1595
110017F12028	9302.828	324.7617	3.491	286.648	3.0813
110017T12046	7574.757	185.9376	2.4547	228.0305	3.0104
110017U11433	11366.86	279.6817	2.4605	352.1682	3.0982
110017L11301	8158.147	235.3054	2.8843	251.7849	3.0863

110017X11165	9594.445	324.4745	3.3819	305.8421	3.1877
110017T11329	9607.594	287.5745	2.9932	277.8612	2.8921
110017T11200	10190.58	304.1176	2.9843	306.441	3.0071
110017J11601	6505.426	260.0609	3.9976	212.3176	3.2637
110017F11397	9768.873	318.8072	3.2635	312.1155	3.195
110017X11239	9852.872	323.8836	3.2872	313.4198	3.181
110017T12054	11462.48	337.616	2.9454	349.6401	3.0503
110017D11217	9564.331	236.5355	2.4731	247.7353	2.5902
110017T11020	9054.573	296.0845	3.27	279.569	3.0876
110017F11559	10822.88	379.6449	3.5078	357.8693	3.3066
110017T11375	10803.68	346.9709	3.2116	321.7227	2.9779
110017T11407	10776.65	285.3334	2.6477	330.3043	3.065
110017K12133	10729.39	371.9022	3.4662	322.0105	3.0012
110017K11294	10482.54	342.433	3.2667	338.2295	3.2266
110017T11378	9124.503	293.8729	3.2207	314.3026	3.4446
110017T11459	9363.178	252.6935	2.6988	293.9383	3.1393
110017D11267	9335.865	266.6977	2.8567	287.6007	3.0806
110017K11436	11166.86	330.8181	2.9625	355.3071	3.1818
110017K11399	8424.459	282.5395	3.3538	251.2342	2.9822
110017F11351	9634.989	249.1801	2.5862	283.9335	2.9469
110017R11479	10876.19	335.2042	3.082	303.0433	2.7863
110017T12207	11401.78	337.5041	2.9601	325.3612	2.8536
110017F11013	9331.707	200.7064	2.1508	238.4998	2.5558
110017T11400	11866.9	358.2141	3.0186	374.7566	3.158
110017V11110	11765.88	356.3886	3.029	373.6138	3.1754
110017J12091	10398.24	281.2517	2.7048	308.9526	2.9712
110017K11463	9212.525	227.5678	2.4702	222.5285	2.4155
110017H11274	7294.148	241.4655	3.3104	228.4163	3.1315
110017T12017	10449.2	344.1235	3.2933	317.5199	3.0387
110017T11272	11060.96	315.3589	2.8511	346.7389	3.1348
110017H10286	9906.782	291.0216	2.9376	260.8456	2.633
110017X11219	7297.4	236.7933	3.2449	234.21	3.2095
110017710030	10214.81	406.1512	3.9761	345.6897	3.3842
110017F11087	9646.619	356.7995	3.6987	301.2446	3.1228
110017U12079	9168.077	308.3133	3.3629	274.8681	2.9981
110017N12121	9832.348	390.1574	3.9681	302.9641	3.0813
110017V11119	10025.72	360.3345	3.5941	302.5061	3.0173
110017T12001	9336.99	214.1159	2.2932	284.4701	3.0467
110017T11120	8814.943	251.6666	2.855	273.4043	3.1016
110017H11037	9653.467	343.2966	3.5562	309.7411	3.2086
110017T11271	7764.377	242.2718	3.1203	240.2298	3.094
110017T11116	10389.08	325.9574	3.1375	313.8126	3.0206
110017T11247	9210.038	282.9876	3.0726	243.0713	2.6392
110017T11379	9290.59	222.7233	2.3973	247.4549	2.6635

110017R12086	9486.309	274.2871	2.8914	237.1482	2.4999
110037011419	11678.85	434.3949	3.7195	383.7087	3.2855
110037120194	7510.533	250.7917	3.3392	227.1335	3.0242
110037120102	9390.301	295.9353	3.1515	287.3526	3.0601
110037120105	11193.17	373.8742	3.3402	339.2202	3.0306
110037011496	11490.17	370.719	3.2264	361.6647	3.1476
110037011509	11057.15	378.9286	3.427	338.9128	3.0651
110037011373	8631.139	327.7589	3.7974	280.0546	3.2447
110037011401	10440.52	331.1837	3.1721	317.3082	3.0392
110037120061	7844.389	272.5376	3.4743	235.112	2.9972
110037011433	11275.59	342.3044	3.0358	343.15	3.0433
110037011404	10536.44	334.7952	3.1775	317.3153	3.0116
110037120023	9983.59	316.969	3.1749	300.6558	3.0115
110037011517	13025.52	420.8806	3.2312	400.131	3.0719
110037011456	10174.44	304.6735	2.9945	331.2695	3.2559
110037011495	10166.5	354.4857	3.4868	316.1478	3.1097
110037120073	9306.456	302.5622	3.2511	291.897	3.1365
110037011407	11821	390.8141	3.3061	363.5785	3.0757
110037011497	10130.2	367.2399	3.6252	311.848	3.0784
110037011100	11403.9	413.5738	3.6266	369.8056	3.2428
110037011421	12298.37	437.3669	3.5563	377.0434	3.0658
110037011476	12773.5	434.3373	3.4003	386.8582	3.0286
110037011267	8202.959	265.1606	3.2325	245.1946	2.9891
110037120056	11241.52	385.6291	3.4304	343.4509	3.0552
110037010339	7503.678	226.9562	3.0246	233.0717	3.1061
110037011492	9521.604	277.7261	2.9168	286.6098	3.0101
110037011416	10162.91	357.2973	3.5157	326.1277	3.209
110037011487	10804.86	356.6468	3.3008	337.1657	3.1205
110037011500	10423.2	359.1105	3.4453	322.0664	3.0899
110037011486	10379.27	346.0136	3.3337	330.891	3.188
110037011431	8012.451	296.9414	3.706	256.8071	3.2051
110037011518	10840.47	345.8111	3.19	332.3255	3.0656
110037010326	10991.78	365.3229	3.3236	336.0298	3.0571
110037120026	10574.94	360.986	3.4136	325.4965	3.078
110037120032	12773.15	390.999	3.0611	393.9112	3.0839
110037011491	10984.23	362.0841	3.2964	339.7751	3.0933
110037120036	9734.175	287.6838	2.9554	285.0945	2.9288
110037011434	11555.51	401.5078	3.4746	356.4529	3.0847
110037011331	9076	256.0521	2.8212	224.032	2.4684
110037120096	8178.917	216.316	2.6448	195.8278	2.3943
110037011305	9706.5	345.9882	3.5645	314.0829	3.2358
110037120093	10463.63	279.8603	2.6746	263.2127	2.5155
110037011481	10460.16	367.7376	3.5156	325.8551	3.1152
110037120033	9408.456	310.6484	3.3018	281.1058	2.9878

110037120126	10047.55	307.696	3.0624	299.8891	2.9847
110037120066	10409.78	336.3818	3.2314	304.736	2.9274
110037011447	8896.409	306.8638	3.4493	278.7156	3.1329
110037120078	11625.99	391.1796	3.3647	359.0454	3.0883
110037011463	10827.92	323.1917	2.9848	318.4491	2.941
110037011022	9799.949	280.5333	2.8626	266.8624	2.7231
110037011358	12324.53	392.6595	3.186	386.4726	3.1358
110037120020	9772.171	325.7455	3.3334	306.1328	3.1327
110037120050	10377.78	330.055	3.1804	317.8507	3.0628
110037011453	10120.7	376.3889	3.719	323.2552	3.194
110037011508	10615.63	365.6236	3.4442	332.1419	3.1288
110037011526	11082.97	374.8262	3.382	347.7837	3.138
110037011393	9564.308	320.6243	3.3523	302.2417	3.1601
110037120052	9368.447	303.8562	3.2434	289.6443	3.0917
110037011398	9629.775	341.5874	3.5472	288.5851	2.9968
110037011505	9406.97	251.5706	2.6743	273.9498	2.9122
110037011435	9923.539	302.797	3.0513	305.2778	3.0763
110037120067	11133.93	351.3311	3.1555	365.8832	3.2862
110037011399	10155.39	331.6649	3.2659	310.2269	3.0548
110037011514	9409.645	315.1855	3.3496	300.657	3.1952
110037120069	10325	344.6588	3.3381	316.1515	3.062
110037011446	10058.82	328.7524	3.2683	320.8462	3.1897
110037011484	11382.18	386.4933	3.3956	357.6281	3.142
110037011450	8011.131	253.8006	3.1681	252.1824	3.1479
110037011442	8730.408	302.8753	3.4692	282.289	3.2334
110037011469	8973.324	316.9288	3.5319	285.9439	3.1866
110037011437	8583.558	279.7553	3.2592	264.8199	3.0852
110037011411	9873.992	339.9023	3.4424	315.3161	3.1934
110037120041	10775.21	354.2781	3.2879	339.4191	3.15
110037011309	12351.07	428.1374	3.4664	372.6935	3.0175
110037011451	9249.784	302.7824	3.2734	286.5028	3.0974
110037011485	10939.31	375.0105	3.4281	331.2423	3.028
110037011420	11703.65	384.4533	3.2849	363.2346	3.1036
110037120156	10970.59	351.2563	3.2018	323.1277	2.9454
110037011430	10101.64	300.7865	2.9776	322.6768	3.1943
110037011502	11460.01	357.621	3.1206	355.4207	3.1014
110037120054	10253.43	339.9319	3.3153	318.5843	3.1071
110038120047	9838.756	315.4207	3.2059	299.4032	3.0431
110038011175	12610.47	392.854	3.1153	385.5903	3.0577
110038120064	12203.11	421.6908	3.4556	346.3976	2.8386
110038120028	11401.98	400.2208	3.5101	359.5499	3.1534
110038011210	13464.22	409.0429	3.038	403.9265	3
110038120014	10724.5	298.3343	2.7818	356.8579	3.3275
110038010199	11591.29	284.5429	2.4548	298.6842	2.5768

110038011307	10708.82	338.8806	3.1645	325.1198	3.036
110038011183	13031.84	414.5819	3.1813	405.5899	3.1123
110038011034	11412.11	373.2443	3.2706	293.6449	2.5731
110038011297	12590.02	398.9904	3.1691	400.9166	3.1844
110038120070	11728.56	307.0537	2.618	347.8925	2.9662
110038120089	10884.07	374.3468	3.4394	333.2376	3.0617
110038011263	12111.01	422.868	3.4916	378.8445	3.1281
110038011191	10925.35	343.0121	3.1396	334.0752	3.0578
110038011148	11434.23	356.885	3.1212	345.0621	3.0178
110038120117	10125.33	334.3183	3.3018	301.0869	2.9736
110038011270	12554.02	393.7944	3.1368	387.8438	3.0894
110038011262	12419.02	421.6009	3.3948	382.9529	3.0836
110038011222	9928.704	299.6979	3.0185	310.5401	3.1277
110038011032	10630.11	376.4123	3.541	344.9365	3.2449
110038120111	8512.752	296.9333	3.4881	263.4952	3.0953
110038120057	10880.67	356.9949	3.281	338.2366	3.1086
110038011019	11867.24	287.2823	2.4208	292.8243	2.4675
110038120066	10007.12	393.8203	3.9354	342.844	3.426
110038011284	11279.74	360.1283	3.1927	361.7526	3.2071
110038120004	7984.019	262.0195	3.2818	249.9876	3.1311
110038011289	11764.45	433.3436	3.6835	378.5095	3.2174
110038120021	11583.05	358.7965	3.0976	363.395	3.1373
110038011300	14505.68	431.2247	2.9728	437.027	3.0128
110038011233	11980.06	369.0696	3.0807	377.1801	3.1484
110038011215	10874.26	365.4838	3.361	358.1981	3.294
110038011261	12258.84	399.7363	3.2608	392.3565	3.2006
110038011295	11150.54	331.3605	2.9717	342.0427	3.0675
110038011267	13706.42	416.0996	3.0358	426.4617	3.1114
110038011220	11895.46	422.0867	3.5483	367.3438	3.0881
110044120817	7771.107	286.2565	3.6836	241.1452	3.1031
110012011345	8082.85	275.2938	3.4059	232.3496	2.8746
110012011333	9944.659	356.8144	3.588	273.508	2.7503
110012911461	8415.662	212.5796	2.526	234.8559	2.7907
110012111077	8773.445	289.3921	3.2985	280.4168	3.1962
110012011230	10514.49	437.2873	4.1589	331.9426	3.157
110012111211	12749.09	438.1608	3.4368	362.9157	2.8466
110012111177	11709.13	295.9951	2.5279	318.4415	2.7196
110012011310	10753.92	426.8018	3.9688	305.8524	2.8441
110012111179	8999.374	309.7495	3.4419	261.3148	2.9037
110012011325	9658.511	310.8495	3.2184	284.7039	2.9477
110012120029	11136.92	380.3259	3.415	310.4194	2.7873
110012911443	9365.456	335.583	3.5832	292.9889	3.1284
110012120008	9478.826	294.2322	3.1041	290.9526	3.0695
110012011343	10083.21	348.6672	3.4579	287.6638	2.8529

110012912209	8869.868	334.1368	3.7671	252.6848	2.8488
110012011274	7891.917	265.9339	3.3697	237.7203	3.0122
110012711436	10590.23	365.2676	3.4491	307.9745	2.9081
110012112090	9279.675	316.3905	3.4095	279.8193	3.0154
110012111189	11884.47	399.2351	3.3593	357.1997	3.0056
110012911389	9652.608	325.428	3.3714	262.0007	2.7143
110012111187	10600	414.28	3.9083	327.3387	3.0881
110012111205	10667.95	385.6252	3.6148	308.7199	2.8939
110012111171	12847.6	417.033	3.246	362.7133	2.8232
110012111102	11054.73	394.1343	3.5653	350.5013	3.1706
110012111168	8256.256	280.9356	3.4027	269.7732	3.2675
110019011124	9864.21	402.588	4.0813	295.9855	3.0006
110019011596	9097.982	321.5955	3.5348	255.5623	2.809
110019120072	9811.648	356.83	3.6368	277.3066	2.8263
110064011284	10611.96	441.0226	4.1559	327.2623	3.0839
110064011244	10846.87	483.1195	4.454	331.4477	3.0557
110064011246	11276.32	434.7361	3.8553	349.0925	3.0958
110064120032	11961.43	436.616	3.6502	357.1802	2.9861
110064011191	10650.78	380.3286	3.5709	321.2487	3.0162
110064011278	7179.955	250.0276	3.4823	230.5053	3.2104
110064011245	11470.39	390.3717	3.4033	367.7521	3.2061
110064011049	13135	441.5461	3.3616	414.9478	3.1591
110064011287	9958.441	364.7279	3.6625	308.6917	3.0998
110064011304	11232.41	454.0251	4.0421	340.3644	3.0302
110064011057	10266.68	449.9477	4.3826	336.0902	3.2736
110064011248	6600.035	243.5083	3.6895	208.3433	3.1567
110064011223	10309.69	347.2096	3.3678	310.6309	3.013
110064011281	11292.85	442.454	3.918	349.3444	3.0935
110064011237	12366.33	537.9723	4.3503	369.0235	2.9841
110064120020	10854.27	367.5039	3.3858	348.1182	3.2072
110064011207	9100.04	387.8437	4.262	295.2508	3.2445
110064011202	12484.3	467.1	3.7415	378.7611	3.0339
110064011224	9532.808	373.7337	3.9205	307.8239	3.2291
110064011189	9611.129	343.7805	3.5769	310.3145	3.2287
110064011195	9368.173	337.5446	3.6031	295.5096	3.1544
110064011188	10472.55	419.0277	4.0012	344.8192	3.2926
110064011236	10831.41	401.5854	3.7076	337.8534	3.1192
110064011286	7856.732	299.5379	3.8125	241.9088	3.079
110064011227	11016.53	413.5606	3.754	338.0093	3.0682
110064120004	9837.517	401.8429	4.0848	316.1778	3.214
110064120022	8761.454	331.0691	3.7787	265.2881	3.0279
110064011242	12479.36	422.1143	3.3825	384.3767	3.0801
110064011300	10143.23	361.0382	3.5594	309.2266	3.0486
110064120048	10334.04	394.4709	3.8172	308.7707	2.9879

110064011307	9512.097	390.7474	4.1079	280.8066	2.9521
110064011269	10947.56	301.6273	2.7552	353.3983	3.2281
110064011238	9733.992	353.305	3.6296	311.1373	3.1964
110064120010	8924.715	399.5417	4.4768	281.9407	3.1591
110064120008	10724.87	338.6486	3.1576	344.6331	3.2134
110064011289	9119.955	337.2651	3.6981	284.3602	3.118
110064011213	10570.89	396.6198	3.752	340.5095	3.2212
110064011196	10184.73	464.4337	4.5601	321.1142	3.1529
110064011306	9565.699	272.0293	2.8438	289.8981	3.0306
110064011116	8908.63	337.6282	3.7899	275.3301	3.0906
110064011199	10367.24	393.862	3.7991	339.1851	3.2717
110064120023	11029.47	351.9394	3.1909	347.3401	3.1492
110064011295	9086.757	340.5081	3.7473	282.3528	3.1073
110064011183	7673.039	243.7187	3.1763	240.2121	3.1306
110064120001	10571.12	384.5879	3.6381	326.711	3.0906
110064011256	9829.22	466.082	4.7418	302.2878	3.0754
110064011103	10667.37	435.3781	4.0814	339.5104	3.1827
110064011265	8239.735	300.1406	3.6426	251.213	3.0488
110064011206	8901.433	373.1748	4.1923	284.4898	3.196
110064011268	10948.61	396.1427	3.6182	333.2429	3.0437
110010120052	12390.93	449.7411	3.6296	380.4387	3.0703
110010011029	7254.627	252.2216	3.4767	218.4441	3.0111
110010011100	12419.34	370.767	2.9854	372.2325	2.9972
110010011232	11225.22	352.4271	3.1396	334.2085	2.9773
110010011181	12757.68	470.5927	3.6887	381.0338	2.9867
110010120009	13147.64	452.2787	3.44	427.7978	3.2538
110010W10296	11452.1	385.9703	3.3703	317.0515	2.7685
110010W11006	8535.604	273.7795	3.2075	224.1364	2.6259
110010011034	10276.13	378.6445	3.6847	300.1965	2.9213
110010011252	12168.62	518.1762	4.2583	375.9494	3.0895
110010011114	10343.18	405.4838	3.9203	316.0567	3.0557
110010011174	11217.67	418.419	3.73	342.8231	3.0561
110010011108	11111.01	400.3518	3.6032	354.0078	3.1861
110010120043	11312.54	392.1832	3.4668	333.8558	2.9512
110010011160	11098.27	361.3153	3.2556	317.0998	2.8572
110010011230	10078.86	339.8793	3.3722	294.2422	2.9194
110010011220	13665.38	504.6215	3.6927	412.8448	3.0211
110010011227	12042.45	480.6622	3.9914	343.3181	2.8509
110010011251	12458.97	463.9721	3.724	383.6117	3.079
110010120003	12981.36	500.3534	3.8544	406.8747	3.1343
110010011185	12227.93	430.5453	3.521	383.6023	3.1371
110010011239	11878.04	379.8834	3.1982	344.0592	2.8966
110010011215	9894.731	316.1367	3.195	287.2638	2.9032
110010011184	8142.692	305.4324	3.751	269.8651	3.3142

110010010201	9623.646	256.1815	2.662	218.2162	2.2675
110010011112	11973.6	429.1817	3.5844	355.9153	2.9725
110010120006	12287.34	444.6298	3.6186	376.9142	3.0675
110010120005	11955.65	412.3502	3.449	383.0708	3.2041
110010011110	12373.33	420.5199	3.3986	364.1594	2.9431
110010011158	9839.281	421.1606	4.2804	297.3824	3.0224
110010011116	9351.118	362.786	3.8796	270.3034	2.8906
110010011226	11177.02	372.8094	3.3355	335.3888	3.0007
110071011387	10427.89	434.5926	4.1676	327.3522	3.1392
110071011098	6786.548	273.2671	4.0266	213.2944	3.1429
110071011427	7676.024	253.3625	3.3007	225.322	2.9354
110071011356	9654.547	357.421	3.7021	290.8625	3.0127
110071011491	9269.03	275.7814	2.9753	272.7041	2.9421
110071011417	9758.883	317.4467	3.2529	311.2303	3.1892
110071011422	8874.812	274.0808	3.0883	251.2725	2.8313
110071011413	12360.81	341.381	2.7618	353.9148	2.8632
110071011416	9446.572	306.0312	3.2396	295.0448	3.1233
110071011495	9250.875	315.7786	3.4135	274.9453	2.9721
110071011344	8989.648	268.9703	2.992	284.6482	3.1664
110071011388	11186.14	436.0471	3.8981	348.7504	3.1177
110071011336	8996.173	276.7763	3.0766	273.4747	3.0399
110071011466	9790.09	354.5677	3.6217	280.5057	2.8652
110071011488	9304.644	263.2005	2.8287	286.5179	3.0793
110071011316	9692.119	335.0372	3.4568	291.7328	3.01
110071011103	8976.401	350.6451	3.9063	280.7818	3.128
110071011441	10814.92	376.8025	3.4841	325.9292	3.0137
110071120609	8947.278	279.9335	3.1287	264.9826	2.9616
110071011412	9380.679	350.9875	3.7416	280.4073	2.9892
110071011219	8100.002	314.677	3.8849	241.5258	2.9818
110071011431	11119.7	404.9571	3.6418	316.0217	2.842
110071011443	11121.07	377.4268	3.3938	329.5728	2.9635
110071011415	11488.55	391.5527	3.4082	340.578	2.9645
110071010362	12146.44	438.7903	3.6125	274.1209	2.2568
110071011429	12450.13	394.8061	3.1711	349.6993	2.8088
110071011430	10112.18	287.7218	2.8453	272.9479	2.6992
110071011349	10355.17	331.2515	3.1989	308.263	2.9769
110071010481	10754.47	441.8905	4.1089	340.7554	3.1685
110071120606	8057.142	285.4645	3.543	261.5268	3.2459
110071011433	10163.89	369.1422	3.6319	312.7733	3.0773
110071011318	8719.924	324.2329	3.7183	280.8862	3.2212
110043120037	9842.518	247.9232	2.5189	241.25	2.4511
110043120123	9514.611	336.3034	3.5346	272.1464	2.8603
110043011328	10364.79	349.1483	3.3686	333.1243	3.214
110043011298	11681.62	349.0936	2.9884	297.2973	2.545

110043120025	9615.431	389.1365	4.047	316.9342	3.2961
110043011330	9476.268	342.3302	3.6125	301.5633	3.1823
110043011291	10623.8	387.9175	3.6514	338.6337	3.1875
110043z12009	11082.07	315.5286	2.8472	260.5061	2.3507
110043120109	11342.14	341.6026	3.0118	272.6197	2.4036
110043011296	6534.282	242.0167	3.7038	208.9075	3.1971
110043120061	8826.526	340.0331	3.8524	276.9676	3.1379
110043011380	10575.34	473.9234	4.4814	339.9867	3.2149
110043120016	11435.95	318.8113	2.7878	274.7258	2.4023
110043Z11133	6356.093	197.0071	3.0995	157.7328	2.4816
110043Z10029	10188.74	387.0294	3.7986	321.4445	3.1549
110043011416	11275.9	465.8976	4.1318	352.4733	3.1259
110043120121	11375.14	286.0051	2.5143	289.9523	2.549
110043120008	7076.496	251.2368	3.5503	225.8393	3.1914
110043011337	11173.28	419.5454	3.7549	348.5168	3.1192
110043011294	10718.04	354.6706	3.3091	281.8951	2.6301
110043120158	8936.727	238.3514	2.6671	219.8345	2.4599
110043011108	8598.055	264.3644	3.0747	218.3648	2.5397
110043011117	12278.04	480.2311	3.9113	366.6592	2.9863
110043120046	11250.72	334.405	2.9723	260.7691	2.3178
110043011396	9029.604	277.254	3.0705	280.4144	3.1055
110043120017	10904.93	393.5151	3.6086	324.3016	2.9739
110043Z11072	9514.647	343.4312	3.6095	299.5306	3.1481
110043011302	10086.01	384.6098	3.8133	342.9849	3.4006
110043011304	11121.65	395.5305	3.5564	363.0886	3.2647
110043Z11103	13328.36	347.8168	2.6096	304.9928	2.2883
110043120130	11091.05	359.35	3.24	252.9092	2.2803
110043120024	10777.97	332.231	3.0825	311.7744	2.8927
110043011288	9358.402	354.2249	3.7851	307.0211	3.2807
110043120011	6530.62	218.619	3.3476	201.868	3.0911
110043120029	11721.36	400.3548	3.4156	324.1543	2.7655
110043011303	9727.656	269.1837	2.7672	270.6526	2.7823
110043011418	11786.97	334.9268	2.8415	290.5489	2.465
110043Z11127	10300.58	253.1575	2.4577	244.0723	2.3695
110043120104	12066.86	390.0372	3.2323	350.482	2.9045
110043011402	11409.84	302.5434	2.6516	271.9993	2.3839
110043011386	10674.77	399.4712	3.7422	308.9385	2.8941
110043011327	6881.1	220.1195	3.1989	163.7014	2.379
110043Z10017	10000.82	273.1125	2.7309	234.6693	2.3465
110043120110	11645.84	405.2286	3.4796	372.7833	3.201
110043Z11169	9846.211	307.8319	3.1264	242.4039	2.4619
110043120082	11442.88	306.1199	2.6752	272.5923	2.3822

Table S4-4 Records of phenotypic values for dairy production traits of the second lactation.

ID	Milk yield (kg)	Fat yield (kg)	Fat percentage (%)	Protein yield (kg)	Protein percentage (%)
110022511408	11424.25	395.8387	3.4649	330.7319	2.895
110022511461	11668.69	386.2103	3.3098	350.4458	3.0033
110022110399	12507.7	417.7323	3.3398	390.7532	3.1241
110022512159	12615.04	508.1464	4.0281	369.6207	2.93
110022512001	12830.22	419.8562	3.2724	358.7587	2.7962
110022512077	11605.75	374.9586	3.2308	332.0405	2.861
110022512112	6466.68	220.2551	3.406	200.4347	3.0995
110022511522	10756.37	404.6223	3.7617	302.5444	2.8127
110022511556	10974.12	385.7404	3.515	344.6862	3.1409
110022512118	8838.566	319.1341	3.6107	252.0582	2.8518
110022511411	10408.67	412.4955	3.963	303.9435	2.9201
110022511512	12014.92	482.6395	4.017	351.5447	2.9259
110022512163	9262.214	301.976	3.2603	285.5818	3.0833
110022512010	5845.35	218.5576	3.739	169.6905	2.903
110022511405	11206.72	366.6615	3.2718	313.2279	2.795
110022512144	12058.04	412.8795	3.4241	345.5353	2.8656
110022512177	7052.582	263.4915	3.7361	202.8887	2.8768
110022511443	7791.201	317.9745	4.0812	258.9873	3.3241
110022511495	5606.634	205.0234	3.6568	162.3849	2.8963
110022511558	13904.32	508.62	3.658	404.0178	2.9057
110022511076	11545.38	361.4397	3.1306	351.2105	3.042
110022511458	11093.88	375.683	3.3864	341.0479	3.0742
110022511539	10837.65	355.3665	3.279	295.3151	2.7249
110022511425	6508.396	203.2637	3.1231	210.7223	3.2377
110022511307	13231.7	495.9769	3.7484	393.8282	2.9764
110022511110	10111.44	365.6802	3.6165	320.5629	3.1703
110022511355	6091.008	213.1061	3.4987	178.6493	2.933
110022511549	12799.09	428.5265	3.3481	392.4074	3.0659
110022511497	11600.82	397.1656	3.4236	346.5396	2.9872
110022512093	8665.904	245.4791	2.8327	247.8015	2.8595
110022511536	14425.11	541.288	3.7524	425.0215	2.9464
110022512133	9021.131	224.0578	2.4837	277.2464	3.0733
110022512126	12598.83	461.3945	3.6622	351.8603	2.7928
110022511488	12629.47	471.5971	3.7341	369.7657	2.9278
110022511448	11267.04	370.4377	3.2878	328.5243	2.9158
110022511510	10289.53	336.6218	3.2715	291.5537	2.8335
110022511482	9023.249	347.738	3.8538	262.5314	2.9095
110022511547	13368.07	509.7245	3.813	383.2091	2.8666
110022511544	12404.82	499.3435	4.0254	371.6111	2.9957
110022512032	11587.29	419.1702	3.6175	340.4925	2.9385
110022511494	13255.39	461.0091	3.4779	393.2608	2.9668

110022511428	13553.67	471.9389	3.482	398.6948	2.9416
110022511520	11496.6	459.8525	3.9999	340.5523	2.9622
110022110418	14509.35	512.0784	3.5293	431.4064	2.9733
110022511431	8139.84	294.4994	3.618	243.3649	2.9898
110022512164	7905.566	322.4601	4.0789	236.495	2.9915
110022511446	9743.75	307.8051	3.159	276.4107	2.8368
110022512003	13679.36	457.2052	3.3423	396.25	2.8967
110022511533	11588.65	454.0201	3.9178	341.1235	2.9436
110022512014	11719.58	432.9328	3.6941	330.0115	2.8159
110012011345	9541.108	336.2	3.5237	272.3032	2.854
110012011333	9542.838	302.0022	3.1647	242.6457	2.5427
110012911461	11287.27	403.4522	3.5744	324.78	2.8774
110012111077	7567.796	318.3923	4.2072	218.4217	2.8862
110012111211	9507.728	359.4872	3.781	286.7055	3.0155
110012111177	11164.04	347.1682	3.1097	310.2487	2.779
110012011310	8341.22	323.3891	3.877	228.3659	2.7378
110012111179	10719.45	417.7155	3.8968	290.7329	2.7122
110012011325	10199.53	384.1857	3.7667	306.9141	3.0091
110012911443	9023.633	360.3678	3.9936	253.6543	2.811
110012120008	14285.98	465.9088	3.2613	386.7645	2.7073
110012011343	11946.84	394.3294	3.3007	334.9655	2.8038
110012912209	8157.319	308.3793	3.7804	240.5022	2.9483
110012011274	7983.13	284.399	3.5625	239.909	3.0052
110012711436	13332.01	459.0745	3.4434	406.3597	3.048
110012111187	8354.563	415.4891	4.9732	217.5361	2.6038
110012111205	11253.77	438.1542	3.8934	338.5021	3.0079
110012111171	12282.98	436.267	3.5518	337.6469	2.7489
110012111102	11238.92	464.5945	4.1338	341.7756	3.041
110019011124	12620.54	408.3124	3.2353	354.3469	2.8077
110019011596	12599.97	434.8501	3.4512	384.2613	3.0497
110019011476	10775.13	315.2802	2.926	327.4023	3.0385
110019011525	11380.41	391.3266	3.4386	324.0457	2.8474
110019011461	9578.493	247.3454	2.5823	233.4279	2.437
110019011478	10016.62	420.1073	4.1941	301.0597	3.0056
110019011370	12070.66	371.3054	3.0761	369.2293	3.0589
110019011450	13908.78	478.9072	3.4432	389.8492	2.8029
110019011605	12056.63	365.4245	3.0309	355.7671	2.9508
110019011574	12587.56	435.1143	3.4567	367.9597	2.9232
110019120149	11211.25	394.1762	3.5159	352.5265	3.1444
110019011508	12573.11	439.3297	3.4942	344.9685	2.7437
110019011427	5794.36	195.1019	3.3671	165.1856	2.8508
110019011488	12758.41	390.3052	3.0592	365.286	2.8631
110037011100	11375.8	400.6443	3.5219	360.6014	3.1699
110037120020	9813.537	281.9625	2.8732	290.412	2.9593

110037011437	11862.66	427.9575	3.6076	360.886	3.0422
110037120105	10621.81	343.5305	3.2342	325.3672	3.0632
110037011496	12288.49	458.5203	3.7313	361.601	2.9426
110037011509	9877.729	392.1557	3.9701	314.3587	3.1825
110037011373	11387.53	410.7367	3.6069	348.9594	3.0644
110037011401	10693.1	355.6098	3.3256	307.6833	2.8774
110037011433	11368.28	391.4555	3.4434	349.2223	3.0719
110037011404	12016.74	437.5534	3.6412	361.5836	3.009
110037120023	9079.803	333.3559	3.6714	288.0295	3.1722
110037011517	11797.24	433.4542	3.6742	354.1531	3.002
110037011456	7437.66	250.8202	3.3723	234.9408	3.1588
110037011495	10526.91	403.3807	3.8319	309.7227	2.9422
110037011407	12957.48	489.4171	3.7771	389.2946	3.0044
110037011497	9782.082	361.3501	3.694	313.7505	3.2074
110037011421	5048.096	180.2927	3.5715	142.326	2.8194
110037011476	11703.48	367.5478	3.1405	333.9003	2.853
110037120056	7908.775	306.2594	3.8724	233.3879	2.951
110037011416	12152.46	379.0111	3.1188	366.9558	3.0196
110037011487	12022.78	442.2701	3.6786	356.7039	2.9669
110037011486	7398.065	257.9261	3.4864	212.3023	2.8697
110037011431	10337.64	367.8339	3.5582	306.8418	2.9682
110037010326	10284.39	413.6277	4.0219	332.2371	3.2305
110037120026	10121.43	308.2482	3.0455	285.6471	2.8222
110037120032	9145.422	371.487	4.062	265.9763	2.9083
110037120036	8465.887	298.8881	3.5305	254.3999	3.005
110037011434	12529.01	416.1133	3.3212	371.1467	2.9623
110037120096	9677.848	341.3667	3.5273	268.3764	2.7731
110037011305	12223.24	433.9371	3.5501	372.8821	3.0506
110037120093	13472.5	419.9513	3.1171	394.4344	2.9277
110037011481	10780	361.2486	3.3511	314.3771	2.9163
110037120033	9937.418	357.588	3.5984	289.6459	2.9147
110037120062	10556.71	347.6323	3.293	316.4162	2.9973
110037011447	10677.2	347.3508	3.2532	307.4074	2.8791
110037120078	12488.57	418.8292	3.3537	367.9257	2.9461
110037011463	12304.21	430.7335	3.5007	362.0145	2.9422
110037011022	10947.53	372.7964	3.4053	327.4736	2.9913
110037011358	14206.4	454.7611	3.2011	408.1357	2.8729
110037120050	12440.86	453.0586	3.6417	384.9947	3.0946
110037011453	7550.57	255.3376	3.3817	222.0849	2.9413
110037011393	10762.19	379.1519	3.523	329.5705	3.0623
110037120052	11333.57	388.5373	3.4282	334.7708	2.9538
110037011398	13347.08	477.4115	3.5769	404.8435	3.0332
110037011505	12150.31	398.9068	3.2831	359.0781	2.9553
110037011435	9183.124	312.9333	3.4077	273.4642	2.9779

110037011399	10278.13	356.4146	3.4677	317.8099	3.0921
110037011514	12160.94	385.514	3.1701	371.6384	3.056
110037120069	10690.98	396.058	3.7046	311.5458	2.9141
110037011446	13125.7	445.0794	3.3909	381.5247	2.9067
110037011484	14249.56	504.4487	3.5401	417.7971	2.932
110037011469	11288.41	431.1043	3.819	326.6639	2.8938
110037011411	10305.56	337.0639	3.2707	313.3508	3.0406
110037120041	11953.23	459.6616	3.8455	350.5883	2.933
110037011309	8938.213	303.3808	3.3942	291.8505	3.2652
110037011451	9620.804	336.68	3.4995	287.8448	2.9919
110037011485	10680.11	384.3984	3.5992	308.6123	2.8896
110037011430	11968.22	377.1068	3.1509	353.4456	2.9532
110037120054	10301.05	413.7932	4.017	301.6251	2.9281
110037011419	13347.36	452.2487	3.3883	408.4427	3.0601
110037120102	10623.75	390.8904	3.6794	307.409	2.8936
110019011462	10373.31	375.8977	3.6237	310.3591	2.9919
110019011546	13115.19	338.739	2.5828	302.6985	2.308
110019120126	12818.67	420.8498	3.2831	354.3978	2.7647
110019011497	10581.62	373.4252	3.529	318.8876	3.0136
110019011072	8601.397	286.6759	3.3329	259.1343	3.0127
110019011492	11726.38	321.2793	2.7398	276.1679	2.3551
110019011369	10284.84	293.6631	2.8553	317.997	3.0919
110019010049	11968.68	459.1067	3.8359	388.1564	3.2431
110019011446	14200.43	469.1111	3.3035	455.0243	3.2043
110019011467	11670.75	451.0393	3.8647	379.2875	3.2499
110019011522	12765.69	414.3231	3.2456	375.5409	2.9418
110019120003	11870.7	407.2007	3.4303	351.8833	2.9643
110019011314	13287.02	469.3107	3.5321	386.1605	2.9063
110019011576	13031.62	420.9342	3.2301	368.6775	2.8291
110019120049	10446.83	426.2934	4.0806	285.8253	2.736
110019011089	7975.002	301.9734	3.7865	245.1516	3.074
110019011469	10547.26	392.7484	3.7237	343.44	3.2562
110019011381	13318.76	444.1939	3.3351	412.1756	3.0947
110019011490	11197.23	328.9185	2.9375	334.4276	2.9867
110019011577	12216.17	399.3098	3.2687	328.2362	2.6869
110050120007	10542.62	340.1999	3.2269	312.4622	2.9638
110050011271	11788.75	451.9217	3.8335	337.453	2.8625
110050011291	12645.55	458.6161	3.6267	375.1175	2.9664
110050011201	11877.06	459.5355	3.8691	362.3811	3.0511
110050011215	10522.63	416.3174	3.9564	313.5744	2.98
110050011250	12076.58	471.7837	3.9066	362.7322	3.0036
110050011302	11058.09	414.0924	3.7447	323.7478	2.9277
110050011288	11610.41	408.9765	3.5225	345.2122	2.9733
110050011258	12254.69	487.4792	3.9779	376.1943	3.0698

110050011253	8604.666	330.0922	3.8362	287.1205	3.3368
110050011315	13026.88	409.0311	3.1399	375.7344	2.8843
110050011307	10151.3	305.7571	3.012	286.9569	2.8268
110050011249	10530.25	456.9393	4.3393	343.6232	3.2632
110050011276	6552.01	269.9821	4.1206	206.5259	3.1521
110050011224	10974.59	431.2577	3.9296	327.021	2.9798
110050N11162	12587.3	488.0725	3.8775	389.3251	3.093
110050011193	13866.01	556.0962	4.0105	410.101	2.9576
110050011284	13511.05	523.8099	3.8769	413.6678	3.0617
110050011310	12601.46	411.992	3.2694	335.917	2.6657
110050011252	12952.79	465.3549	3.5927	400.1505	3.0893
110050011277	11139.81	413.7771	3.7144	338.7059	3.0405
110050011194	12723.63	536.5557	4.217	402.3977	3.1626
110050N11132	10588.97	386.3811	3.6489	326.8604	3.0868
110050120001	8231.286	312.4349	3.7957	244.5021	2.9704
110050011325	11248.17	444.2689	3.9497	324.1835	2.8821
110050120043	10808.55	354.2393	3.2774	309.0488	2.8593
110050011257	9598.886	383.3507	3.9937	317.7615	3.3104
110050011232	12460.53	490.247	3.9344	375.012	3.0096
110050011327	12258.76	432.7464	3.5301	379.9357	3.0993
110050011301	10070.39	307.7915	3.0564	291.7091	2.8967
110050011326	12159.16	451.4819	3.7131	371.1585	3.0525
110050011286	10884.05	359.2716	3.3009	308.8893	2.838
110050011320	11338.89	457.3655	4.0336	352.9683	3.1129
110024011336	10154.57	314.0302	3.0925	308.9021	3.042
110024011270	9880.443	321.7961	3.2569	276.3461	2.7969
110024011126	10933.07	400.2267	3.6607	336.2027	3.0751
110024011297	8145.255	288.0895	3.5369	247.2737	3.0358
110024011333	9231.078	421.6295	4.5675	254.3346	2.7552
110024011253	8682.324	329.9196	3.7999	257.5091	2.9659
110015011667	10643.35	424.4673	3.9881	354.6682	3.3323
110024011201	9787.536	344.8932	3.5238	283.4862	2.8964
110024011308	9827.578	410.2424	4.1744	305.048	3.104
110024120032	7749.63	227.6376	2.9374	228.8853	2.9535
110024011300	10099.32	353.2034	3.4973	299.7073	2.9676
110024120008	10790.44	420.169	3.8939	311.3582	2.8855
110024011343	10161.05	404.5826	3.9817	315.2974	3.103
110024011295	8357.922	358.9226	4.2944	274.7332	3.2871
110024011028	10245.95	361.9279	3.5324	308.5875	3.0118
110024011335	10515.32	375.8282	3.5741	301.6847	2.869
110024010118	9662.071	378.4826	3.9172	287.321	2.9737
110024011339	9518.363	375.528	3.9453	293.7272	3.0859
110024011283	7923.757	321.7917	4.0611	258.1402	3.2578
110026210045	11497.19	397.4119	3.4566	366.6339	3.1889

110026120019	10638.89	326.1457	3.0656	306.1765	2.8779
110026209249	12658.5	453.4528	3.5822	397.3123	3.1387
110026211142	7136.637	228.1012	3.1962	209.1035	2.93
110026210206	14331.58	519.3766	3.624	451.5452	3.1507
110026211230	11549.64	337.4458	2.9217	348.0484	3.0135
110026211258	12860.39	434.3852	3.3777	381.1047	2.9634
110026211259	8402.115	332.7154	3.9599	248.711	2.9601
110026211164	13417.22	451.342	3.3639	389.7704	2.905
110026211178	8535.256	265.2416	3.1076	224.2639	2.6275
110026210210	13619.99	393.2636	2.8874	395.8105	2.9061
110026811296	10710.31	287.1221	2.6808	292.9164	2.7349
110026211271	10066.69	348.1463	3.4584	317.906	3.158
110026211136	11105.26	424.4318	3.8219	330.8256	2.979
110026210088	12970.72	370.0807	2.8532	402.1962	3.1008
110026211276	9576.278	294.2503	3.0727	280.0199	2.9241
110026120048	13166.79	489.4886	3.7176	397.0972	3.0159
110026120057	11906.9	331.4761	2.7839	356.8259	2.9968
110026120005	8233.063	341.1452	4.1436	271.3864	3.2963
110026211200	11330.7	399.5092	3.5259	374.0944	3.3016
110026211231	11905.43	419.9641	3.5275	369.6279	3.1047
110026211192	9768.945	404.1706	4.1373	345.6644	3.5384
110026812009	8828.872	314.8552	3.5662	269.21	3.0492
110026120004	15311.31	541.9132	3.5393	448.2845	2.9278
110026810212	12436.36	357.9059	2.8779	373.2399	3.0012
110026211253	13008.7	442.1398	3.3988	399.2632	3.0692
110026120020	13285.42	457.9219	3.4468	394.4708	2.9692
110026211268	11516	476.1289	4.1345	355.3031	3.0853
110026211275	9645.563	328.3446	3.4041	280.329	2.9063
110026211004	12109.4	326.1305	2.6932	374.8466	3.0955
110026120017	8314.328	271.2383	3.2623	250.9015	3.0177
110026211239	8965.388	331.9435	3.7025	249.3005	2.7807
110026211277	10286.77	280.4174	2.726	294.4382	2.8623
110026210219	11778.67	462.3597	3.9254	371.7818	3.1564
110026211233	5523.288	198.8826	3.6008	171.4097	3.1034
110026811208	11403.42	339.6852	2.9788	309.6029	2.715
110026811014	11729.99	351.0318	2.9926	343.2079	2.9259
110026120007	11719.9	373.736	3.1889	344.5534	2.9399
110026811092	12277.5	412.7941	3.3622	369.9088	3.0129
110015011586	10997.99	331.3475	3.0128	262.1371	2.3835
110026120013	8918.226	322.7774	3.6193	264.1489	2.9619
110026120028	10787.5	402.9564	3.7354	320.0437	2.9668
110046011147	11054.61	442.6266	4.004	334.6783	3.0275
110046011275	12884.87	431.3211	3.3475	360.0291	2.7942
110046120028	9513.562	502.0212	5.2769	313.6146	3.2965

110046011253	15255.92	515.7263	3.3805	433.1766	2.8394
110046011155	12467.25	614.2366	4.9268	403.4652	3.2362
110046120051	10843.4	335.2671	3.0919	270.0223	2.4902
110046011179	11069.17	601.1999	5.4313	347.8376	3.1424
110046011259	10921.51	339.3532	3.1072	323.5061	2.9621
110046011144	14483.93	741.9247	5.1224	441.137	3.0457
110046120006	12936.74	499.6167	3.862	396.9637	3.0685
110046120023	12490.23	556.6394	4.4566	365.7762	2.9285
110046011178	14386.5	608.5489	4.23	433.6378	3.0142
110046011037	11180.86	368.6554	3.2972	342.2798	3.0613
110046120126	8931.232	413.257	4.6271	241.7149	2.7064
110046011153	6986.821	318.3335	4.5562	230.4463	3.2983
110046011189	11902.73	591.0895	4.966	387.9575	3.2594
110046011138	7574.182	360.978	4.7659	230.1643	3.0388
110046120089	8283.775	316.8461	3.8249	231.6392	2.7963
110046011148	10500	476.6056	4.5391	304.5106	2.9001
110046120018	7495.315	354.2136	4.7258	217.9787	2.9082
110046011145	12514.1	544.8262	4.3537	382.7937	3.0589
110046011040	12651.86	419.5231	3.3159	403.1769	3.1867
110055011303	11338.54	455.3671	4.0161	356.7558	3.1464
110055H12008	10355.84	321.1553	3.1012	303.9957	2.9355
110055011354	12298.28	392.7456	3.1935	343.2204	2.7908
110055120002	13907.48	474.6621	3.413	422.5369	3.0382
110055011344	9437.334	373.3976	3.9566	288.32	3.0551
110055011269	12686.59	384.6066	3.0316	338.5543	2.6686
110055011343	9844.448	279.2968	2.8371	289.4465	2.9402
110055011323	9586.213	366.9315	3.8277	296.9234	3.0974
110055011316	12371.54	486.7087	3.9341	397.3986	3.2122
110055120016	8876.197	295.3821	3.3278	274.239	3.0896
110055011308	11652.69	363.0512	3.1156	369.9379	3.1747
110055011325	12069.24	450.6535	3.7339	354.9806	2.9412
110055011367	13482.48	436.1852	3.2352	381.4193	2.829
110055011390	11980.01	280.0567	2.3377	409.0454	3.4144
110055011321	6556.512	145.0497	2.2123	212.4572	3.2404
110055011386	11515.12	405.1826	3.5187	326.2465	2.8332
110055H11288	11881.73	374.9993	3.1561	344.986	2.9035
110055011306	11641.43	357.3104	3.0693	353.8995	3.04
110055011330	8494.517	217.4681	2.5601	212.6093	2.5029
110055H11272	8871.562	253.2831	2.855	262.7668	2.9619
110055120015	12023.25	377.1213	3.1366	339.3923	2.8228
110055011327	12668.87	438.6216	3.4622	368.0053	2.9048
110055011268	9944.34	274.3146	2.7585	326.4528	3.2828
110055011310	14416.43	502.4269	3.4851	441.0706	3.0595
110055011351	10912.61	368.2461	3.3745	318.8228	2.9216

110055011326	9506.272	303.7349	3.1951	280.5586	2.9513
110055011328	10613.12	250.2254	2.3577	302.9408	2.8544
110055011300	12223.9	379.5644	3.1051	323.0533	2.6428
110055011337	7212.072	234.5798	3.2526	203.467	2.8212
110055011385	12070.89	395.1647	3.2737	359.3383	2.9769
110010W10296	12722.96	376.1161	2.9562	386.867	3.0407
110010W11006	12013.34	415.5536	3.4591	356.496	2.9675
110010011034	11634.55	423.4861	3.6399	364.2197	3.1305
110010011252	10386.82	426.9816	4.1108	308.738	2.9724
110010011114	11361.91	437.1495	3.8475	330.0067	2.9045
110010011108	9059.101	353.314	3.9001	278.0691	3.0695
110010011251	12368.24	449.697	3.6359	385.3574	3.1157
110010010197	11708.65	403.7261	3.4481	358.0155	3.0577
110010120003	13011.47	493.7333	3.7946	367.3139	2.823
110010011185	10526.37	322.3068	3.0619	306.412	2.9109
110010011239	14647.81	469.6087	3.206	415.3093	2.8353
110010011215	11014.12	375.879	3.4127	304.7057	2.7665
110010011184	9887.174	377.9965	3.8231	313.6706	3.1725
110010011112	12558.8	494.7915	3.9398	374.3024	2.9804
110010120005	10146.83	373.1803	3.6778	337.1894	3.3231
110010011110	8835.84	299.2346	3.3866	254.0481	2.8752
110010010210	15004.05	527.1373	3.5133	435.1325	2.9001
110010011116	9881.179	370.732	3.7519	283.4317	2.8684
110010011226	12213.49	417.6526	3.4196	372.1818	3.0473
110010011029	11305.25	371.4002	3.2852	345.6694	3.0576
110010011100	10410.98	376.5549	3.6169	300.284	2.8843
110010011232	12171.35	405.1114	3.3284	352.7015	2.8978
110010011181	9300.138	366.537	3.9412	283.0776	3.0438
110071011387	10176.37	455.6671	4.4777	282.9233	2.7802
110071011098	11114.68	391.1924	3.5196	301.7859	2.7152
110071011427	9014.509	275.5014	3.0562	271.3818	3.0105
110071011356	8832.173	360.1848	4.0781	272.4195	3.0844
110071011491	9773.109	304.6278	3.117	286.1469	2.9279
110071011417	8895.194	269.0885	3.0251	271.677	3.0542
110071011413	13885.27	440.5657	3.1729	425.4863	3.0643
110015011560	12777.66	505.6757	3.9575	378.7425	2.9641
110071011421	10223.8	340.4629	3.3301	329.1451	3.2194
110071011495	8376.255	292.3983	3.4908	262.5956	3.135
110071011388	11590.54	421.1074	3.6332	345.5255	2.9811
110071120005	6492.428	224.19	3.4531	188.7673	2.9075
110071011316	10600.23	398.3673	3.7581	324.6957	3.0631
110071011103	11780.03	448.9959	3.8115	361.5527	3.0692
110071011441	10921.82	420.8834	3.8536	318.6551	2.9176
110071011412	10371.2	405.5036	3.9099	312.6087	3.0142

110071011219	6685.209	231.1344	3.4574	197.6816	2.957
110071011431	11892.73	374.8351	3.1518	337.6584	2.8392
110071010362	15376.3	546.8579	3.5565	414.222	2.6939
110071011429	10681.4	366.9274	3.4352	301.6855	2.8244
110071010481	11098.85	428.4267	3.8601	339.081	3.0551
110071011433	12351.17	429.6848	3.4789	337.7056	2.7342
110064011295	11029.93	571.2512	5.1791	314.2318	2.8489
110064011103	14250.58	648.5868	4.5513	416.5161	2.9228
110064011265	7833.22	264.4887	3.3765	234.7929	2.9974
110064011246	11756.05	551.0178	4.6871	333.4838	2.8367
110064011245	10991	537.7129	4.8923	351.1077	3.1945
110064011223	13037.74	618.8622	4.7467	328.6161	2.5205
110064120020	13121.05	517.5468	3.9444	404.3252	3.0815
110064011207	12386.23	658.2539	5.3144	411.7927	3.3246
110064011224	13728.16	712.7935	5.1922	401.2055	2.9225
110064011189	13492.98	621.5676	4.6066	416.8386	3.0893
110064120004	11942.66	584.7247	4.8961	275.0634	2.3032
110064120022	11583.56	596.3797	5.1485	343.7075	2.9672
110064011300	13393.01	511.3048	3.8177	324.7536	2.4248
110064011307	8567.688	308.5139	3.6009	256.1739	2.99
110064011277	8980.178	364.0564	4.054	263.5143	2.9344
110064011213	12759.56	604.7392	4.7395	381.9956	2.9938
110064011116	9633.283	409.2508	4.2483	263.6726	2.7371
110064011199	11134.86	593.143	5.3269	349.8017	3.1415
110064120023	12280.34	564.773	4.599	368.1156	2.9976
110064011183	9008.588	276.3384	3.0675	253.2584	2.8113
110064011256	11896.21	569.0314	4.7833	336.0917	2.8252
110033010067	13119.38	435.7339	3.3213	399.3145	3.0437
110033011239	12319.84	360.8973	2.9294	368.3877	2.9902
110033011030	12429.53	422.8525	3.402	371.6056	2.9897
110033011232	6768.971	279.4028	4.1277	221.6432	3.2744
110033011235	14074.8	434.1514	3.0846	417.2898	2.9648
110033012006	11078.55	366.2124	3.3056	329.2876	2.9723
110033012034	12865.47	520.241	4.0437	377.4343	2.9337
110033011237	9907.92	412.754	4.1659	311.8022	3.147
110015011533	7994.673	319.7869	4	240.4558	3.0077
110033011230	11591.09	375.9801	3.2437	352.3575	3.0399
110033011236	13031.93	636.4535	4.8838	451.7389	3.4664
110033012018	13073.41	428.2718	3.2759	354.4071	2.7109
110033012009	12549.52	493.2214	3.9302	376.1846	2.9976
110033011081	12139.74	444.5938	3.6623	381.5157	3.1427
110033011229	12549.38	389.7963	3.1061	382.5176	3.0481
110033012043	10113.42	457.6323	4.525	318.593	3.1502
110033011220	13213.16	541.5546	4.0986	394.4525	2.9853

110033011256	12478.8	478.5369	3.8348	394.8541	3.1642
110033011248	13567.69	519.0455	3.8256	420.7612	3.1012
110033012023	9507.125	343.1216	3.6091	281.2398	2.9582
110033011053	10377.57	405.7214	3.9096	325.9698	3.1411
110033012024	13603.37	433.9747	3.1902	405.122	2.9781
110033012025	9300.331	341.1919	3.6686	304.2603	3.2715
110033011188	10465.58	465.6137	4.449	332.4497	3.1766
110033011080	11341.65	426.049	3.7565	350.8426	3.0934
110033011086	10939.16	395.3848	3.6144	360.117	3.292
110043120037	11122.9	274.3464	2.4665	244.626	2.1993
110043011416	13231.21	311.304	2.3528	318.6076	2.408
110043Z11072	11107.17	256.4868	2.3092	262.1515	2.3602
110043120082	9343.486	362.6954	3.8818	307.6716	3.2929
110043011298	12174.49	360.4013	2.9603	291.4694	2.3941
110043120025	9773.376	385.0124	3.9394	287.3861	2.9405
110043011291	12507.23	433.2506	3.464	344.7619	2.7565
110043120129	9456.907	212.9223	2.2515	304.7867	3.2229
110043011296	8094.944	239.9584	2.9643	216.1269	2.6699
110043120061	11129.36	406.0881	3.6488	335.7728	3.017
110043011380	11082.8	347.0246	3.1312	249.6401	2.2525
110043120016	12802.4	440.902	3.4439	404.1719	3.157
110043Z10029	8556.066	291.8816	3.4114	253.9098	2.9676
110043120121	12552.28	416.0706	3.3147	394.3802	3.1419
110043011294	9165.123	304.4837	3.3222	235.8919	2.5738
110043120158	11649.31	394.9117	3.39	355.2924	3.0499
110043011108	10557.41	322.2439	3.0523	312.3728	2.9588
110043011117	13692.17	449.8152	3.2852	348.411	2.5446
110043120046	13422.07	501.7304	3.7381	410.7556	3.0603
110043011396	9167.745	278.6261	3.0392	210.3906	2.2949
110043120105	10982.12	358.3686	3.2632	328.2447	2.9889
110043011302	9674.881	300.7727	3.1088	259.2481	2.6796
110043011304	11056.79	414.8507	3.752	330.8412	2.9922
110043Z11103	10532.65	397.576	3.7747	314.3785	2.9848
110043120130	11054.98	385.3325	3.4856	311.3194	2.8161
110043011288	8946.613	385.5901	4.3099	271.6728	3.0366
110043120029	12670.79	440.0945	3.4733	357.2275	2.8193
110043011303	9318.4	225.8035	2.4232	221.0604	2.3723
110043011418	12623.97	349.8355	2.7712	293.722	2.3267
110043Z11127	9856.912	318.6345	3.2326	292.287	2.9653
110043120104	11986.17	425.8806	3.5531	353.4122	2.9485
110017T11407	10206.39	318.0006	3.1157	284.2582	2.7851
110017K12133	10393.76	275.4243	2.6499	312.6131	3.0077
110017T11459	9724.963	304.5469	3.1316	262.5351	2.6996
110017K11436	9048.864	347.9379	3.8451	275.8818	3.0488

110017K11399	7234.718	285.5833	3.9474	221.8526	3.0665
110017R11479	13121.32	462.4742	3.5246	370.2313	2.8216
110017T12207	12169.47	438.8677	3.6063	331.6668	2.7254
110017F11013	9375.927	279.5808	2.9819	291.3382	3.1073
110017V11110	10957.89	394.473	3.5999	350.9483	3.2027
110017J12091	9950.148	317.0913	3.1868	267.3306	2.6867
110017K11463	6687.693	282.2474	4.2204	224.9539	3.3637
110017H11274	8415.202	305.4718	3.63	241.0871	2.8649
110017T12017	9380.998	284.2161	3.0297	263.6811	2.8108
110017T11272	10819.88	376.0559	3.4756	317.3256	2.9328
110017H10286	11427.59	334.7256	2.9291	341.5593	2.9889
110017X11219	4925.971	180.7634	3.6696	137.8582	2.7986
110017710030	9776.979	300.8768	3.0774	307.6815	3.147
110017F11087	9405.642	356.2387	3.7875	291.8665	3.1031
110017P12088	7106.247	232.7722	3.2756	216.1791	3.0421
110017T12001	9215.705	229.9318	2.495	267.1541	2.8989
110017T11120	8811.27	209.4263	2.3768	246.6098	2.7988
110017R12086	7265.374	325.4161	4.479	230.0871	3.1669
110017R11357	10971.25	407.4502	3.7138	328.2268	2.9917
110017K11239	9238.645	273.1775	2.9569	250.7461	2.7141
110017K11174	8373.666	200.3986	2.3932	204.912	2.4471
110017F12028	10106.94	411.13	4.0678	292.0804	2.8899
110017T12046	8704.955	311.7767	3.5816	269.4184	3.095
110017U11433	10279.99	371.9507	3.6182	314.5883	3.0602
110017X11165	10056.99	372.2694	3.7016	305.9738	3.0424
110017T11329	11749.12	417.3289	3.552	312.3152	2.6582
110017T11200	9539.042	249.4078	2.6146	262.7434	2.7544
110017F11397	12682.07	451.3675	3.5591	385.7885	3.042
110017T11390	7328.817	245.3468	3.3477	208.3509	2.8429
110017F11559	11502.78	403.9316	3.5116	341.9891	2.9731
110073011313	11036.44	423.4903	3.8372	341.622	3.0954
110073440155	10623.31	325.5512	3.0645	349.5174	3.2901
110073120010	12366.21	329.9553	2.6682	362.7505	2.9334
110073011099	9400.134	373.6553	3.975	278.4978	2.9627
110073010220	10768.43	360.6779	3.3494	338.398	3.1425
110073011003	11577.34	394.2548	3.4054	402.1506	3.4736
110073120002	10769.25	363.7961	3.3781	352.2299	3.2707
110073011246	10619.79	398.0192	3.7479	317.4893	2.9896
110073011312	11626.13	334.4373	2.8766	303.1165	2.6072
110073120012	8429.713	322.6472	3.8275	255.9429	3.0362
110073011200	10986.72	422.4285	3.8449	339.3908	3.0891
110073441440	10692.05	441.9878	4.1338	333.3887	3.1181
110073320313	12256.79	457.4234	3.732	411.8158	3.3599
110073010170	12325.95	442.9824	3.5939	381.2047	3.0927

110073320322	10000.55	355.8096	3.5579	300.3266	3.0031
110073441329	8275.227	418.3127	5.055	249.0098	3.0091
110073011289	10587.85	406.9227	3.8433	314.6284	2.9716
110073440233	11459.71	401.2846	3.5017	404.1266	3.5265
110073011266	8408.066	300.1848	3.5702	253.4275	3.0141
110073011305	12536.76	524.1243	4.1807	365.6471	2.9166
110073441279	10536.8	383.6658	3.6412	328.1158	3.114
110073120081	9921.738	390.718	3.938	288.3654	2.9064
110073090169	12717.59	419.2862	3.2969	409.7225	3.2217
110073090167	8293.517	277.2108	3.3425	261.9507	3.1585
110073441255	10132.17	404.0812	3.9881	304.7961	3.0082
110016011224	12209.76	422.0548	3.4567	367.4771	3.0097
110016011011	12314.52	400.9855	3.2562	360.5815	2.9281
110016011237	12482.25	401.0547	3.213	378.8613	3.0352
110016011278	8784.94	263.7503	3.0023	279.4138	3.1806
110016010294	12644.06	425.8394	3.3679	356.1706	2.8169
110038011032	9723.74	321.7975	3.3094	279.6839	2.8763
110038120111	8691.265	334.2921	3.8463	265.9614	3.0601
110038120057	11583.04	437.0396	3.7731	357.302	3.0847
110038011019	11823.08	390.6465	3.3041	355.9221	3.0104
110038120066	12963.08	581.6924	4.4873	378.0683	2.9165
110038011284	14101.13	478.5923	3.394	411.4004	2.9175
110038120004	10329	352.3222	3.411	311.5846	3.0166
110038120021	14167.43	442.6045	3.1241	418.3782	2.9531
110038011215	11875.66	433.5448	3.6507	356.3174	3.0004
110038011261	11585.61	438.8976	3.7883	370.5541	3.1984
110038011295	9081.639	342.4414	3.7707	278.7609	3.0695
110038011267	9610.074	278.2789	2.8957	281.5944	2.9302
110038011220	13535.8	551.6107	4.0752	389.3301	2.8763
110038120047	8943.388	442.4652	4.9474	269.4106	3.0124
110038120064	12214.11	431.1948	3.5303	366.5699	3.0012
110044120817	8339.721	311.9639	3.7407	254.2447	3.0486
110038120028	11721.78	420.8355	3.5902	358.7335	3.0604
110038011210	10904.25	402.5304	3.6915	316.5722	2.9032
110038120014	12076.71	583.5223	4.8318	343.8239	2.847
110038010199	10412.42	320.4528	3.0776	305.6046	2.935
110038011183	9881.664	299.7998	3.0339	250.1247	2.5312
110038011034	11061.91	409.2464	3.6996	325.1869	2.9397
110038011297	6915.857	266.4956	3.8534	200.9333	2.9054
110038011263	12118.95	375.9055	3.1018	351.5827	2.9011
110038011191	13632.02	494.1471	3.6249	420.466	3.0844
110038011148	12794.19	420.0843	3.2834	380.2944	2.9724
110016011001	10532.7	393.986	3.7406	346.0833	3.2858
110016010278	12786.14	393.5189	3.0777	354.176	2.77

110016120007	11283.18	451.1467	3.9984	322.9246	2.862
110016011201	10278.91	338.1248	3.2895	302.642	2.9443
110016011014	6504.716	264.4752	4.0659	181.7483	2.7941
110016011002	8667.428	355.2952	4.0992	253.9123	2.9295
110016010240	10117	346.4567	3.4245	328.155	3.2436
110016010247	14006.66	504.1698	3.5995	427.1472	3.0496
110016010295	13301.28	393.5848	2.959	360.5577	2.7107
110016010290	12882.22	421.6996	3.2735	347.5624	2.698
110016011249	8801.032	276.8013	3.1451	268.5195	3.051
110016011251	4639.168	104.0473	2.2428	108.0601	2.3293
110016011256	10876.71	333.567	3.0668	339.7449	3.1236
110016011231	10738.51	295.0619	2.7477	303.1802	2.8233
110016011291	9155.432	391.1841	4.2727	295.6472	3.2292
110016010258	8100.168	307.2961	3.7937	247.4601	3.055
110045X11128	6846.765	285.3937	4.1683	228.3807	3.3356
110045010278	11514.79	259.9118	2.2572	367.4138	3.1908
110045011247	10864.28	388.3761	3.5748	322.8863	2.972
110045011266	13507.34	468.0969	3.4655	399.7093	2.9592
110045120025	11468.75	357.3892	3.1162	332.72	2.9011
110045011284	12064.71	333.9754	2.7682	371.9431	3.0829
110045010227	13546.23	393.1386	2.9022	396.3084	2.9256
110045011241	10894.41	395.0857	3.6265	352.5866	3.2364
110045011254	16512.08	489.4675	2.9643	467.0275	2.8284
110045010262	11696.84	341.8052	2.9222	346.1798	2.9596
110045120031	11591.82	363.4034	3.135	359.5318	3.1016
110045120048	12340.22	490.3139	3.9733	398.6507	3.2305
110045120030	9781.103	331.4816	3.389	264.0506	2.6996
110045011118	12586.04	406.3152	3.2283	384.4532	3.0546
110045120009	8588.034	296.1841	3.4488	280.5453	3.2667
110045X11253	6777.643	271.0244	3.9988	211.842	3.1256
110045011252	10405.61	337.9013	3.2473	296.7992	2.8523
110045011251	13298.29	481.3051	3.6193	396.1296	2.9788
110045120036	8916.572	255.656	2.8672	269.4053	3.0214
110045011244	11542.54	404.9699	3.5085	336.1533	2.9123
110045120063	9672.06	300.8011	3.11	319.236	3.3006
110045120099	12079.79	424.0246	3.5102	361.548	2.993
110032010296	12496.11	410.8596	3.2879	389.6912	3.1185
110032011031	12510.9	443.7741	3.5471	387.0997	3.0941
110032011267	12210.1	536.5607	4.3944	362.3958	2.968
110032011290	12216.06	466.6167	3.8197	326.2542	2.6707
110032011003	11559.9	462.1762	3.9981	353.2011	3.0554
110032012064	9580.347	506.4076	5.2859	249.204	2.6012
110032120092	10615.45	421.0725	3.9666	305.3428	2.8764
110032011281	10995.29	382.1742	3.4758	289.7478	2.6352

110032010323	12066.08	392.6787	3.2544	359.1349	2.9764
110032011301	9816.211	363.828	3.7064	282.1866	2.8747
110032011058	11718.54	356.6773	3.0437	295.0261	2.5176
110032011274	8882.647	436.7686	4.9171	260.8212	2.9363
110032011303	8016.954	387.8041	4.8373	242.8817	3.0296
110032120097	11573.09	326.0139	2.817	312.8321	2.7031
110032011262	13131.09	492.534	3.7509	382.6793	2.9143
110032012124	9143.497	365.7765	4.0004	220.3949	2.4104
110032012069	8694.907	451.4048	5.1916	268.7248	3.0906
110032011273	12599.55	487.6909	3.8707	346.6515	2.7513
110032012063	11933.51	466.6239	3.9102	330.9519	2.7733
110032012071	10220.8	403.3538	3.9464	299.0505	2.9259
110032012119	11192.14	375.4291	3.3544	325.6017	2.9092
110032011270	11836.07	442.7636	3.7408	360.4793	3.0456
110032120096	9456.24	422.1455	4.4642	271.2333	2.8683
110015011475	14300.96	623.8509	4.3623	440.6126	3.081
110015011676	11545.02	445.5222	3.859	385.3149	3.3375
110015011603	12460.58	523.5561	4.2017	386.1159	3.0987
110015011641	12146.85	444.4532	3.659	367.5272	3.0257
110015011700	13736.62	530.5771	3.8625	428.2805	3.1178
110015011528	9003.847	354.4814	3.937	289.3386	3.2135
110015011519	9603.68	396.3535	4.1271	301.2674	3.137
110015011548	14182.27	548.7121	3.869	412.1794	2.9063
110015120050	10476.92	388.7043	3.7101	324.9627	3.1017
110015011585	12524.67	575.1453	4.5921	410.709	3.2792
110015011681	7468.992	311.6437	4.1725	207.3467	2.7761
110015011652	11950.7	424.1781	3.5494	359.5965	3.009
110015120028	9059.596	334.0364	3.6871	311.7135	3.4407
110015011211	7355.632	288.6129	3.9237	232.9161	3.1665
110015120007	10763.95	389.3751	3.6174	341.9384	3.1767
110015011684	10464.11	357.2239	3.4138	290.7558	2.7786
110015120089	10939.46	398.8965	3.6464	293.3744	2.6818
110015011570	9075.473	399.4116	4.401	252.7791	2.7853
110015011387	12212.2	453.5245	3.7137	381.3382	3.1226
110015011582	14159.33	535.1379	3.7794	398.033	2.8111
110015011228	9747.217	388.5436	3.9862	321.2975	3.2963
110015011647	8738.15	402.8549	4.6103	307.7227	3.5216
110015011562	9865.008	365.755	3.7076	297.7358	3.0181
110015120070	9477.585	327.7823	3.4585	267.0309	2.8175
110015011537	13343.79	513.6557	3.8494	418.3544	3.1352
110015011534	11604.23	494.468	4.2611	381.6633	3.289
110015011563	11005.62	416.4966	3.7844	328.0885	2.9811
110015011614	12158.32	503.4759	4.141	362.4151	2.9808
110015011645	11786.96	432.4634	3.669	338.3092	2.8702

110015011705	7274.157	250.9875	3.4504	230.0525	3.1626
110015011685	8407.637	340.1141	4.0453	282.4209	3.3591
110015011500	12981.68	507.4278	3.9088	405.0543	3.1202
110015011595	8943.656	384.3805	4.2978	301.4549	3.3706
110015011649	10540.71	435.0677	4.1275	337.9667	3.2063
110015120012	11786.43	410.604	3.4837	389.3648	3.3035
110015011527	11554.21	475.6292	4.1165	343.7725	2.9753
110015011634	12137.17	429.3524	3.5375	342.2197	2.8196
110015011638	12210.16	475.6222	3.8953	395.0718	3.2356
110015011581	10172.71	414.192	4.0716	310.9085	3.0563
110015011701	10149.53	327.9109	3.2308	318.6443	3.1395
110015011547	11750.4	376.0715	3.2005	354.0982	3.0135
110015011550	14550.14	570.9328	3.9239	443.6482	3.0491
110015011698	10788.5	379.5718	3.5183	310.6225	2.8792
110015011589	11025.52	461.5173	4.1859	336.9179	3.0558
110015011670	13681.87	522.9211	3.822	397.3216	2.904
110015011192	12094.01	438.3474	3.6245	388.1088	3.2091
110015011554	13215.44	499.636	3.7807	398.9608	3.0189
110015011686	10699.15	373.9781	3.4954	341.0354	3.1875
110015011619	11429.81	453.3176	3.9661	349.0434	3.0538
110015011610	10545.9	483.8353	4.5879	326.944	3.1002
110015011503	10883.86	418.0598	3.8411	346.1393	3.1803
110015120045	11697.05	508.6812	4.3488	351.5314	3.0053
110015011661	9238.958	409.8956	4.4366	302.3172	3.2722
110015011542	9757.047	410.9961	4.2123	319.7384	3.277
110015011499	13038.71	468.4027	3.5924	402.3616	3.0859
110015011576	8818.981	322.4131	3.6559	282.516	3.2035
110015011605	10188.32	428.1436	4.2023	295.563	2.901
110015011639	10951.1	385.7965	3.5229	360.1818	3.289
110015011556	10906.54	440.0462	4.0347	350.0236	3.2093
110015011526	11620.49	484.2954	4.1676	361.1531	3.1079
110015011394	8917.335	377.0963	4.2288	238.1374	2.6705
110015011099	7126.126	303.972	4.2656	200.1087	2.8081
110015011569	9555.953	377.2404	3.9477	291.0839	3.0461
110015011693	13103.62	547.4953	4.1782	350.7969	2.6771
110015120030	13677.3	516.1403	3.7737	380.9265	2.7851
110015011704	9399.814	344.2306	3.6621	292.7572	3.1145
110015011513	12440.92	471.5232	3.7901	375.9645	3.022
110015011509	13290.52	503.6972	3.7899	375.5767	2.8259
110015011505	4756.938	180.4449	3.7933	152.4123	3.204
110015011571	9679.099	394.1039	4.0717	313.9803	3.2439

Table S4-5 Pedigree information of individuals used in this study.

ID	Sire	Dam	Grandsire	Granddam	Maternal Grandsire	Maternal Granddam
110046120121	11108672	110046009046	17131025	17388911	23206042	110046003117
110046120018	11101930	110046007227	2294436	123260594	11102919	110046020003
110046011145	11101930	110046007155	2294436	123260594	131601389	110046099106
110046120099	11108672	110046007113	17131025	17388911	11102919	110046005069
110046011040	11101916	110046001089	830287	128036514	11194165	110046099061
110046011147	11101930	110046009070	2294436	123260594	132483775	110046007013
110046120034	11101916	110046007208	830287	128036514	23105297	110046003113
110046011262	11101915	110046007239	2283503	17348883	11102919	110046001222
110046120028	11102412	110046009247	17083054	W00010712	11102129	110046007195
110046011253	11101917	110046006093	17099649	17394861	11102919	110046004035
110046011155	11101916	110046005082	830287	128036514	11196100	110046001021
110046120051	11101930	110046009273	2294436	123260594	11199995	110046002093
110046120024	11102412	110046009275	17083054	W00010712	101034031	110046007239
110046011179	11101916	110046008099	830287	128036514	11199090	110046005063
110046011068	11101916	110046004015	830287	128036514	11196018	110046099017
110046011267	11101915	110046006095	2283503	17348883	11101907	110046C98071
110046011279	11101915	110046008045	2283503	17348883	11101917	110046003015
110046011158	11101916	110046009037	830287	128036514	11199995	110046007010
110046120001	11101915	110046008240	2283503	17348883	11199090	110046004233
110046011259	11101915	110046008198	2283503	17348883	11100113	110046001214
110046011144	11102912	110046009117	17226843	125322098	132483775	110046007075
110046120006	11103863	110046009259	A00011306	X02N70045	11199095	110046006154
110046120023	11103863	110046009238	A00011306	X02N70045	11102129	110046005110
110046011178	11101917	110046007086	17099649	17394861	31204487	110046004148
110046011037	11101916	110046007004	830287	128036514	11101907	110046002119
110046120126	11108549	110046008142	123645630	17213746	13205121	110046003066
110046011153	11101916	110046008105	830287	128036514	11100113	110046097054
110046120103	11108672	110046008195	17131025	17388911	11100113	110046004114
110046011189	11101930	110046009093	2294436	123260594	132483775	110046007052
110046120027	11101930	110046009234	2294436	123260594	101034031	110046007168
110046011138	11102912	110046009101	17226843	125322098	132483775	110046007066
110046011265	11101915	110046009248	2283503	17348883	101034031	110046007220
110046120089	11101915	110046005045	2283503	17348883	11196100	110046001214
110046011263	11101915	110046009031	2283503	17348883	11199090	110046005013
110046011148	11101916	110046008120	830287	128036514	7653	110046006073
110046011198	11101916	110046005197	830287	128036514	11196100	110046001008
110045X11128	11108541	110010008078	123645630	17213746		
110045010227	11101930	110045008065	2294436	123260594	12105214	110045004001
110045011241	11101930	110045009121	2294436	123260594	7175748	110045002165
110045011254	11101930	110045008218	2294436	123260594	11102919	110045003202
110045010262	11108541	110045000131	123645630	17213746	11196100	110045098143

110045120031	11103863	110045009254	A00011306	X02N70045	11102919	110045005033
110045120048	11103863	110045010055	A00011306	X02N70045	61515275	110045008001
110045120030	11103863	110045010017	A00011306	X02N70045	11197314	110045008002
110045011118	11101930	110045009071	2294436	123260594	37306004	110045006276
110045120009	11101930	110045009007	2294436	123260594	11102919	110045005131
110045011270	11101930	110045008226	2294436	123260594	11102919	110045005146
110045X11253	11108541	110010009203	123645630	17213746	101034031	110010006749
110045120023	11102910	110045006283	17058140	124658053	11197314	110045004143
110045011252	11102919	110045007215	830287	120067165	31104496	110045005112
110045011251	11101930	110045008115	2294436	123260594	8264964	110045000011
110045011200	11102919	110045002159	830287	120067165	11196044	110045099152
110045120036	11101917	110045009225	17099649	17394861	23107073	110045005178
110045120053	11103863	110045010019	A00011306	X02N70045	295614274	110045007191
110045120021	11101929	110045009027	17056520	15972916	11102919	110045003227
110045011244	11102919	110045006217	830287	120067165	3758	110045004132
110045120063	11102910	110045008210	17058140	124658053	132483775	110045006215
110045011278	11101930	110045007006	2294436	123260594	11101906	110045002168
110045120099	11101917	110045005159	17099649	17394861	11197314	110045002077
110045010278	11108541	110045006133	123645630	17213746	11197314	110045020130
110045011247	11102919	110045002132	830287	120067165	11196044	110045D96092
110045011266	11102919	110045007067	830287	120067165	13204079	110045002205
110045120025	11102910	110045006066	17058140	124658053	11197314	110045002195
110045011284	11101930	110045009240	2294436	123260594	53207074	110045004041
110045011193	11102919	110045004179	830287	120067165	11196018	110045000041
110024011202	11103330	110024008209	A00011306	C00101542		
110024120036	11103330	110024008348	A00011306	C00101542		
110024011337	11103330	110024008323	A00011306	C00101542		
110024011336	11103330	110024005127	A00011306	C00101542		
110024011270	11104114	110024004062	17349617	129062082		
110024011126	11103330	110024006091	A00011306	C00101542		
110024011313	11103330	110024007163	A00011306	C00101542		
110024120030	11103863	110024009305	A00011306	X02N70045		
110024120004	11103330	110024008137	A00011306	C00101542		
110024011297	11103330	110024005112	A00011306	C00101542	11101927	110024002058
110024011258	11104114	110024006070	17349617	129062082		
110024120091	11104710	110024008208	98597	918564		
110024011333	11103330	110024007224	A00011306	C00101542		
110024011192	11103330	110024005200	A00011306	C00101542		
110024011253	11104114	110024007217	17349617	129062082		
110024011338	11104114	110024005250	17349617	129062082		
110024120125	11102129	110024009134	2149849	14142293		
110024011304	11102909	110024008199	2282997	60141550		
110024011201	11103330	110024007027	A00011306	C00101542		
110024011308	11103330	110024008322	A00011306	C00101542		

110024120032	11103330	110024006331	A00011306	C00101542		
110024011299	11102909	110024008007	2282997	60141550		
110024011315	11103330	110024007183	A00011306	C00101542		
110024011275	11104114	110024006161	17349617	129062082		
110024011300	11102412	110024009287	17083054	W00010712		
110024120005	11103330	110024006318	A00011306	C00101542		
110024120008	11103330	110024006525	A00011306	C00101542		
110024011343	11103330	110024009077	A00011306	C00101542		
110024011295	11102909	110024008025	2282997	60141550		
110024011028	11104114	110024008067	17349617	129062082		
110024011327	11104114	110024009031	17349617	129062082		
110024011335	11104114	110024008253	17349617	129062082		
110024010118	11102909	110024005138	2282997	60141550		
110024011263	11102412	110024009241	17083054	W00010712		
110024011339	11103330	110024008223	A00011306	C00101542		
110024011326	11104114	110024009031	17349617	129062082		
110024011283	11104114	110024005049	17349617	129062082		
110024011204	11103330	110024008068	A00011306	C00101542		
110024011322	11103330	110024008182	A00011306	C00101542		
110024120018	11104114	110024008188	17349617	129062082		
110022511428	11106002	110022109326	17062963	110505000771	11102909	110022105195
110022511520	11103825	110022105027	823672	H00931599	11100113	1100220M2409
110022110418	11103825	110023002163	823672	H00931599		
110022511431	11103825	110022107247	823672	H00931599	11101930	110022101199
110022512164	11105011	110022008329	17131025	17355619		
110022511446	11103863	110022109359	A00011306	X02N70045	11101905	110022105061
110022512003	11104940	110022105297	17013604	15543166	11196015	1100220M2673
110022511533	11108541	110022109301	123645630	17213746	11101927	110022107235
110022512014	11108541	110022109012	123645630	17213746	11102396	110022105263
110022512083	11105011	110022106213	17131025	17355619	11100113	110022104119
110022512138	11106002	110022008400	17062963	110505000771		
110022511408	11108541	110022108221	123645630	17213746	13204104	110022105054
110022511461	11103825	110022104361	823672	H00931599	11196044	1100220M2375
110022512007	11103825	110022105186	823672	H00931599	11102019	1100220M2566
110022110399	11106002	110022106024	17062963	110505000771	11102909	110025003160
110022512159	11106002	110022010051	17062963	110505000771		
110022512001	11106002	110022103349	17062963	110505000771	11196018	1100220M1865
110022512077	11103435	110022107269			131601389	110022103297
110022511424	11103435	110022107320			15105497	110022103270
110022512112	11108541	110022105116	123645630	17213746	11100260	1100220M2496
110022511522	11108541	110022101106	123645630	17213746	11197314	1100220M1493
110022511556	11103825	110022108506	823672	H00931599	11104123	110022105215
110022512118	11108541	110022107084	123645630	17213746		110024002149
110022511411	11103825	110022108207	823672	H00931599	11196043	110022105101

110022512158	11108541	110022009149	123645630	17213746		
110022511512	11103825	110022108201	823672	H00931599	11196043	110022105015
110022512163	11108541	110022008160	123645630	17213746		
110022512010	11103825	110022108043	823672	H00931599	11104123	110022201056
110022511555	11103825	110022108126	823672	H00931599	11104123	110022401198
110022512023	11104940	110022108462	17013604	15543166	11102909	110022106247
110022511405	11103825	110022105237	823672	H00931599	11101907	1100220M2615
110022512144	11108541	110026003126	123645630	17213746		
110022512177	11104940	110023002109	17013604	15543166		
110022511443	11104940	110022109371	17013604	15543166	11102396	110022104283
110022511495	11103435	110022107455			11196046	110022102203
110022511558	11104940	110022109400	17013604	15543166	11199090	110022105212
110022511481	11104123	110022107003	124003312	17278995	11102919	110023002128
110022511076	11106002	110022106289	17062963	110505000771	11102129	
110022511458	11108541	110022109315	123645630	17213746	11102129	110022105012
110022511539	11108541	110024003169	123645630	17213746		
110022512113	11105011	110022107306	17131025	17355619	131601389	110022501099
110022511425	11103825	110022105235	823672	H00931599	11102019	1100220M2613
110022511457	11104940	110022106141	17013604	15543166	11101929	110023001016
110022511307	11103825	110022106270	823672	H00931599	11101930	110022101067
110022511110	11104123	110022101240	124003312	17278995	11196044	1100220M1537
110022511552	11106002	110022107377	17062963	110505000771	11100260	110022199074
110022511355	11104123	110022106264	124003312	17278995		110022102200
110022511549	11104940	110022106217	17013604	15543166	11101906	110022103259
110022511500	11108541	110022109332	123645630	17213746	101034031	110022107252
110022511497	11108541	110023006011	123645630	17213746		
110022512093	11108541	110022108532	123645630	17213746	11101907	110022106298
110022511496	11108541	110022108408	123645630	17213746	132483775	110022106197
110022511536	11104123	110022103081	124003312	17278995	11194160	1100220M1715
110022512133	11106006	110022109176	2259250	110505000890	11102129	110022102237
110022512126	11108541	110022110075	123645630	17213746	60555239	110022108016
110022512108	11104940	110022106048	17013604	15543166		110022103352
110022511488	11104123	110022108140	124003312	17278995	11102129	110022103249
110022511448	11104123	110022102195	124003312	17278995	11193546	1100220M1628
110022511510	11103825	110022106097	823672	H00931599	11102390	110022102279
110022511482	11106002	110022106271	17062963	110505000771		110022103149
110022511547	11104940	110022102236	17013604	15543166	11194108	1100220M1650
110022511544	11104123	110022107425	124003312	17278995	11102909	110022101006
110022512073	11108541	110022108213	123645630	17213746	65105003	110022105085
110022512032	11105011	110022108483	17131025	17355619	11101907	110022403182
110022511494	11103435	110022108438			11102909	110022103105
110055120048	11105687	110055007134	11196529	2132	15105710	110055001183
110055011344	11101927	110005408172	2290977	124351844		
110055011269	11101927	110005407140	2290977	124351844		

110055011343	11101929	110055008175	17056520	15972916	11199090	110055004003
110055011323	11101927	110055006141	2290977	124351844	11196529	110055004021
110055011212	11101929	110055007098	17056520	15972916	131601389	110055004118
110055011316	11101927	110055005119	2290977	124351844	11196100	110055001251
110055120016	11104114	110055009005	17349617	129062082	101034031	110055006249
110055011308	11101929	110055608186	17056520	15972916		
110055011292	11101929	110055001258	17056520	15972916	11195006	110055097233
110055011284	11101929	110055708398	17056520	15972916		
110055011280	11101927	110055005152	2290977	124351844	11102919	110055001106
110055011325	11101927	110005408199	2290977	124351844		
110055011367	11104114	110005407127	17349617	129062082		
110055011390	11101929	110005407128	17056520	15972916		
110055011348	11104114	110055109221	17349617	129062082	23207008	110055106042
110055H12003	11102921	110055B10817	17099649	127368865		
110055011321	11104114	110005404214	17349617	129062082		
110055011386	11101929	110005405198	17056520	15972916		
110055011317	11104114	110055008158	17349617	129062082	11199090	110055003185
110055H11288	11102921	110055157483	17099649	127368865		
110055011303	11101927	110055008102	2290977	124351844	11199095	110055003040
110055011377	11101929	110055008106	17056520	15972916	132483775	110055006127
110055011330	11101929	110055008152	17056520	15972916	11199095	110055005163
110055H11272	11102921	110055007086	17099649	127368865	11101927	110055000110
110055120015	11101929	110055009198	17056520	15972916	11102919	110055007045
110055011267	11104114	110055908565	17349617	129062082		
110055011327	11101927	110005407137	2290977	124351844		
110055011268	11101915	110055009165	2283503	17348883	11199095	110055099027
110055011356	11104114	110005406104	17349617	129062082		
110055011310	11104114	110055009181	17349617	129062082	11199095	110055006132
110055011320	11104114	110055009209	17349617	129062082	11102919	110055004024
110055011339	11101927	110055608098	2290977	124351844	11104070	110055603248
110055011346	11101927	110005405196	2290977	124351844		
110055011351	11101927	110005405086	2290977	124351844		
110055011181	11101929	110055006215	17056520	15972916	31104422	110055002036
110055011301	11101929	110055608151	17056520	15972916		
110055011326	11101929	110055005135	17056520	15972916	11196100	110055000182
110055011328	11101927	110055009204	2290977	124351844	11102919	110055007063
110055011300	11104114	110055009175	17349617	129062082	11102919	110055005070
110055011359	11101929	110055908778	17056520	15972916		
110055H12008	11102921	110005519012	17099649	127368865		
110055011337	11101927	110055908363	2290977	124351844		
110055H11298	11106002	110055228381	17062963	110505000771		
110055011385	11101929	110005407151	17056520	15972916		
110055011354	11101929	110005405048	17056520	15972916		
110055011368	11101929	110005408195	17056520	15972916		

110055120002	11104114	110055009207	17349617	129062082	11102919	110055003044
110055011304	11104114	110055009215	17349617	129062082	11102919	110055006097
110026211175	11101906	110023008059	2297473	123884970		
110026211259	11101906	110023008116	2297473	123884970		
110032010296	11101906	110032002202	2297473	123884970	11196044	110032098055
110032011031	11102910	110032003176	17058140	124658053	11100113	110032001169
110032011268	11102909	110032008081	2282997	60141550		
110032011267	11103543	110032008205	17131025	17278995		
110032011290	11102010	110032005195	2245673	60294138	11100113	110032003130
110032011298	11102010	110032005253	2245673	60294138		
110032011003	11101906	110032005263	2297473	123884970		
110026211164	11102912	110026005249	17226843	125322098		
110032012064	11103543	110032007141	17131025	17278995		
110032120092	11102129	110032009053	2149849	14142293		
110032011260	11103543	110032007033	17131025	17278995		
110032011281	11102010	110032009303	2245673	60294138		
110032010323	11101906	110032004022	2297473	123884970	11196018	110056098358
110032011301	11103863	110032009310	A00011306	X02N70045		
110032011058	11102910	110032009059	17058140	124658053		
110032011274	11102909	110032008208	2282997	60141550		
110032011259	11102909	110032008232	2282997	60141550		
110032011303	11102909	110032009250	2282997	60141550		
110026211178	11101906	110026006143	2297473	123884970		
110032120097	11102129	110032009115	2149849	14142293		
110032011262	11101917	110032008198	17099649	17394861		
110032012124	11102912	110032010063	17226843	125322098		
110032012069	11101917	110032006131	17099649	17394861		
110032011273	11101917	110032007110	17099649	17394861		
110032012063	11103543	110032007141	17131025	17278995		
110032012071	11101917	110032008225	17099649	17394861		
110032012117	11102912	110032004211	17226843	125322098	11198748	110032002190
110032012099	11101917	110032004181	17099649	17394861	11196018	110032001158
110032012119	11101917	110032009034	17099649	17394861		
110026211250	11101906	110023008131	2297473	123884970		
110032011270	11101917	110032007245	17099649	17394861		
110032120096	11102129	110032009026	2149849	14142293		
110032011261	11101917	110032007145	17099649	17394861		
110032011282	11101930	110032005174	2294436	123260594	11100113	110032003082
110026210210	11102921	110026005033	17099649	127368865		
110026811296	11102910	110038009252	17058140	124658053	11103543	110038006215
110026120036	11102909	110023009180	2282997	60141550		
110026211271	11101906	110023009121	2297473	123884970		
110026211136	11102912	110026005232	17226843	125322098		
110026120011	11102909	110026006042	2282997	60141550		

110026210045	11101906	110023007296	2297473	123884970		
110026210088	11101906	110026005105	2297473	123884970		
110026120054	11102909	110023007283	2282997	60141550		
110026120079	11102921	110023008276	17099649	127368865		
110026211276	11102912	110026003191	17226843	125322098		
110026211137	11102909	110023008058	2282997	60141550		
110026120048	11102909	110023009012	2282997	60141550		
110026120039	11102909	110026004277	2282997	60141550	11194107	110023001111
110026211278	11102912	110023008137	17226843	125322098		
110026120057	11102921	110023007123	17099649	127368865		
110026120027	11102909	110023008243	2282997	60141550		
110026120005	11101906	110026207127	2297473	123884970	60270088	110026502037
110026211200	11101906	110023007234	2297473	123884970		
110026211238	11101906	110026004056	2297473	123884970		
110026211231	11101906	110025002158	2297473	123884970		
110026211192	11101906	110026006145	2297473	123884970		
110026120052	11102909	110023007208	2282997	60141550		
110026812009	11102912	110038007234	17226843	125322098	11199095	110038005046
110026120004	11102129	110026006298	2149849	14142293		
110026211236	11101906	110023009101	2297473	123884970		
110026120019	11102912	110023007231	17226843	125322098		
110026211253	11101906	110026005208	2297473	123884970		
110026120020	11102912	110023008241	17226843	125322098		
110026211268	11102412	110023009279	17083054	W00010712		
110026211275	11102129	110026006189	2149849	14142293		
110026210051	11101930	110023008014	2294436	123260594		
110026120024	11102909	110023009026	2282997	60141550		
110026211004	11102129	110026005216	2149849	14142293		
110026120017	11102912	110023008266	17226843	125322098		
110026120044	11102921	110026004246	17099649	127368865		
110026211285	11102129	110025002230	2149849	14142293		
110026209249	11101906	110026005119	2297473	123884970		
110026211239	11102129	110023008076	2149849	14142293		
110026211289	11102129	110023007212	2149849	14142293		
110026211277	11102129	110023007218	2149849	14142293		
110026210219	11101906	110026006256	2297473	123884970		
110026211233	11102129	110026006210	2149849	14142293		
110026811208	11102910	110038007036	17058140	124658053	11199095	110038002031
110026120007	11102909	110023008231	2282997	60141550		
110026120055	11102921	110026006060	17099649	127368865		
110026211142	11102909	110023006298	2282997	60141550		
110026120013	11102129	110023007260	2149849	14142293		
110026120028	11102912	110023009188	17226843	125322098		
110026211251	11102412	110023009141	17083054	W00010712		

110026210206	11101906	110023007137	2297473	123884970		
110026211230	11101906	110023008193	2297473	123884970		
110026211258	11101906	110026004216	2297473	123884970	11100113	110026002086
110033012066	11101917	110033005018	17099649	17394861	11196015	110033000040
110033010067	11100260	110033003055	2149849	14142293	11196046	110033098372
110033011239	11105007	110033006040	17131025	17355619	11101907	110033304015
110033011030	11105007	110033006133	17131025	17355619	31104442	110033304036
110033011232	11101917	110033004180	17099649	17394861		
110033011235	11105007	110033007165	17131025	17355619	11196100	110033305110
110033012006	11103863	110033009270	A00011306	X02N70045	11196035	110033306039
110033011225	11103863	110033009179	A00011306	X02N70045	11103561	110033306173
110033012034	11100260	110033007212	2149849	14142293	11100260	110033303013
110033011228	11105007	110033008253	17131025	17355619	11196100	110033304014
110033011237	11105007	110033007013	17131025	17355619	11101907	110033302101
110033011234	11103411	110033007009	830287	Q01279803	11100260	110033303135
110033011230	11105007	110033008220	17131025	17355619	11197314	110033306078
110033011236	11103411	110033009235	830287	Q01279803	11103519	110033306177
110033012021	11101917	110033008001	17099649	17394861	11197314	110033302087
110033012018	11101917	110033003067	17099649	17394861		
110033012009	11101927	110033005096	2290977	124351844	11101927	110033301020
110033012011	11108672	110033009176	17131025	17388911	11197314	110033306007
110033011081	11101917	110033007043	17099649	17394861	11100260	110033305045
110033011229	11105007	110033006180	17131025	17355619	31104427	110033300080
110033012043	11101917	110033005174	17099649	17394861	11196100	110033202170
110033011242	11105007	110032006175	17131025	17355619		
110033011220	11103411	110033007149	830287	Q01279803	11102912	110033303088
110033011219	11103863	110033009199	A00011306	X02N70045	11196100	110033306142
110033011256	11101917	110033008151	17099649	17394861	11103519	110033304084
110033011248	11103411	110033008158	830287	Q01279803	63105012	110033305107
110033012023	11108672	110033005169	17131025	17388911	11196015	110033303084
110033011053	11105007	110033004105	17131025	17355619		
110033012024	11108672	110033008048	17131025	17388911	11100519	110033305074
110033011231	11103411	110033005025	830287	Q01279803		
110033011200	11103863	110033009212	A00011306	X02N70045	11100260	110033304030
110033011145	11103411	110033002172	830287	Q01279803		
110033012025	11108672	110033006104	17131025	17388911	11199842	110033303060
110033012058	11101917	110033009258	17099649	17394861	11100260	110033301177
110033011241	11105007	110033006014	17131025	17355619	11102396	110033397032
110033011188	11103863	110033009186	A00011306	X02N70045	132483775	110033306061
110033011080	11101907	110033006114	2290977	15963036	11102912	110033303031
110033011086	11108672	110033007045	17131025	17388911	11101907	110033301050
110050011271	11101906	110050007048	2297473	123884970	31104461	110050003288
110050011291	11101906	110050007314	2297473	123884970	131601389	110050001314
110050011201	11102412	110050009163	17083054	W00010712	101034031	110050007290

110050011215	11102412	110050009179	17083054	W00010712	101034031	110050007396
110050011250	11101930	110050006170	2294436	123260594	11100113	110050002442
110050011302	11101927	110050007546	2290977	124351844	131601389	110022003074
110050011312	11101927	110050008348	2290977	124351844	11105013	110050005312
110050011288	11101906	110050009148	2297473	123884970	11101927	110050007549
110050011258	11101930	110050008180	2294436	123260594	9313518	110050006126
110050011253	11101906	110050009090	2297473	123884970	9255161	110050005034
110050011315	11101927	110050009165	2290977	124351844	11100113	110050001234
110050011307	11101927	110050005278	2290977	124351844	11193544	110050002036
110050011249	11101930	110050002264	2294436	123260594	11194127	110050096040
110050011276	11103435	110050008398			9294161	110050006290
110050011224	11102412	110050009079	17083054	W00010712	11102919	110050005490
110050N11162	11101906	110050008175	2297473	123884970		
110050120041	11101927	110050009152	2290977	124351844	11100113	110050005240
110050011275	11101930	110050005354	2294436	123260594	31103413	110050002242
110050011263	11101906	110050008333	2297473	123884970	11199090	110050005288
110050011193	11102412	110050009112	17083054	W00010712	11101916	110050004046
110050011284	11101930	110050008372	2294436	123260594	11100113	110050001452
110050120010	11101916	110050004332	830287	128036514	11194108	110050002104
110050011310	11105011	110050103028	17131025	17355619		
110050011252	11101916	110050006352	830287	128036514	11100113	110050004244
110050011277	11101930	110050004194	2294436	123260594	11194108	110050002106
110050011194	11102412	110050009159	17083054	W00010712	11101916	110050007255
110050N11132	11103543	110050007012	17131025	17278995	11100113	110050002264
110050120001	11101906	110050006476	2297473	123884970	11196018	110050002296
110050011261	11101906	110050005426	2297473	123884970	11100113	110050003112
110050011325	11101927	110050008407	2290977	124351844	11101914	110050004258
110050120043	11101927	110050009012	2290977	124351844	11101914	110050099440
110050011257	11101906	110050005150	2297473	123884970	11193544	110050002386
110050011232	11102412	110050009166	17083054	W00010712	11101916	110050007280
110050011327	11101927	110050007568	2290977	124351844		110022002164
110050011297	11103435	110050107188				
110050011301	11101906	110050004268	2297473	123884970	11199095	110050001162
110050011326	11101927	110050004196	2290977	124351844	11199095	110050001430
110050011286	11101906	110050008194	2297473	123884970	9255254	110050098414
110050011320	11101930	110050007539	2294436	123260594		110023001019
110050120007	11101906	110050007572	2297473	123884970		110022001166
110073011313	11102921	110073168151	17099649	127368865	9255051	110073005029
110073440155	11104070	110073008330	17131025	17388911		
110073120010	11106002	110073227405	17062963	110505000771		
110073011099	11102129	110073005027	2149849	14142293	11194127	110073003028
110073011316	11102921	110073005058	17099649	127368865	21102011	11007300A211
110073010220	11102921	110073007025	17099649	127368865	11197580	110073004102
110073011003	11104070	202158095	17131025	17388911		

110073120002	11102921	110073169034	17099649	127368865	11101906	110073005133
110073011246	11102921	110073448104	17099649	127368865	11100519	110073006069
110073011312	11102921	110073006064	17099649	127368865	11196035	110073000310
110073120012	11104940	110073226080	17013604	15543166		110022002239
110073011281	11102921	110073006125	17099649	127368865	11101927	110073002236
110073011200	11103863	110016009230	A00011306	X02N70045	132295034	110016002047
110073441440	11103863	110073009273	A00011306	X02N70045		
110073011314	11104940	110073225311	17013604	15543166		110022023148
110073320313	11104710	110032008012	98597	918564		
110073441278	11101930	110073009441	2294436	123260594		
110073011301	11102921	110073128270	17099649	127368865	11102396	110073005191
110073010170	11104114	110073006024	17349617	129062082	11195008	110073002025
110073320322	11104710	110032006076	98597	918564		
110073011303	11102921	110073007066	17099649	127368865	31105145	110073004046
110073011215	11106002	110073228397	17062963	110505000771		110022001106
110073011287	11102921	110073007022	17099649	127368865	11196044	110073000215
110073441329	11101906	110073003182	2297473	123884970		
110073011289	11102921	110073228456	17099649	127368865	11102129	110073202108
110073440233	11104710	110073008358	98597	918564		
110073011266	11102921	110073447308	17099649	127368865	11197314	110073005141
110073011305	11102921	110073080683	17099649	127368865	11194127	110073001936
110073441279	11104940	110073224283	17013604	15543166		110025002108
110073441460	11101906	110073007053	2297473	123884970	9043859	110073001856
110073120081	11105011	110073222247	17131025	17355619		110022095233
110073090169	11104123	110073008250	124003312	17278995		
110073090167	11104123	110073005225	124003312	17278995		
110073441255	11102129	110073009145	2149849	14142293	11102909	110073006015
110019011476	11103519	110019006146	17058140	15831061	11101902	110019H94076
110019011525	11103519	110019007104	17058140	15831061	32204486	110019005030
110019011550	11104070	110019009453	17131025	17388911	11101929	110019002384
110019011526	11103519	110019004209	17058140	15831061	11101929	110019000189
110019120091	11103519	110019010057	17058140	15831061	11108572	110019005267
110019011461	11104070	110019009246	17131025	17388911	9255161	110019007094
110019011478	11104070	110019006001	17131025	17388911		110013001042
110019011370	11104070	110019008344	17131025	17388911	10569537	110019006145
110019011450	11101930	110019007211	2294436	123260594	11102921	110019004260
110019011554	11103863	110019009549	A00011306	X02N70045	11102921	110019006344
110019011605	11104070	110019006295	17131025	17388911	11103561	110019X97056
110019011290	11101930	110019008168	2294436	123260594	129032447	1100190T2049
110019011574	11104070	110019006338	17131025	17388911	11102912	110019000286
110019011498	11104070	110019009452	17131025	17388911	11101930	110019006041
110019120149	11108549	110019009129	123645630	17213746	11101927	110019007077
110019011508	11104070	110019001386	17131025	17388911	11196015	110019098140
110019011485	11103519	110019005086	17058140	15831061	11199095	110019001335

110019011427	11103519	110019001385	17058140	15831061	11196015	110019042290
110019120114	11108549	110019009544	123645630	17213746	11197314	110019007496
110019011488	11103519	110019005284	17058140	15831061	11102019	110019001321
110019011462	11104070	110019008412	17131025	17388911	11195008	110019002089
110019011546	11101930	110019009491	2294436	123260594	64107308	110019007293
110019120126	11108672	110019009179	17131025	17388911	11103519	110019005153
110019011472	11103519	110019008094	17058140	15831061	11195020	110019005300
110019011497	11104070	110019008108	17131025	17388911	11101929	110019004045
110019011072	11104114	110019008214	17349617	129062082	11199995	110019004055
110019011492	11104070	110019007185	17131025	17388911	11102912	110019080091
110019011369	11103519	110019080511	17058140	15831061		
110019011487	11104070	110019009472	17131025	17388911	11197314	110019007402
110019010049	11103519	110019080048	17058140	15831061		
110019011446	11101930	110019006152	2294436	123260594	11101902	110019000Z82
110019011467	11104070	110019005150	17131025	17388911		1019002100
110019011522	11103863	110019009496	A00011306	X02N70045	6120	110019004009
110019120003	11104070	110019008170	17131025	17388911	11103519	110019005074
110019011314	11101930	110019007369	2294436	123260594	11195008	110019080418
110019011576	11104070	110019008365	17131025	17388911	9294161	110019006194
110019011475	11104070	110019008161	17131025	17388911	11103519	110019006088
110019120049	11109571	110019009071	60372887	134482565	11195008	110019006286
110019011473	11103519	110019080372	17058140	15831061		
110019011469	11104070	110019080078	17131025	17388911		
110019011381	11104070	110019008313	17131025	17388911	11105013	110019004120
110019011371	11104070	110019008103	17131025	17388911	11101929	110019004070
110019011490	11101930	110019080175	2294436	123260594		
110019011227	11104070	110019006400	17131025	17388911	11101907	110019004229
110019120171	11108672	110019080131	17131025	17388911		
110019011577	11103519	110019008329	17058140	15831061	11101929	110019080279
110015011475	11105007	110015009433	17131025	17355619	11104701	110015003754
110015011513	11105007	110015009539	17131025	17355619	11101902	110015005542
110015011509	11105007	110015009487	17131025	17355619	101034031	110015007435
110015120073	11104710	110015005565	98597	918564	11197580	110015003536
110015011505	11104710	110015004880	98597	918564	18015268	110015982745
110015011571	11104710	110015007470	98597	918564	11101908	110015005859
110015011586	11104114	110015008470	17349617	129062082	11102129	110015003496
110015011667	11104070	110015004395	17131025	17388911	128460878	1073789
110015011560	11104710	110015008610	98597	918564	11101929	110015005690
110015011548	11104114	110015005536	17349617	129062082	11101930	110015001126
110015011533	11105007	110015009511	17131025	17355619	15106120	110015003442
110015120050	11106006	110015009695	2259250	110505000890	11103560	110015007612
110015011585	11105007	110015009637	17131025	17355619	11101902	110015005756
110015011681	11104070	110015004834	17131025	17388911		110141141703
110015011598	11106006	110015005695	2259250	110505000890	11101930	110015003607

110015011652	11104070	110015005423				
110015120028	11104710	110015005823	98597	918564		1023570
110015011211	11104710	110015008192	98597	918564	11104701	110015005776
110015120007	11104070	110015007109	17131025	17388911	11102687	110015004853
110015011676	11104070	110015009614	17131025	17388911	11101929	110015004435
110015011684	11106006	110015005655	2259250	110505000890	11101902	110015000097
110015120089	11109571	110015007396	60372887	134482565	11199995	110015003428
110015011570	11104710	110015009322	98597	918564	11101929	110015005324
110015011493	11104710	110015005631	98597	918564	11199095	110015000074
110015011387	11104070	110015007153	17131025	17388911	11104701	110015003532
110015011582	11105007	110015009654	17131025	17355619	11101902	110015006667
110015011669	11104070	110015003288				
110015120022	11104070	110015003291	17131025	17388911		W98003476
110015011228	11104710	110015009198	98597	918564	11102912	110015007181
110015011603	11106006	110015009110				
110015011647	11104070	110015008493	17131025	17388911	11102129	110015005304
110015011562	11104114	110015009304	17349617	129062082	11101929	110015004849
110015120070	11104710	110015006683	98597	918564	11101902	110015000128
110015011537	11105007	110015009503	17131025	17355619	11102909	110015002628
110015011534	11104710	110015006740	98597	918564	11196100	110015099074
110015011563	11104710	110015008413				
110015011592	11105007	110015009642	17131025	17355619	11101902	110015004697
110015011614	11106006	110015009368	2259250	110505000890	11102017	110015004512
110015011645	11104070	110015005668				
110015011685	11104070	110015004337	17131025	17388911	2287161	110015115267
110015011500	11105007	110015009383	17131025	17355619	11101929	110015004449
110015011530	11104710	110015009351	98597	918564	11101929	110015005399
110015011595	11104114	110015006558	17349617	129062082	11101930	110015001111
110015011649	11104114	110015005356				
110015120012	11104070	110015009299	17131025	17388911	11101929	110015005370
110015011641	11104070	110015008600	17131025	17388911	11197580	110015006625
110015011527	11105007	110015009449	17131025	17355619	11102129	110015003448
110015011634	11105007	110015010050	17131025	17355619	11104070	110015007600
110015011638	11104114	110015005402	17349617	129062082		110161161127
110015011581	11105007	110015009525	17131025	17355619	11101902	110015003306
110015011701	11104070	110015005543	17131025	17388911	31102005	110015001193
110015010357	11106006	110015008306	2259250	110505000890	11101907	110015004910
110015011547	11104114	110015009415	17349617	129062082	11101929	110015004322
110015011550	11104070	110015008269	17131025	17388911	11101929	110015005803
110015011700	11104070	110015009676	17131025	17388911	11101929	110015004680
110015011267	11104710	110015009186	98597	918564	11101929	110015005486
110015011339	11104070	110015003316	17131025	17388911		
110015011698	11104070	110015009474	17131025	17388911	23107077	110015003263
110015011589	11104114	110015004348	17349617	129062082	163863148	110015414136

110015011670	11104070	110015003288	17131025	17388911		110015000000
110015011192	11106006	110015003417	2259250	110505000890		110020023757
110015011554	11104710	110015008329	98597	918564	9294161	110015005633
110015011686	11106006	110015004627	2259250	110505000890	11199815	110015002526
110015011528	11104710	110015008368	98597	918564	8264964	110015005847
110015120015	11104070	110015008323				
110015011351	11103863	110015009384	A00011306	X02N70045	11101929	110015006580
110015011619	11104114	110015009314				
110015011610	11104114	110015008591	17349617	129062082	11101929	110015005361
110015011503	11104710	110015009220	98597	918564	11102912	110015007173
110015120045	11106006	110015009650	2259250	110505000890	831070	110015007465
110015011661	11104070	110015008623	17131025	17388911	11101902	110015099204
110015120080	11106006	110015009674				
110015011696	11104070	110015008399	17131025	17388911	11102017	110015005629
110015011658	11104070	110015004573				
110015011519	11104710	110015005439	98597	918564	1210762	110196196278
110015011542	11104070	110015007598	17131025	17388911	11197314	110015005703
110015011476	11104710	110015007203	98597	918564	11101905	110015004904
110015011499	11104114	110015008248	17349617	129062082	11190611	110015004805
110015011593	11104070	110015009486	17131025	17388911	23206095	110015004400
110015011576	11104114	110015008594	17349617	129062082	11197580	110015002714
110015011605	11104114	110015004865	17349617	129062082		614987
110015011639	11104070	110015006765	17131025	17388911	11101907	110015004635
110015120048	11106006	110015009686	2259250	110505000890	11101929	110015001082
110015011556	11104710	110015007342	98597	918564	11101905	110015005468
110015011526	11105007	110015009632				
110015011622	11106006	110015009399				
110015011394	11106006	110015004719	2259250	110505000890	7175748	110015002709
110015011099	11106006	110015009022	2259250	110505000890	11101902	110015002775
110015011511	11104710	110015008441	98597	918564	42106547	110015003284
110015011569	11104710	110015005836	98597	918564	5279989	110215821534
110015011693	11103863	110015010003	A00011306	X02N70045	11105687	110015003328
110015120030	11104070	110015010040	17131025	17388911	129901651	110015007427
110015011642	11104070	110015008491	17131025	17388911	9255254	110015006599
110015011704	11104070	110015005304	17131025	17388911		110162816264
110016011001	11101906	110016006228	2297473	123884970	11196100	110016001264
110016010278	11101906	110016002069	2297473	123884970	11184919	110016099035
110016120007	11104859	110016009085	11189205	605	18058022	110016007030
110016011271	11101906	110016006178	2297473	123884970	11196100	110016001187
110016011201	11100260	110016006250	2149849	14142293	10569537	110016004169
110016011014	11104701	110016008236	163863148	962089	11102925	110016003185
110016011193	11100260	110016005246	2149849	14142293	11101930	110016003158
110016010247	11101906	110016005234	2297473	123884970	11101930	110016003109
110016010295	11100260	110016005197	2149849	14142293	13103025	110016003587

110016010290	11100260	110016005087	2149849	14142293	11101930	110016097069
110016011249	11104701	110016008299	163863148	962089	18058022	110016003160
110016011251	11104701	110016006191	163863148	962089	11196100	110016003136
110016011256	11101906	110016005003	2297473	123884970	11101929	110016001113
110016011234	11100260	110016007070	2149849	14142293	11101927	110016001171
110016011231	11104070	110016008247	17131025	17388911	11101915	110016002169
110016011291	11104701	110016002169	163863148	962089	11193539	110016092099
110016011247	11104701	110016007138	163863148	962089	11196046	110016003207
110016010258	11101915	110016004070	2283503	17348883	11199069	110016001057
110016011257	11104701	110016003273	163863148	962089	11198391	110016098116
110016011224	11101906	110016008221	2297473	123884970	18058022	110016006127
110016011011	11100260	110016007002	2149849	14142293	11196100	110016002136
110016011289	11103863	110016009316	A00011306	X02N70045	11101915	110016003281
110016011281	11103863	110016009309	A00011306	X02N70045	63105008	110016006217
110016011242	11100260	110016009158	2149849	14142293	11101906	110016001242
110016011237	11100260	110016008042	2149849	14142293	13205118	110016003213
110016011278	11103863	110016009196	A00011306	X02N70045	11101906	110016005039
110016010294	11100260	110016004089	2149849	14142293	11101914	110016099114
110017T11107	11102910	110017009118	17058140	124658053		
110017R11357	11101930	110017003282	2294436	123260594		
110017T11326	11199995	110017009042	2167531	15088752		
110017F11679	11102129	8050	2149849	14142293		
110017K11239	11103519	110017007201	17058140	15831061		
110017K11174	11103519	110017007099	17058140	15831061		
110017F12028	11102129	110017005752	2149849	14142293		
110017T12046	11104859	1101206130	11189205	605		
110017U11433	11104940	110017108237	17013604	15543166		
110017L11301	11104859	110017002069	11189205	605		
110017X11165	11101930	110017006052	2294436	123260594		
110017T11329	11104859	110012009011	11189205	605	11101927	110012006224
110017T11200	11102412	9261	17083054	W00010712		
110017J11601	11106006	110017009365	2259250	110505000890		
110017F11397	11102909	1105709162	2282997	60141550		
110017X11239	11101930	110017009228	2294436	123260594		
110017T12054	11104859	1101209084	11189205	605		
110017D11217	11102129	110017107161	2149849	14142293		
110017T11020	11101929	205129	17056520	15972916		
110017F11559	11101906	110017005667	2297473	123884970		
110017T11375	11102010	110017009085	2245673	60294138		
110017T11407	11102010	110017007441	2245673	60294138		
110017K12133	11108549	110017009581	123645630	17213746		
110017K11294	11101929	110017007517	17056520	15972916		
110017T11378	11102010	110017008018	2245673	60294138		
110017T11459	11102010	110017009007	2245673	60294138		

110017D11267	11101930	110017009020	2294436	123260594		
110017K11436	11104070	110017008243	17131025	17388911		
110017K11399	11103519	110017008385	17058140	15831061		
110017F11351	11102129	110017006042	2149849	14142293		
110017R11479	11199995	110017008270	2167531	15088752		
110017T12207	11102010	110017008460	2245673	60294138		
110017F11013	11101906	409005789	2297473	123884970		
110017T11400	11102010	110017009295	2245673	60294138		
110017V11110	11102910	110017008038	17058140	124658053		
110017J12091	11106006	110017006669	2259250	110505000890		
110017K11463	11101930	110017007135	2294436	123260594		
110017H11274	11102921	110017008131	17099649	127368865		
110017T12017	11108672	110017005034	17131025	17388911		
110017T11272	11101929	110017006207	17056520	15972916		
110017H10286	11101906	110017004076	2297473	123884970		
110017X11219	11101930	110017005024	2294436	123260594		
110017710030	11199995	110017A05482	2167531	15088752		
110017F11087	11101906	409005651	2297473	123884970		
110017U12079	11106002	110017108374	17062963	110505000771		
110017N12121	11102912	110017009234	17226843	125322098		
110017V11119	11102910	110017007082	17058140	124658053		
110017T12001	11199995	110017004191	2167531	15088752		
110017T11120	11102910	6009	17058140	124658053		
110017H11037	11104123	110017007326	124003312	17278995		
110017T11271	11104859	110017006199	11189205	605		
110017T11116	11102910	110017009103	17058140	124658053		
110017T11247	11103543	110017010027	17131025	17278995		
110017T11379	11102010	110017004541	2245673	60294138		
110017R12086	11100260	110017010080	2149849	14142293		
110037011419	11101930	110037007353	2294436	123260594	11196529	110037201022
110037120194	11101917	110037009250	17099649	17394861	11101927	110037007243
110037120102	11100260	110037010041	2149849	14142293	11103560	110037B20094
110037120105	11101917	110037009093	17099649	17394861	11101915	110037004317
110037011496	11101917	110037007364	17099649	17394861	11196529	110037003239
110037011509	11199995	110037007287	2167531	15088752	11102912	110037B20016
110037011373	11100260	110037009274	2149849	14142293	11101929	110037007167
110037011401	11100260	110037009386	2149849	14142293	11103560	110037004224
110037120061	11101917	110037008455	17099649	17394861	11196529	110037005189
110037011433	11101917	110037007349	17099649	17394861	11196100	110037005091
110037011404	11100260	110037007221	2149849	14142293	11101907	110037004240
110037120023	11199995	110037001038	2167531	15088752	11193558	11003700M427
110037011517	11100260	110037010022	2149849	14142293	129032447	110037007396
110037011456	11101917	110037008396	17099649	17394861	9313518	110037006290
110037011495	11101917	110037008229	17099649	17394861	11101915	110037005087

110037120073	11100260	110037010060	2149849	14142293	11103560	110037004236
110037011407	11101917	110037008072	17099649	17394861	11104738	110037004292
110037011497	11101917	110037009376	17099649	17394861	11104710	110037005287
110037011100	11103863	110037009091	A00011306	X02N70045	11101927	110037007092
110037011421	11101917	110037096066	17099649	17394861		
110037011476	11199995	110037006364	2167531	15088752	11102910	110037003130
110037011267	11101930	110037007357	2294436	123260594	9313518	110037005210
110037120056	11100260	110037010078	2149849	14142293	61515275	110037008076
110037010339	11102909	110037007242	2282997	60141550	60270088	110037002166
110037011492	11100260	110037009411	2149849	14142293	11199095	110037004238
110037011416	11101930	110037007372	2294436	123260594	11196529	110037005077
110037011487	11199995	110037008203	2167531	15088752	9294161	110037002187
110037011500	11101917	110037005156	17099649	17394861	11100519	110037B20046
110037011486	11199995	110037008231	2167531	15088752	11101915	110037202068
110037011431	11101917	110037008298	17099649	17394861	13205123	110037003122
110037011518	11100260	110037010008	2149849	14142293	11104738	110037007368
110037010326	11101916	110037008368	830287	128036514	11103560	110037004151
110037120026	11103863	110037010053	A00011306	X02N70045	11104903	110037008081
110037120032	11103863	110037010035	A00011306	X02N70045	11199095	110037001142
110037011491	11101917	110037008244	17099649	17394861	11101915	110037004333
110037120036	11100260	110037009394	2149849	14142293	129032447	110037007365
110037011434	11101930	110037007217	2294436	123260594	11102910	110037004050
110037011331	11199995	110037007212	2167531	15088752	11196035	110037203010
110037120096	11100260	110037010047	2149849	14142293	11103543	110037005001
110037011305	11199995	110037007381	2167531	15088752	11196035	110037201107
110037120093	11100260	110037010084	2149849	14142293	6860836	110037008053
110037011481	11199995	110037003077	2167531	15088752		
110037120033	11101917	110037009055	17099649	17394861	11101915	110037004164
110037120126	11100260	110037010096	2149849	14142293	6860836	110037006362
110037120066	11101917	110037008218	17099649	17394861	11101915	110037005081
110037011447	11199995	110037009275	2167531	15088752	11103560	110037006237
110037120078	11100260	110037010066	2149849	14142293	11107671	110037005263
110037011463	11100260	110037009414	2149849	14142293	11101915	110037003112
110037011022	11101927	110037007308	2290977	124351844	23105291	110037005233
110037011358	11101930	110037006251	2294436	123260594	32104266	110037201022
110037120020	11101917	110037003294	17099649	17394861	11100113	110037096063
110037120050	11101917	110037007218	17099649	17394861	11196035	110037004014
110037011453	11101917	110037007228	17099649	17394861	11101929	110037004359
110037011508	11100260	110037010016	2149849	14142293	11199095	110037B20014
110037011526	11103863	110037010013	A00011306	X02N70045	129032447	110037008007
110037011393	11199995	110037008310	2167531	15088752	11105013	110037001080
110037120052	11101917	110037009006	17099649	17394861	11103560	110037004229
110037011398	11101930	110037007331	2294436	123260594	9313518	110037005229
110037011505	11101917	110037006358	17099649	17394861	11102910	110037003132

110037011435	11101917	110037006169	17099649	17394861	31104431	110037B10093
110037120067	11101917	110037009012	17099649	17394861	11199095	110037005236
110037011399	11101917	110037008405	17099649	17394861	11199095	110037005299
110037011514	11103863	110037010038	A00011306	X02N70045	11103543	110037006101
110037120069	11101917	110037009021	17099649	17394861	11101915	110037005301
110037011446	11101917	110037008258	17099649	17394861	11101915	110037005095
110037011484	11101917	110037007365	17099649	17394861	9313518	110037005271
110037011450	11101917	110037004067	17099649	17394861	11100519	110037096089
110037011442	11101917	110037008341	17099649	17394861	11101915	110037004295
110037011469	11199995	110037008015	2167531	15088752	11199095	110037002143
110037011437	11199995	110037005287	2167531	15088752	11101927	110037003133
110037011411	11101917	110037008306	17099649	17394861	132483775	110037006155
110037120041	11103863	110037010005	A00011306	X02N70045	11104738	110037007057
110037011309	11101917	110037008163	17099649	17394861	11199095	110037001193
110037011451	11101917	110037008453	17099649	17394861	11103543	110037005267
110037011485	11101917	110037008044	17099649	17394861	9313518	110037005316
110037011420	11101917	110037008319	17099649	17394861	11103560	110037009961
110037120156	11101917	110037007313	17099649	17394861	15104150	110037005118
110037011430	11199995	110037005304	2167531	15088752	11101927	110037009954
110037011502	11101917	110037008156	17099649	17394861	9255051	110037006018
110037120054	11100260	110037010094	2149849	14142293	11101915	110037004281
110038120047	11102912	110038008266	17226843	125322098	8264964	110038006138
110038011175	11102412	110038009185	17083054	W00010712	11103543	110038006097
110038120064	11102910	110038009098	17058140	124658053	11101927	110038007039
110038120028	11102912	110038008277	17226843	125322098	11100113	110038004071
110038011210	11102912	110038007028	17226843	125322098	61204030	110038003313
110038120014	11102912	110038008029	17226843	125322098	32204003	110038003056
110038010199	11102910	110038004180	17058140	124658053	11196018	11003800M926
110038011307	11102910	110038006010	17058140	124658053	11101907	110038003805
110038011183	11102910	110038006029	17058140	124658053	23103238	110038013012
110038011034	11102910	110038007209	17058140	124658053	34304003	110038001185
110038011297	11102910	110038006214	17058140	124658053	23104263	110038201070
110038120070	11103863	110038010030	A00011306	X02N70045	11102912	110038008014
110038120089	11102910	110038010027	17058140	124658053	11102912	110038006249
110038011263	11102910	110038006242	17058140	124658053	11199095	110038002128
110038011191	11102910	110038009110	17058140	124658053	11199095	110038020145
110038011148	11102412	110038009169	17083054	W00010712	129032447	110038005027
110038120117	11102912	110038008178	17226843	125322098	11199095	110038003270
110038011270	11102912	110038009173	17226843	125322098	9255161	110038005172
110038011262	11102910	110038005135	17058140	124658053	11100260	110038201069
110038011222	11102912	110038007158	17226843	125322098	11101907	110038003094
110038011032	11101916	110038009023	830287	128036514	11102919	110038001156
110038120111	11103863	110038010086	A00011306	X02N70045	108357	110038008089
110038120057	11102412	110038010047	17083054	W00010712	11102912	110038006054

110038011019	11102910	110038007090	17058140	124658053	11101907	110038003201
110038120066	11102910	110038010017	17058140	124658053	11197314	110038008020
110038011284	11102910	110038006044	17058140	124658053	11199095	110038003214
110038120004	11102910	110038009226	17058140	124658053	11199095	110038004152
110038011289	11102910	110038006137	17058140	124658053	11101907	110038099125
110038120021	11102910	110038009266	17058140	124658053	11199095	110038004011
110038011300	11102910	110038006097	17058140	124658053	11199095	110038013046
110038011233	831148	110038007187			11102919	110038013006
110038011215	51557959	110038009214	2250783	50460184	101034031	110038007155
110038011261	11102910	110038008166	17058140	124658053	132483775	110038201127
110038011295	11102910	110038005224	17058140	124658053	11101907	110038003115
110038011267	11102912	110038009198	17226843	125322098	101034031	110038007117
110038011220	11102912	110038007140	17226843	125322098	60411048	110038201123
110044120817	11106002	110044228106	17062963	110505000771		
110012011345	11104859	110012006200	11189205	605		110012004181
110012011333	11103330	110012008103	A00011306	C00101542	11103560	110012099177
110012911461	11102010	1100120Y9301	2245673	60294138		
110012111077	11101929	110012023314	17056520	15972916		
110012011230	11103863	110012009245	A00011306	X02N70045	11103560	110012001138
110012111211	11104070	110012108193	17131025	17388911		
110012111177	11103543	110012108147	17131025	17278995		
110012011310	11108672	110012006225	17131025	17388911	11102390	11001200M752
110012111179	11102412	110012109239	17083054	W00010712	11103560	110012107197
110012011325	11104859	110012007137	11189205	605	10569537	110012005076
110012120029	11108672	110012009326	17131025	17388911	11105867	110012006028
110012911443	11199995	1100120Y8477	2167531	15088752		
110012120008	11108672	110012009036	17131025	17388911	11199095	110012006117
110012011343	11108541	110012009303	123645630	17213746	780	110012007224
110012912209	11102010	110012110710	2245673	60294138		
110012011274	11104859	110012008154	11189205	605	132483775	110012005042
110012711436	11199995	110012708429	2167531	15088752		
110012112090	11104701	110012010017	163863148	962089	61174187	110012008001
110012111189	11102412	110012109224	17083054	W00010712		
110012911389	11102010	1100120Y8469	2245673	60294138		
110012111187	11102412	110012109258	17083054	W00010712		
110012111205	11102412	110012109272	17083054	W00010712		
110012111171	11104070	110012108040	17131025	17388911	11104070	110012106109
110012111102	11101929	110012109070	17056520	15972916	11101927	110012107067
110012111168	11103543	110012108165	17131025	17278995		
110019011124	11104114	110019007456	17349617	129062082	131601389	110019003111
110019011596	11101930	110019005069	2294436	123260594	11101902	110019099246
110019120072	11109571	110019006172	60372887	134482565	11102019	110019004110
110064011284	11101906	110064005216	2297473	123884970	11197351	110064002125
110064011244	11101930	110064008083	2294436	123260594	9313518	110064006029

110064011246	11101930	110064005263	2294436	123260594	31103409	110064003196
110064120032	11101906	110064004229	2297473	123884970	11197351	110064001163
110064011191	11101930	110064004218	2294436	123260594	11196046	110064099132
110064011278	11199995	110064002138	2167531	15088752	23199202	110064097146
110064011245	11101930	110064006234	2294436	123260594	11100113	110064004252
110064011049	11101916	110064007020	830287	128036514	11100113	110064004269
110064011287	11101906	110064005120	2297473	123884970	11197351	110064099153
110064011304	11199995	110064009216	2167531	15088752	11106006	110064003145
110064011057	11101916	110064009044	830287	128036514	101034031	110064007033
110064011248	11101930	110064006166	2294436	123260594	11103828	11006400S148
110064011223	11103863	110064009235	A00011306	X02N70045	23107077	110064000S49
110064011281	11101906	110064006051	2297473	123884970	13203057	110064004027
110064011237	11101930	110064009165	2294436	123260594	11102912	110064007136
110064120020	11101906	110064005217	2297473	123884970	11197351	110064001031
110064011207	11101916	110064009056	830287	128036514	23206042	110064003272
110064011202	11103863	110064009243	A00011306	X02N70045	23107074	110064004148
110064011224	11109571	110064006102	60372887	134482565	11197314	110064004087
110064011189	11199995	110064002154	2167531	15088752	11194166	110064091026
110064011195	11103863	110064009220	A00011306	X02N70045	23107076	110064005121
110064011188	11103863	110064009200	A00011306	X02N70045	11199995	110064005186
110064011236	11101930	110064007168	2294436	123260594	11199995	110064002062
110064011286	11199995	110064009260	2167531	15088752	6120	110064005200
110064011227	11101930	110064007191	2294436	123260594	131601389	110064004143
110064120004	11101906	110064005032	2297473	123884970	11197351	110064001192
110064120022	11101906	110064006110	2297473	123884970	11100113	110064003021
110064011242	11101930	110064009046	2294436	123260594	11106002	110064005061
110064011300	11199995	110064005158	2167531	15088752	11197351	1100640S1029
110064120048	11101906	110064009080	2297473	123884970	11199095	110064007054
110064011307	11199995	110064003125	2167531	15088752	11196044	110064001066
110064011269	11101906	110064006113	2297473	123884970	11100113	110064002055
110064011238	11199995	110064005144	2167531	15088752	11197351	110064001084
110064120010	11199995	110064009217	2167531	15088752	11106006	110064005099
110064120008	11101906	110064007045	2297473	123884970	11100113	110064002045
110064011289	11199995	110064008226	2167531	15088752	129032447	110064006059
110064011213	11101930	110064008150	2294436	123260594	11199095	110064001136
110064011196	11103863	110064009226	A00011306	X02N70045	23107077	110064004212
110064011306	11101906	110064003084	2297473	123884970	11194161	110064000S27
110064011116	11101916	110064005252	830287	128036514	11197351	110064096164
110064011199	11101916	110064008121	830287	128036514	11199995	110064003055
110064120023	11101906	110064010008	2297473	123884970	131601389	110064007192
110064011295	11101906	110064004273	2297473	123884970	11197351	110064099107
110064011183	11199995	110064009133	2167531	15088752	11101916	11006400S159
110064120001	11101906	110064005163	2297473	123884970	11197351	110064001129
110064011256	11101930	110064008068	2294436	123260594	11100113	110064005043

110064011103	11101930	110064004245	2294436	123260594	11197351	110064002130
110064011265	11199995	110064008204	2167531	15088752	129032447	110064004148
110064011206	11103863	110064009209	A00011306	X02N70045	23206092	110064006117
110064011268	11101906	110064005187	2297473	123884970	11194138	110064003007
110010120052	11108541	110010010064	123645630	17213746	101034031	110010208092
110010011029	11108541	110010208289	123645630	17213746	53206068	110010204156
110010011100	11108549	110010207227	123645630	17213746	7491	110010204268
110010011232	11104701	110010109130	163863148	962089	11106004	110010207225
110010011181	11108541	110010208381	123645630	17213746	11101927	110010206202
110010120009	11109751	110010206200	122358313	60871898	11101917	110010096146
110010W10296	11102910	110038006214	17058140	124658053	23104263	110038201070
110010W11006	11102910	110038003123	17058140	124658053	11196044	11003800M581
110010011034	11108541	110010206284	123645630	17213746	11197361	110010202336
110010011252	11102412	110010010056	17083054	W00010712	101034031	110010208053
110010011114	11108541	110010206164	123645630	17213746	11102910	110010202072
110010011174	11108549	110010208239	123645630	17213746	11101927	110010204012
110010011108	11108549	110010208464				
110010120043	11108541	110010205156	123645630	17213746		110010099324
110010011160	11108549	110010207354	123645630	17213746	11101927	110010203016
110010011230	11104701	110010206180	163863148	962089		
110010011220	11104701	110010207148				
110010011227	11104701	110010208344	163863148	962089	11199090	110010204080
110010011251	11102412	110010010036	17083054	W00010712	11104070	110010203294
110010120003	11108541	110010208468	123645630	17213746	11101929	110010206256
110010011185	11108541	110010109131	123645630	17213746	11106003	110010205218
110010011239	11104701	110010206068	163863148	962089	11101929	110010204080
110010011215	11108541	110010109059	123645630	17213746	129032447	110010205314
110010011184	11108541	110010207409	123645630	17213746	31104707	110010202306
110010010201	11108541	110010205054	123645630	17213746	31102005	110010000M24
110010011112	11108541	110010206076	123645630	17213746	11101929	110010203358
110010120006	11108541	110010010010	123645630	17213746	11100519	110010200307
110010120005	11102412	110010010021	17083054	W00010712	11104070	110010206136
110010011110	11108541	110010208098	123645630	17213746	36105819	110010201324
110010011158	11108549	110010208206	123645630	17213746	9313518	110010303097
110010011116	11108541	110010109089	123645630	17213746	11199090	110010207144
110010011226	11108541	110010208412	123645630	17213746	11199090	110010206094
110071011387	11109571	110071005594	60372887	134482565	HO1104668	110071005240
110071011098	11101916	110071007427	830287	128036514	11101930	110071005117
110071011427	11101915	110071006091	2283503	17348883	11194160	110071003065
110071011356	11101917	110071005141	17099649	17394861	11196035	110071002035
110071011491	11101915	110071004135	2283503	17348883	11196035	110071002063
110071011417	11101915	110071005298	2283503	17348883	11100519	110071002239
110071011422	11101915	110071006316	2283503	17348883	11101916	110071004150
110071011413	11104070	110071008465	17131025	17388911	11199090	110071002361

110071011416	11101917	110071005339	17099649	17394861	11197351	110071099122
110071011495	11101915	110071008341	2283503	17348883	11199090	110071099155
110071011344	11101917	4414	17099649	17394861		
110071011388	11109571	110071008176	60372887	134482565	9255254	110071004601
110071011336	11101915	110071005560	2283503	17348883	A00011322	110071004548
110071011466	11101930	110071006418	2294436	123260594	11196035	110071001022
110071011488	11104070	110071009363	17131025	17388911	23107073	110071005426
110071011316	11101915	110071009249	2283503	17348883	11102919	110071007282
110071011103	11104070	110071007520	17131025	17388911	11100113	110071004089
110071011441	11101917	110071009288	17099649	17394861	11104070	110071004612
110071120609	11102129	110071009061	2149849	14142293	101034031	110071007013
110071011412	11101917	110071007358	17099649	17394861	11100113	110071004162
110071011219	11101915	110071007035	2283503	17348883	11196035	110071004345
110071011431	11101929	110071004376	17056520	15972916		S06000722
110071011443	11101917	110071004413	17099649	17394861		S05026473
110071011415	11101917	110071009311	17099649	17394861	11199090	110071005631
110071010362	11104070	110071007354	17131025	17388911	11101916	110071005517
110071011429	11101930	110071008472	2294436	123260594	11199095	110071004141
110071011430	11101930	110071008472	2294436	123260594	11199095	110071004141
110071011349	11101917	110071008092	17099649	17394861	37405019	110071005184
110071010481	11108541	110071002025	123645630	17213746	11193546	11007100M787
110071120606	11103543	110071009134	17131025	17278995	101034031	110071007145
110071011433	11101929	110071006117	17056520	15972916	11197314	110071003309
110071011318	11101915	110071008041	2283503	17348883	11101916	110071003281
110043120037	11101906	110043009425	2297473	123884970	11101929	110043004172
110043120123	11100260	110043009175	2149849	14142293	11101916	110043005048
110043011328	11102909	110043006193	2282997	60141550	37203032	110043099075
110043011298	11103832	110043006189	A00011306	H00896745	11196100	110043002198
110043120025	11101906	110043008298	2297473	123884970	11101927	110043004164
110043011330	11103863	110043009377	A00011306	X02N70045	23107072	110043007203
110043011291	11103863	110043009329	A00011306	X02N70045	132795034	110043005154
110043z12009	11103330	110043008226	A00011306	C00101542	11103560	110043006170
110043120109	11100260	110043009076	2149849	14142293	11106005	110043006286
110043011296	11103832	1100430Z6193	A00011306	H00896745	11102919	1100430B1093
110043120061	11101906	110043008055	2297473	123884970		110043006026
110043011380	11103863	110043009396	A00011306	X02N70045	132795034	110043004190
110043120016	11101906	110043010075	2297473	123884970	11101929	110043006259
110043Z11133	11104859	110043007217	11189205	605	15105497	110043002159
110043Z10029	11103543	110043108263	17131025	17278995		
110043011416	11103543	110043006199	17131025	17278995	50927	110043003124
110043120121	11100260	110043009374	2149849	14142293	831070	110043000H47
110043120008	11103543	110043010024	17131025	17278995	11101908	1100430Z6100
110043011337	11101929	1100430000Z0	17056520	15972916		
110043011294	11103863	110043009326	A00011306	X02N70045	132795034	110043098079

110043120158	11100260	110043003129	2149849	14142293	11196018	110043000104
110043011108	11108541	110043009112	123645630	17213746	11101908	110043005141
110043011117	11103863	110043009106	A00011306	X02N70045	11101908	110043002234
110043120046	11101906	110043008286	2297473	123884970	11101927	1100430Z6124
110043011396	11103543	110043008257	17131025	17278995	11100519	110043003102
110043120017	11103543	110043008201	17131025	17278995	11100519	110043006136
110043Z11072	11104859	110012005188	11189205	605	11100260	110012099121
110043011302	11103832	110043008241	A00011306	H00896745	9313518	1100430Z6094
110043011304	11103832	110043008121	A00011306	H00896745	9255254	110043001146
110043Z11103	11103863	110043009274	A00011306	X02N70045	11102910	1100430Z6013
110043120130	11100260	110043009104	2149849	14142293	101034031	110043007066
110043120024	11103543	110043010033	17131025	17278995	295614274	110043003188
110043011288	11103832	1100430Z7087	A00011306	H00896745	14104023	1100430X4034
110043120011	11103543	110043008353	17131025	17278995	11100519	110043006250
110043120029	11103543	110043010069	17131025	17278995	130411853	110043007228
110043011303	11103832	1100430Z7075	A00011306	H00896745	13104525	1100430Y3054
110043011418	11103543	110043006101	17131025	17278995	11100519	110043000203
110043Z11127	11104859		11189205	605		
110043120104	11100260	110043010112	2149849	14142293	11101908	110043007072
110043011402	11103543	110043007214	17131025	17278995	23105295	110043004151
110043011386	11103863	110043009382	A00011306	X02N70045	23107072	110043004013
110043011327	11103863	110043009356	A00011306	X02N70045	39HO00780	110043007239
110043Z10017	11104123	1100430Q8510	124003312	17278995		
110043120110	11101906	110043010098	2297473	123884970	11101929	110043006055
110043Z11169	11103863	110043009292	A00011306	X02N70045	11102910	110043006071
110043120082	11101906	110043007231	2297473	123884970	13104153	110043005214
