# Supplementary material for: Associations between polymorphisms of SLC22A7, NGFR, ARNTL and PPP2R2B genes and Milk production traits in Chinese Holstein
Source: BMC Genom Data. 2021 Nov 3;22:47. doi: 10.1186/s12863-021-01002-0 (PMC8567656; doi:10.1186/s12863-021-01002-0)
Supplement: Supplementary file 5 — Additional file 5: Table S5. Primers and procedures for PCR used in SNPs identification of SLC22A7, NGFR, ARNTL and PPP2R2B genes. [file 12863_2021_1002_MOESM5_ESM.pdf]

Additional file 5: Table S5 Primers and procedures for PCR used in SNPs identification of *SLC22A7*, *NGFR*, *ARNTL* and *PPP2R2B* genes.

Table S5-1 Details of primers.

**Primer Name**	**Primer Sequence (5'-3')**	**Product Length (bp)**	**Annealing Temp. (℃)**
SLC22A7-1F	GCTCTTTGTGACTGCCTTCC	790	60
SLC22A7-1R	AGCTAGGCTGGACTTCCTGA		60
SLC22A7-2F	TCACTTCCCATCATCACCAC	822	60
SLC22A7-2R	TCCTAACCAGGTGAAGGGTTC		60
SLC22A7-3F	CCTGGGACATACTCTCCAGGT	531	60
SLC22A7-3R	TGATGAGCAGAGAGCCTTCA		60
SLC22A7-4F	GGGGAACTAAGCCAGACAGG	732	60
SLC22A7-4R	GGTAAACGGAGACTCTCAAAGG		60
SLC22A7-5F	CACCCCTGAGACTCGTAACC	334	60
SLC22A7-5R	AAACCTGCACAGGGGTCAC		60
SLC22A7-6F	TAGAGGGGCCAATACGGAGT	362	60
SLC22A7-6R	TTGGAGGCAGGAAGACAGTT		60
SLC22A7-7F	CATTGTGATGCCACTGGGTA	388	60
SLC22A7-7R	CCTCTGAGACACCTGGGAAG		60
SLC22A7-8F	CCAGGCTGAGGGTACTGTGT	401	60
SLC22A7-8R	GGCCAGTAGGACATCCTGAA		60
SLC22A7-9F	GCAGCAGAGGACAGAGATCC	341	60
SLC22A7-9R	CAGAGTCCAATCCCTCCAGA		60
SLC22A7-10F	ATTATGGGGACTGGGAAGGT	684	60
SLC22A7-10R	ACTAACAAGCCCTCCCTCCA		60
SLC22A7-11F	AGCTGTCTGGGGAGAAGG	460	60
SLC22A7-11R	CCATGCACACTCGCATACAT		60
SLC22A7-12F	GTGGTTGGCTGAGAGGCTAA	520	60
SLC22A7-12R	ATCCTGTGGTGAGGTCTGCT		60
SLC22A7-13F	TGACTTCTGGGTGACCTTGG	563	60
SLC22A7-13R	TCTCTGCCAACTCCTCCTTC		60
SLC22A7-14F	GCGATTGGAAAGACCAAGAG	766	60
SLC22A7-14R	GCAAGTGGGTCTTCCTGTTC		60
SLC22A7-15F	CCCGTAGAACATCCCAAACT	508	60
SLC22A7-15R	CAGCAGAGCTTGCATTTGAC		60
SLC22A7-16F	GATAATGCCATCTGCCCAGT	521	60
SLC22A7-16R	GTCCTCTTCCCTCTGCTTGT		60
NGFR-1F	CGCAGTTCACAGCGTCTC	820	60
NGFR-1R	CCTTTCCTGGTTCCCAGAC		60
NGFR-2F	CACACCTGGCCAAGTTCAG	500	60
NGFR-2R	TCGTGTGCAGTCATCTCCAT		60
NGFR-3F	TGTGTGTGTGCACTGTGTGA	509	60
NGFR-3R	CCCAGCCGTTATCAGCAGTA		60

NGFR-4F	AGGCCTTTGCAGAGAGAACC	326	60
NGFR-4R	AGAGGCGGAAAGGGACAT		60
NGFR-5F	ATGTCCCTTTCCGCCTCT	420	60
NGFR-5R	ACCCCAACGCTCTTTTCTGT		60
NGFR-6F	CCCTGTCAGCTTTCAAGTGC	441	60
NGFR-6R	CTTCTGGCACCCATGCAGT		60
NGFR-7F	GCGACTAAATTCGGGGTGT	584	60
NGFR-7R	GCCCAGAGAGGCTAACAGTG		60
NGFR-8F	CCACACCTGTACGTGTAGCC	500	60
NGFR-8R	CTTGGTTTGGGGCTGAGTTA		60
NGFR-9F	CGCCAGAGCACAGACACTT	323	60
NGFR-9R	CCTCTGCAGTCCCTCCTACA		60
NGFR-10F	TCCCCAGACTGAGAGGAGAA	831	60
NGFR-10R	TTCCCCGAGTTCACAGTGTAG		60
NGFR-11F	GCTTCTCGCCCTGTTTCTC	461	60
NGFR-11R	ATTGGGTTTGGCTGTGAGAC		60
NGFR-12F	CCTCTGGCAAGTGTCTCACA	502	60
NGFR-12R	CTCAAGCCTCAGTCGTCCTT		60
NGFR-13F	GCAGCATGCAGATTCAGAGA	802	60
NGFR-13R	AGGGACTGGGCAAAAAGG		60
NGFR-14F	GGGTTGGAGGTTGCAAGTAT	466	60
NGFR-14R	CAAGGTCTGGCAGCTAAGG		60
NGFR-15F	CCCCACATAATGGGGAAGTT	443	60
NGFR-15R	GGAAGTCTGCAACTCCTGGAC		60
NGFR-16F	CTGGTCCAGGAGTTGCAGA	742	60
NGFR-16R	CTCTCTCTTCAGGGCCTTTG		60
NGFR-17F	TGAGGCTAAGCCCTACTGAAG	804	60
NGFR-17R	GGGATAGCATGTGCAAAGGT		60
ARNTL-1F	CAAACACCATAAGCAAAGATAAAAG	735	54
ARNTL-1R	AAACATGAAACATCCATAACCAATC		54
ARNTL-2F	ATTTATTGATGGATTAGATACAGGC	753	54
ARNTL-2R	TCCGCATTATCTTGCATCG		56
ARNTL-3F	GGAATGATGGGGACAAAAGCCTGAT	845	61
ARNTL-3R	CCCAATGAAAGCTCTTGTCAATCTG		59
ARNTL-4F	ACCAGAACATAAACAGCAGAAGCAG	720	52
ARNTL-4R	TCTAGTCTTCTCTGCCTGTCCTTCT		59
ARNTL-5F	TTTGAGCTGACTTGCTTTGCACTTT	743	57
ARNTL-5R	TCACTGATTGTTGAAGAAATGTCCA		56
ARNTL-6F	CCTAGTCCTCCACCACCTTAGAAAA	259	61
ARNTL-6R	ACACCAAGTTTAGCCAGAGGAAGAC		61
ARNTL-7F	GTGGTGAGAAGCGATTTTGTATGTC	209	59
ARNTL-7R	CTTACAGCCTCCTTGCTTATCCCTA		61
ARNTL-8F	GCAGGGAAGAAAATGGATGTGTAAC	244	59
ARNTL-8R	CTCCCTAAGGACACGACAGTATCAA		61

ARNTL-9F	TGCCCTTCAAGTCCTCCAC	693	60
ARNTL-9R	AGCTTGCTAACGCCGATT		54
ARNTL-10F	GGAAGGTTTGAGGCAGTC	311	56
ARNTL-10R	AGTAAGAACAAGGTGGAGA		54
ARNTL-11F	CCGTGCTTTGGACGCTTAG	217	60
ARNTL-11R	GTGGGAACAGGGGCAGTA		58
ARNTL-12F	GCAGAGTAAAGGCAAAGC	304	54
ARNTL-12R	AGCACTTGTGACCCAGATA		56
ARNTL-13F	TGACAGACGACACGACCCT	348	60
ARNTL-13R	TGCAATCTGCCTGCTCTG		54
ARNTL-14F	ATCCCTCCCAGCCCTCCTCCTA	618	64
ARNTL-14R	GGCCCAGTCTGCTCTGCTTTAC		62
ARNTL-15F	GTAATAAGCCCTCTGAACCCACCCA	292	62
ARNTL-15R	GGGACACTTGAAACTTCAGACCTGG		62
ARNTL-16F	AGCGCATTTAGGATTTGG	348	52
ARNTL-16R	TAGCAGGATGACAGTAAAGGAT		54
ARNTL-17F	GAGCCACTCAGACACTTCATTT	409	56
ARNTL-17R	AACCAAGTTCCCAGCATTTC		53
ARNTL-18F	TGTGGCGTGCATGAGTAAG	676	58
ARNTL-18R	AGCCTCCCAGAGTTCCCT		58
ARNTL-19F	ACACGGCTACACCCATTC	428	56
ARNTL-19R	CTCCCACTTCCAGTATTTCACA		56
ARNTL-20F	ATCCAATAAGAAACCGAAGCCACCT	293	59
ARNTL-20R	GCCAGATGTTTTTTGCAAGCTCACT		59
ARNTL-21F	TCTTTATCTCCTCCCACA	373	52
ARNTL-21R	AGCAGGCTTTAGTTCCAC		54
ARNTL-22F	ACTCACCAGTCTTTATCTCCTCCCA	661	61
ARNTL-22R	AACTAATACATTATACTTTAACCCA		51
PPP2R2B-1F	GCACCAGAACAAACATCAGG	476	60
PPP2R2B-1R	TAGGCCCAGACAGGCTACAC		60
PPP2R2B-2F	CAGCCCTGTGTAGTCTTAGAGGA	340	60
PPP2R2B-2R	TGCAAGGAGGGTACTACGAGA		60
PPP2R2B-3F	CACCTGTGAAGTGGGGTTCT	704	60
PPP2R2B-3R	GGCAGCAGTGGCAGAATAC		60
PPP2R2B-4F	CTCCCCAAATTTGATGATGC	628	60
PPP2R2B-4R	GGTGACTGCCCCTTTTCAT		60
PPP2R2B-5F	TCTCCCCTCTGACAAACTGC	612	60
PPP2R2B-5R	TGCTATTCTGGGATGTGTGG		60
PPP2R2B-6F	GCAAGGACAGAGACCCATTT	744	60
PPP2R2B-6R	GCAGAAGGCAGTCTCGGTAG		60
PPP2R2B-7F	AGCATCCTCGCTGGGAAT	400	60
PPP2R2B-7R	CTCAGCGATCCGCAACTT		60
PPP2R2B-8F	GTCTGAAGAAAGGCGGTGAG	518	60
PPP2R2B-8R	AAAATGGTGCCTTTCTGGAC		60

PPP2R2B-9F	GGATTTTGACAGGCCAAGAG	334	60
PPP2R2B-9R	ATGGATTTGAGTCCCACAGC		60
PPP2R2B-10F	AATTCTGGCACTGGGAGCTT	351	60
PPP2R2B-10R	AGGCAGGAGCAGTCGTACAG		60
PPP2R2B-11F	AGCCAGCCTTGTGAAGTTGT	401	60
PPP2R2B-11R	CATGAGGGCAGCAGGAAC		60
PPP2R2B-12F	CCCACTTGGACATGGTCTG	395	60
PPP2R2B-12R	CCTGCAAGGTGAAAAGGAAA		60
PPP2R2B-13F	GCAGAGACAAAGCCCTAAGC	301	60
PPP2R2B-13R	CAAGGTTAAGGGCGACACAT		60
PPP2R2B-14F	CGTCCACTGACCTCCACTTT	344	60
PPP2R2B-14R	CACCCCTGAGACTCGTAACC		60
PPP2R2B-15F	CAGCATGTAATTGATCAGTCTCTC	462	60
PPP2R2B-15R	GGAAGAGTTCTCCACAAGTGC		60
PPP2R2B-16F	TGTCTGCCAGAGTCCTGATG	414	60
PPP2R2B-16R	GTGTGTCTGAGGTGGGGAAG		60
PPP2R2B-17F	CCCGATTCCAACCCTAAGTT	421	60
PPP2R2B-17R	CACTGACCTGCCTACCAGCTA		60
PPP2R2B-18F	CCCAGAACTATCTGAGTCAAGC	344	60
PPP2R2B-18R	CTCAGGGCCGATTCTGATG		60
PPP2R2B-19F	GCCCAAGAGCTTGTTTGTCT	723	60
PPP2R2B-19R	AAACACTGGACCCACCAGAG		60
PPP2R2B-20F	TTATTGCCCCGTTTTCTCTG	805	60
PPP2R2B-20R	GCCTTTACAGAGCAGCGTTT		60
PPP2R2B-21F	CCCCTTTCCTGGGTTTTCTA	823	60
PPP2R2B-21R	CCCATCAGGCCAGGAATC		60
PPP2R2B-22F	AAAACTGAGGGAGCATGTGG	757	60
PPP2R2B-22R	CTGTGTGCTGAAGGCATTGT		60

Table S5-2 Reaction system.

Content	Dosage	Total
Forward primer (10 pmol/μL)	1.25 μL	
Reverse primer (10 pmol/μL)	1.25 μL	
2×Taq Master Mix	12.5 μL	25 μL
DNA (50-100 ng/μL)	2 μL	
ddH_2O	8 μL	

Table S5-3 The procedures of PCR amplification.

Temperature (℃)	Time	Cycle No.
95	5 min	1
95	30 s	
Annealing Temp.	30 s	35
72	40 s	
72	10 min	1
